# Supplementary figures and images for: Brain-derived and in vitro-seeded alpha-synuclein fibrils exhibit distinct biophysical profiles
Source: eLife. 2024 Nov 25;13:RP92775. doi: 10.7554/eLife.92775 (PMC11588339; doi:10.7554/eLife.92775)

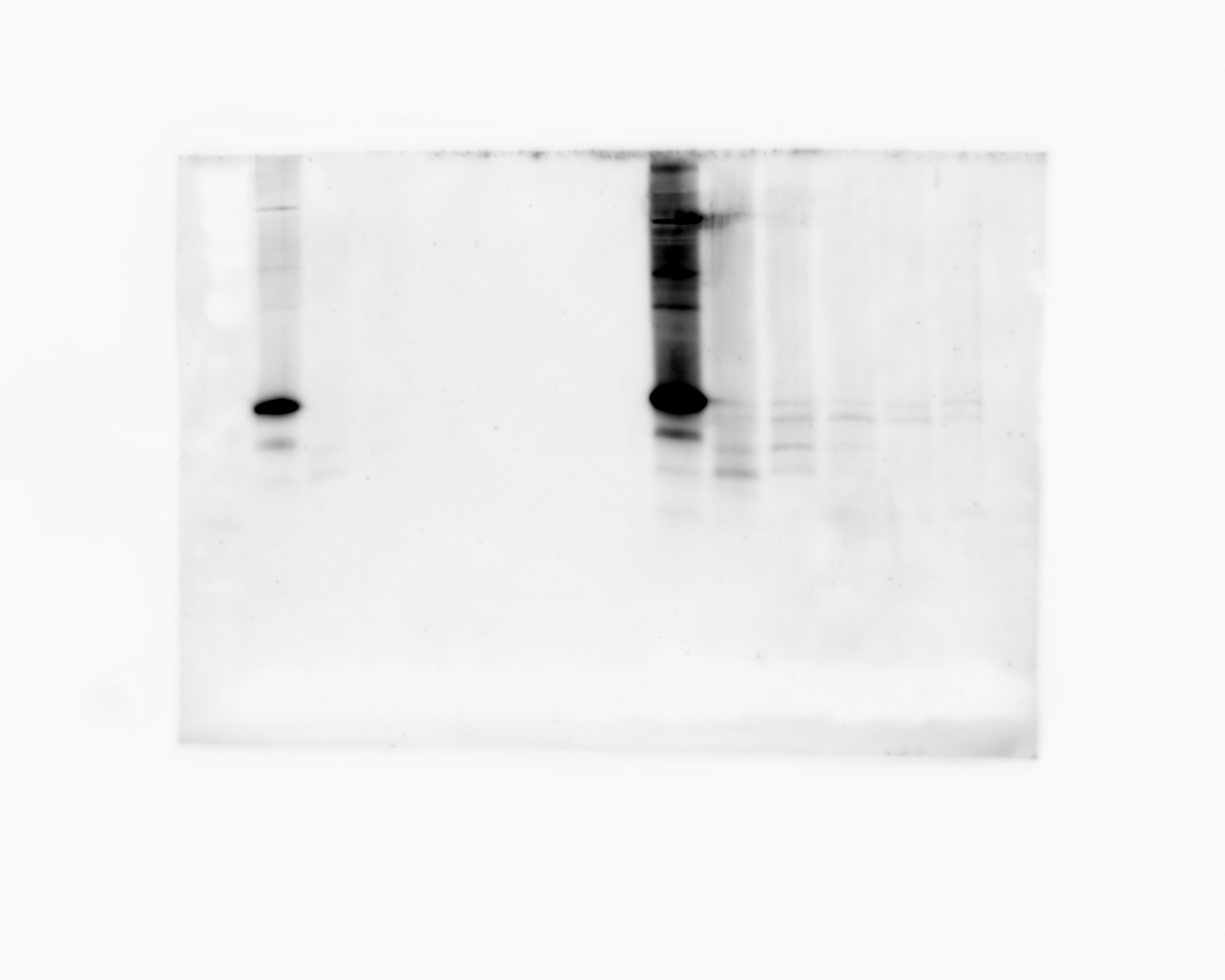

Supplement: Figure 3—source data 1. [file elife-92775-fig3-data1.zip › FIgure 3_Source data 1/Figure 3-PD-Brain derived.jpg]

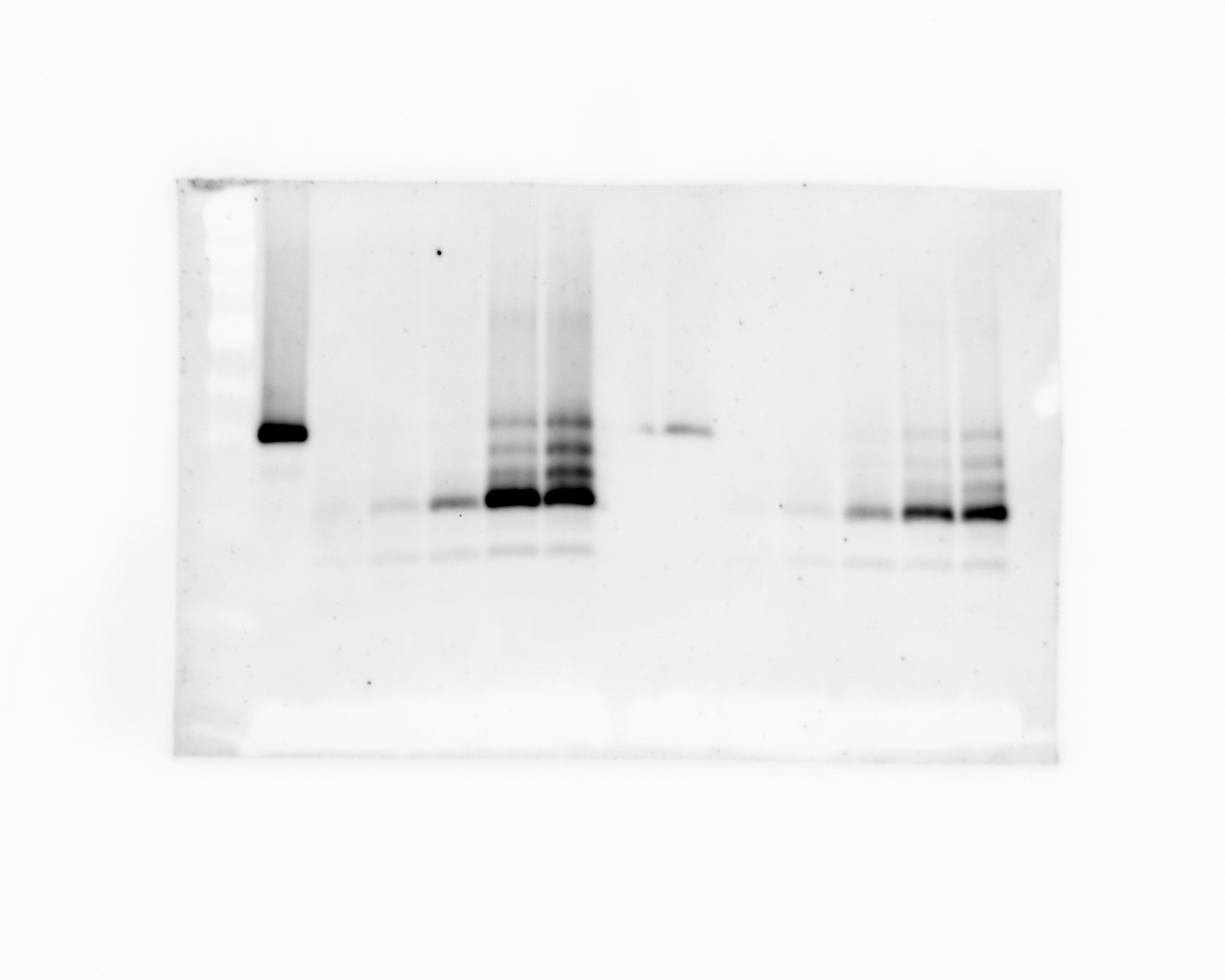

Supplement: Figure 3—source data 1. [file elife-92775-fig3-data1.zip › FIgure 3_Source data 1/Figure 3-DLB-SAA.jpg]

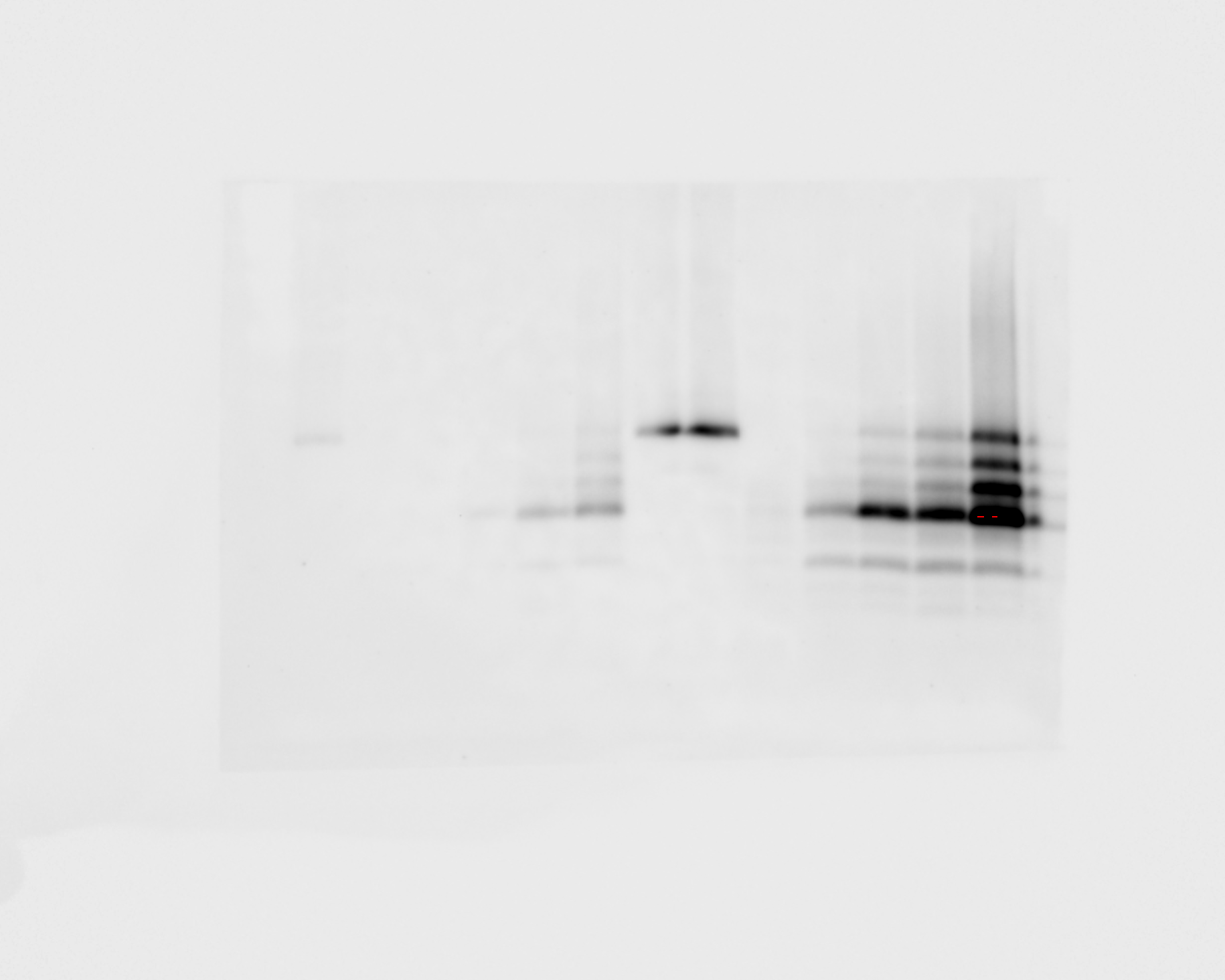

Supplement: Figure 3—source data 1. [file elife-92775-fig3-data1.zip › FIgure 3_Source data 1/Figure 3-PDD-SAA.tif]

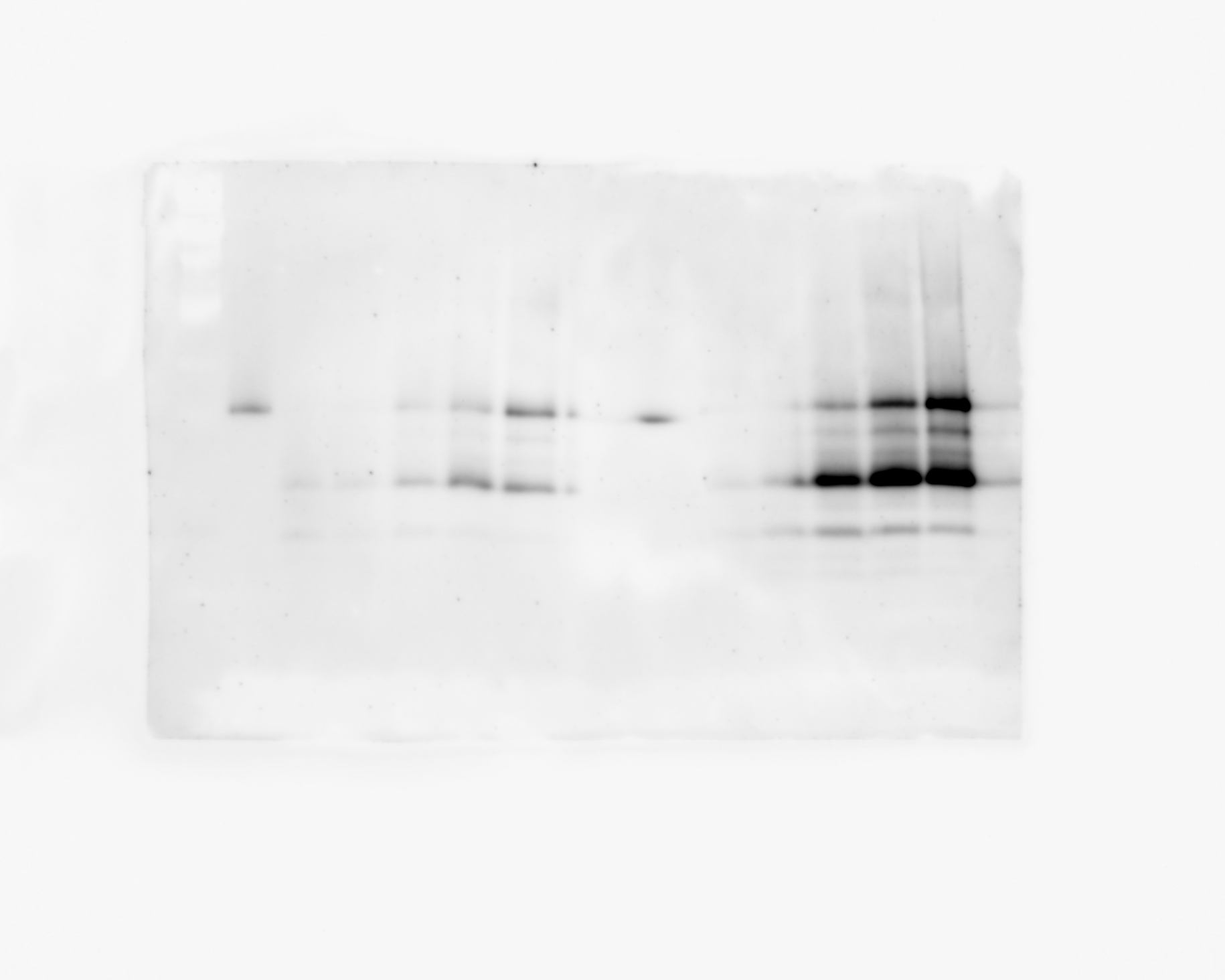

Supplement: Figure 3—source data 1. [file elife-92775-fig3-data1.zip › FIgure 3_Source data 1/Figure 3-MSA-SAA.jpg]

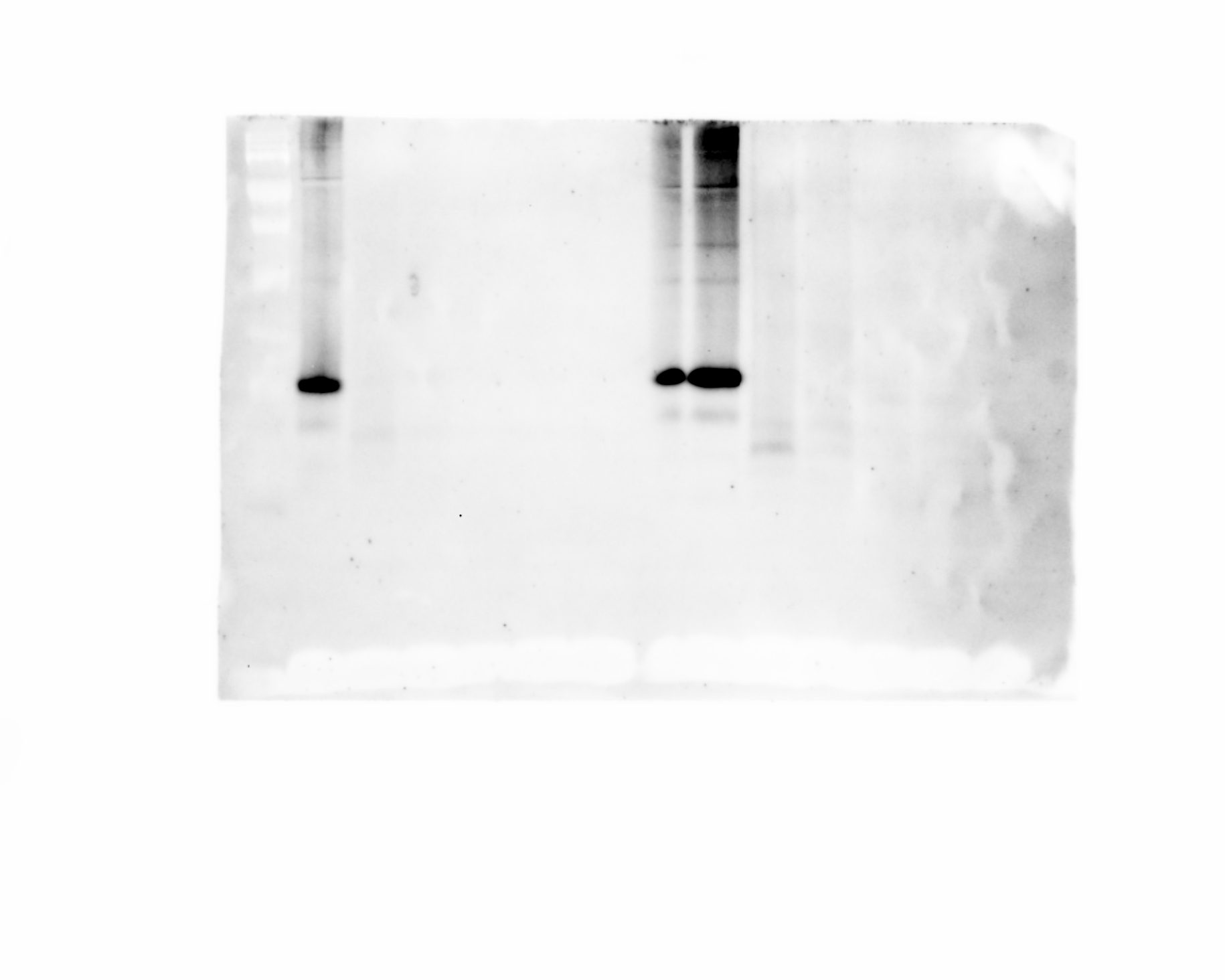

Supplement: Figure 3—source data 1. [file elife-92775-fig3-data1.zip › FIgure 3_Source data 1/Figure 3-MSA-Brain derived.jpg]

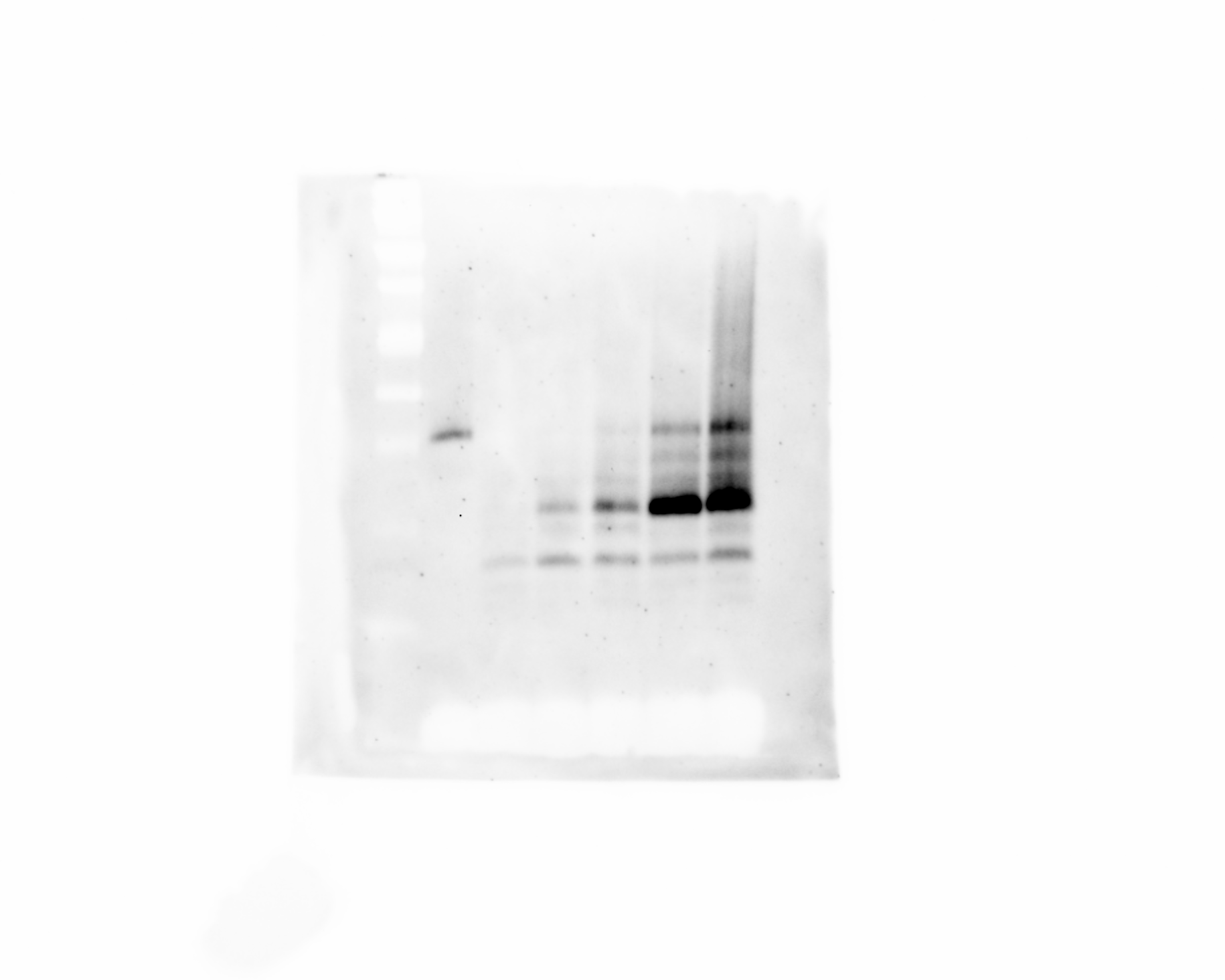

Supplement: Figure 3—source data 1. [file elife-92775-fig3-data1.zip › FIgure 3_Source data 1/Figure 3-PD-SAA.tif]

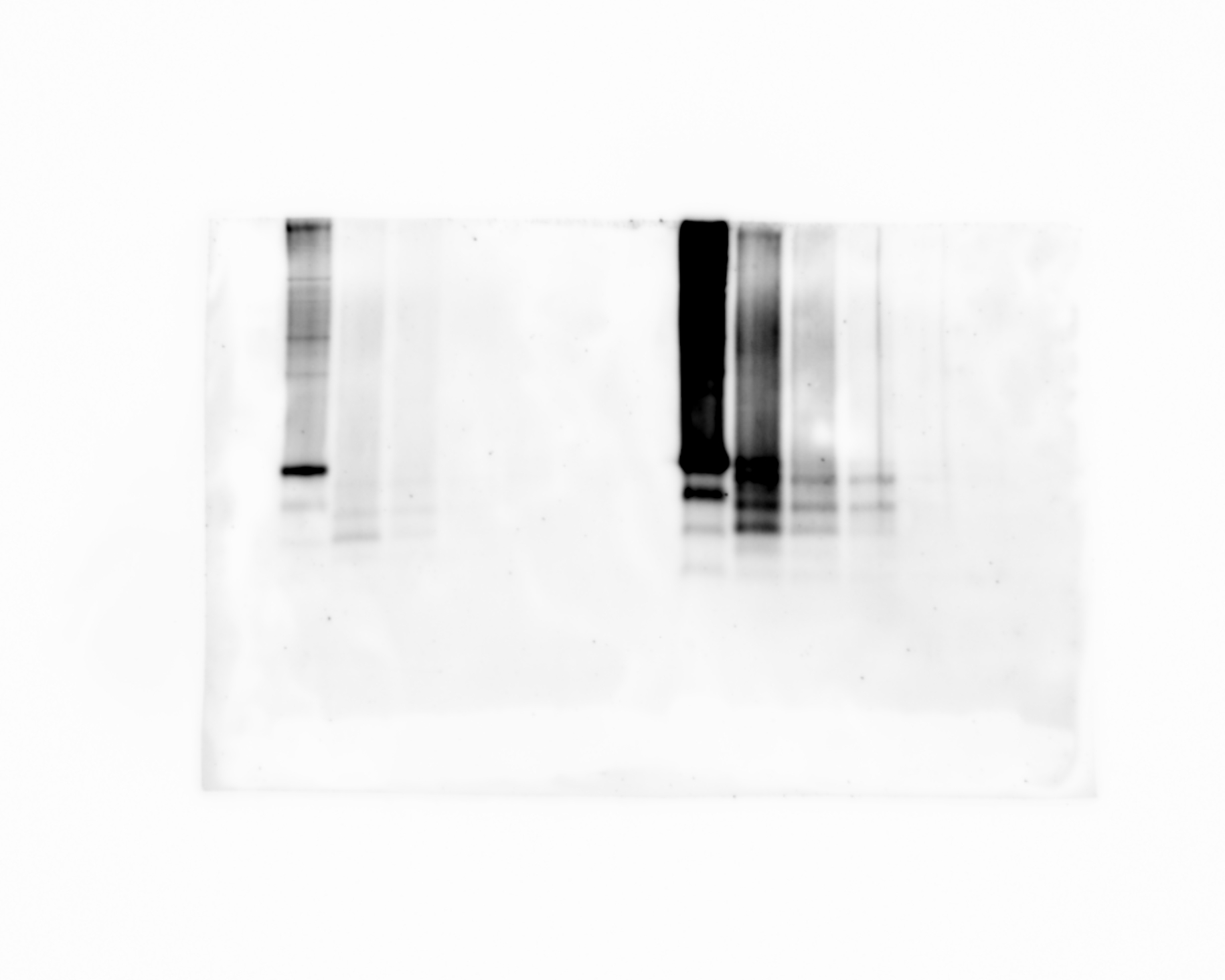

Supplement: Figure 3—source data 1. [file elife-92775-fig3-data1.zip › FIgure 3_Source data 1/Figure 3-DLB-Brain derived.jpg]

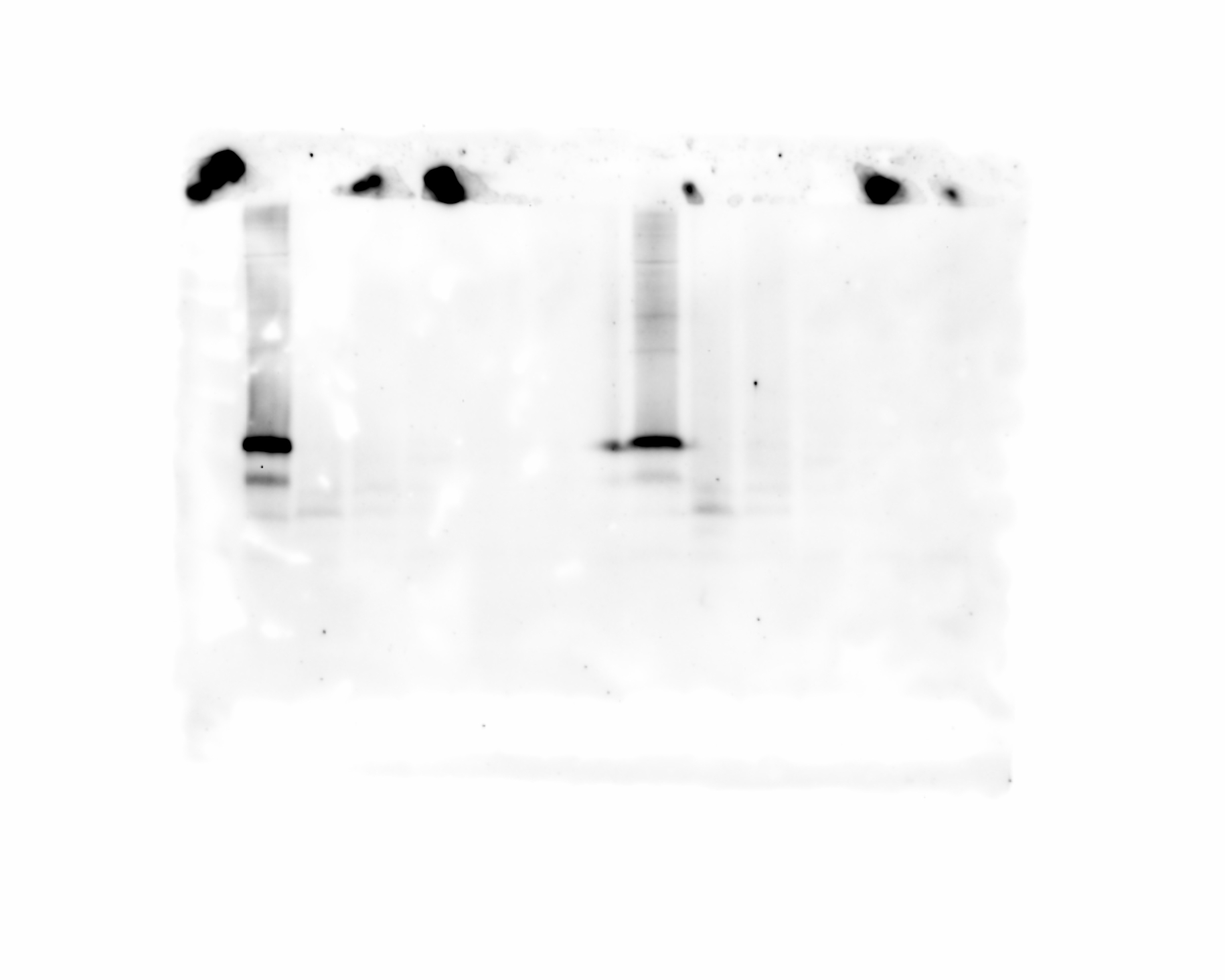

Supplement: Figure 3—source data 1. [file elife-92775-fig3-data1.zip › FIgure 3_Source data 1/Figure 3-PDD-Brain derived.jpg]

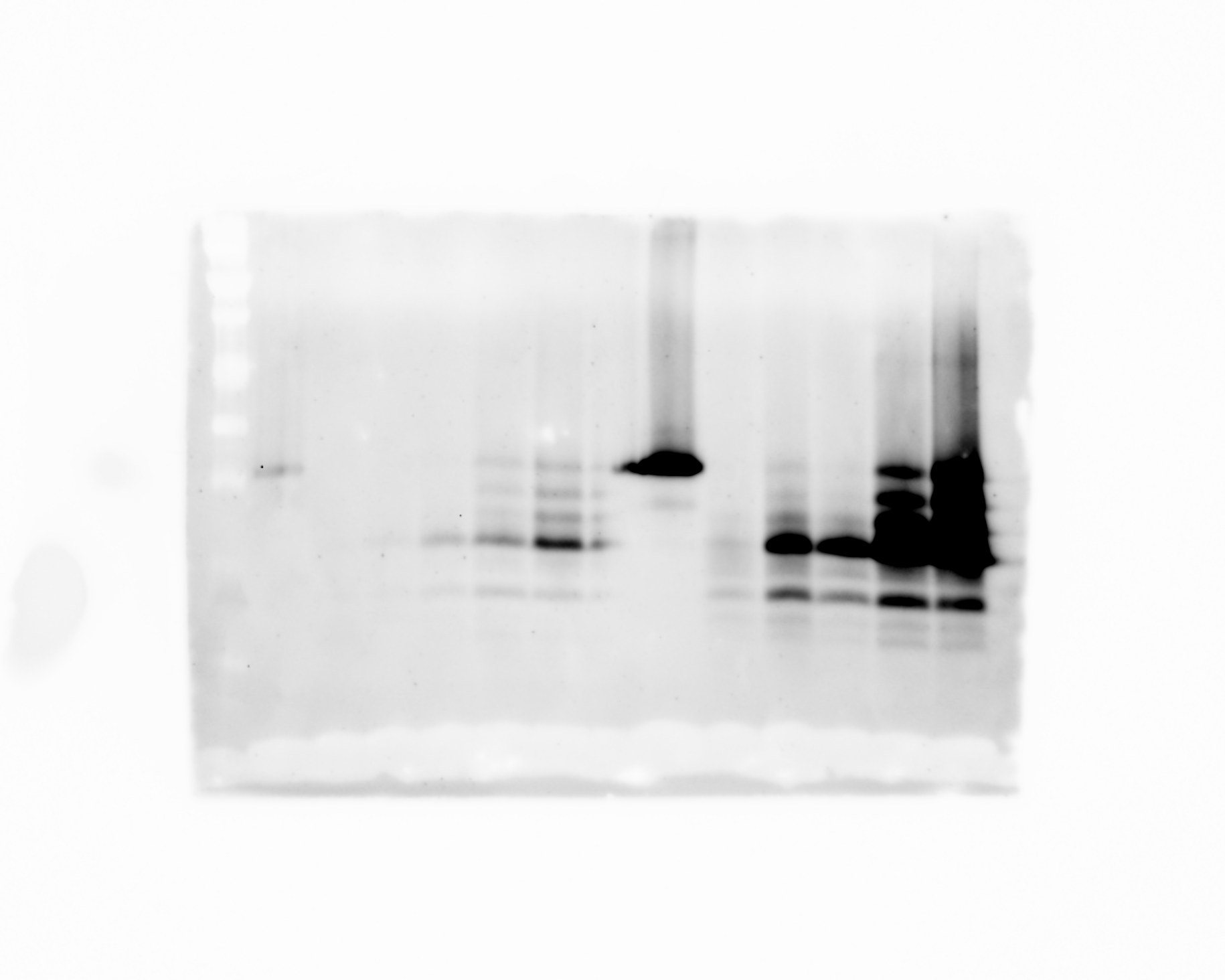

Supplement: Figure 3—figure supplement 1—source data 1. [file elife-92775-fig3-figsupp1-data1.zip › Figure 3 - figure supplement 1_Source data 1/Figure 3 - figure supplement 1 - A/A_PD1_SAA.tif]

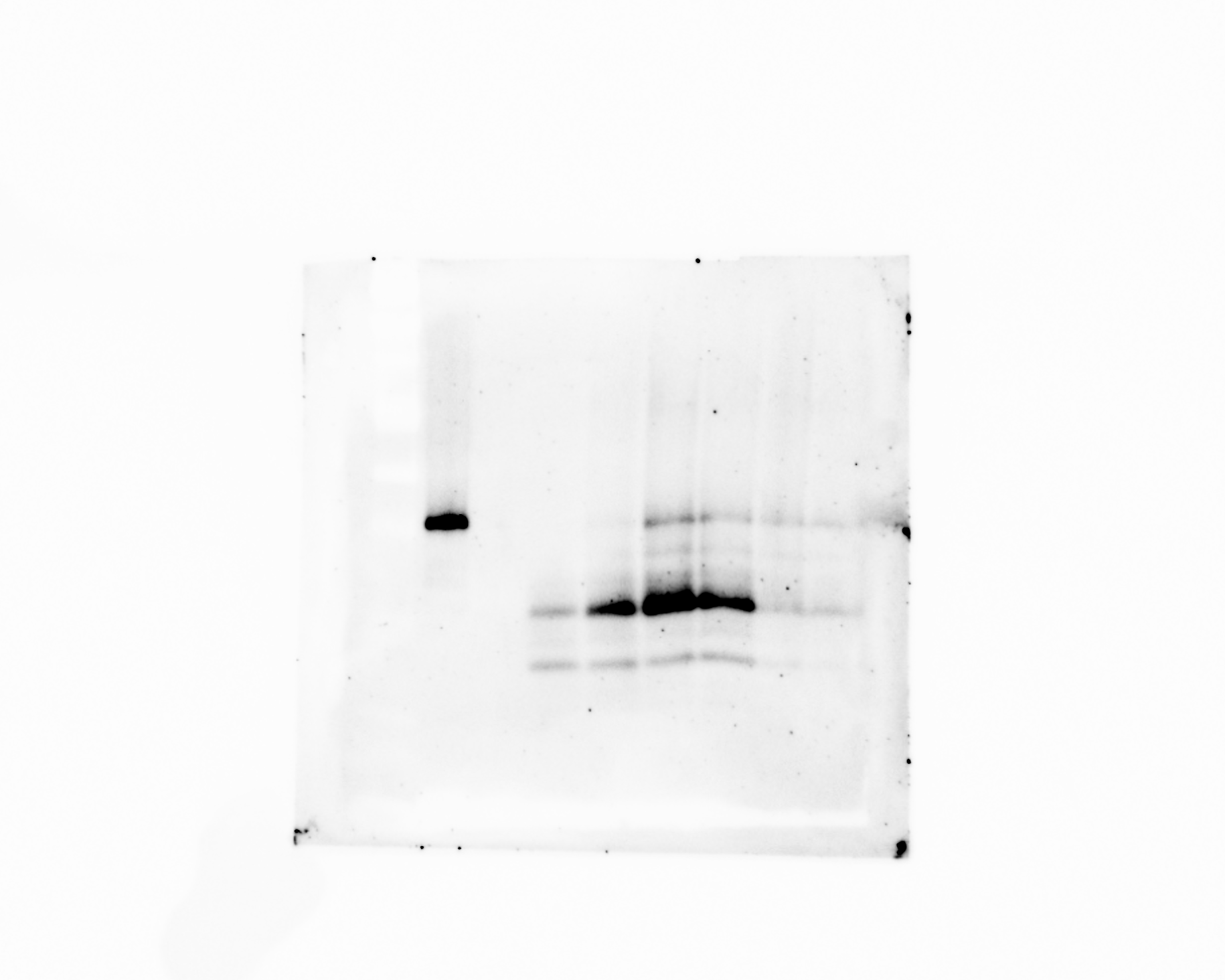

Supplement: Figure 3—figure supplement 1—source data 1. [file elife-92775-fig3-figsupp1-data1.zip › Figure 3 - figure supplement 1_Source data 1/Figure 3 - figure supplement 1 - A/A_PD3_SAA.tif]

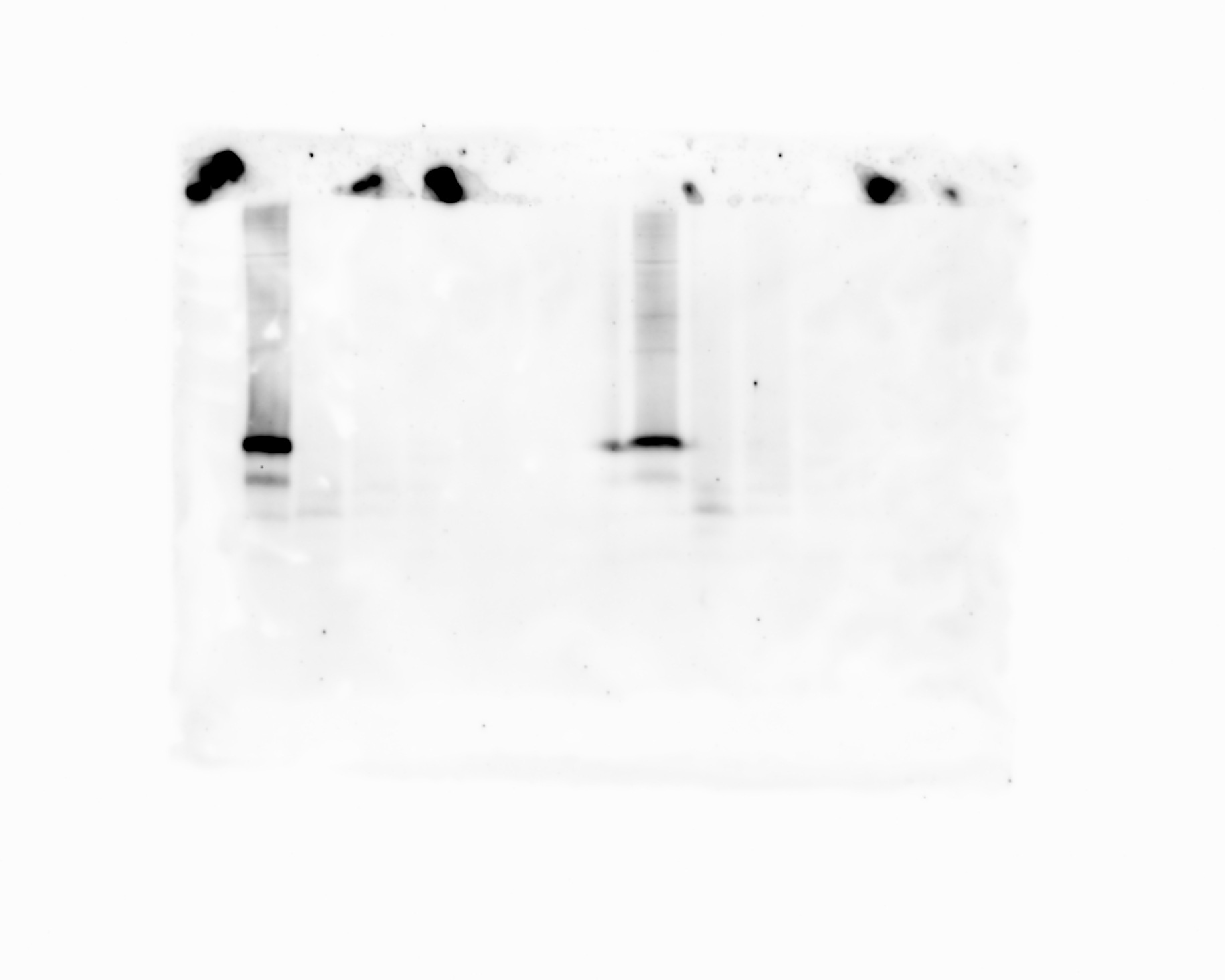

Supplement: Figure 3—figure supplement 1—source data 1. [file elife-92775-fig3-figsupp1-data1.zip › Figure 3 - figure supplement 1_Source data 1/Figure 3 - figure supplement 1 - A/A_PD2_BD.jpg]

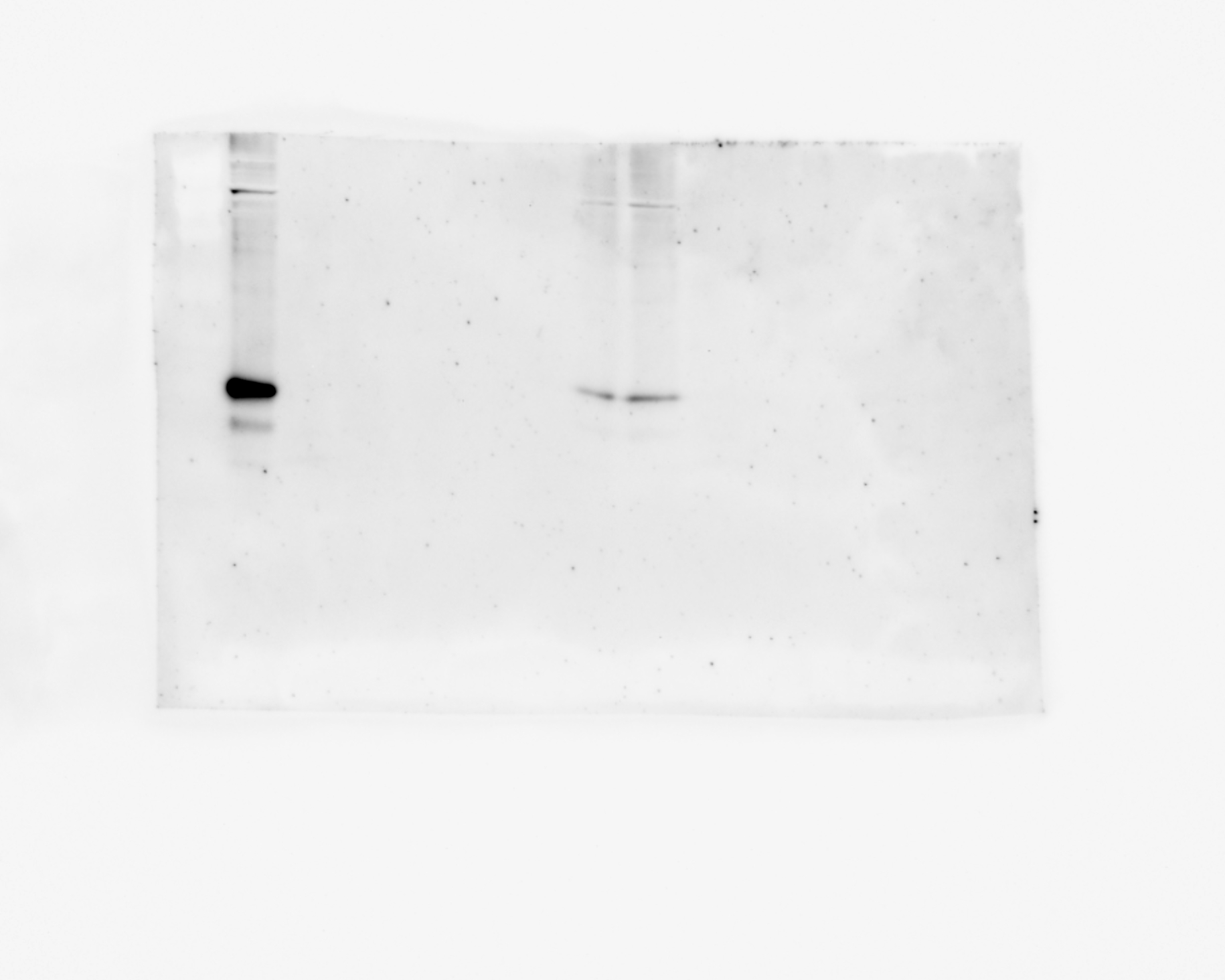

Supplement: Figure 3—figure supplement 1—source data 1. [file elife-92775-fig3-figsupp1-data1.zip › Figure 3 - figure supplement 1_Source data 1/Figure 3 - figure supplement 1 - A/A_PD1_BD.jpg]

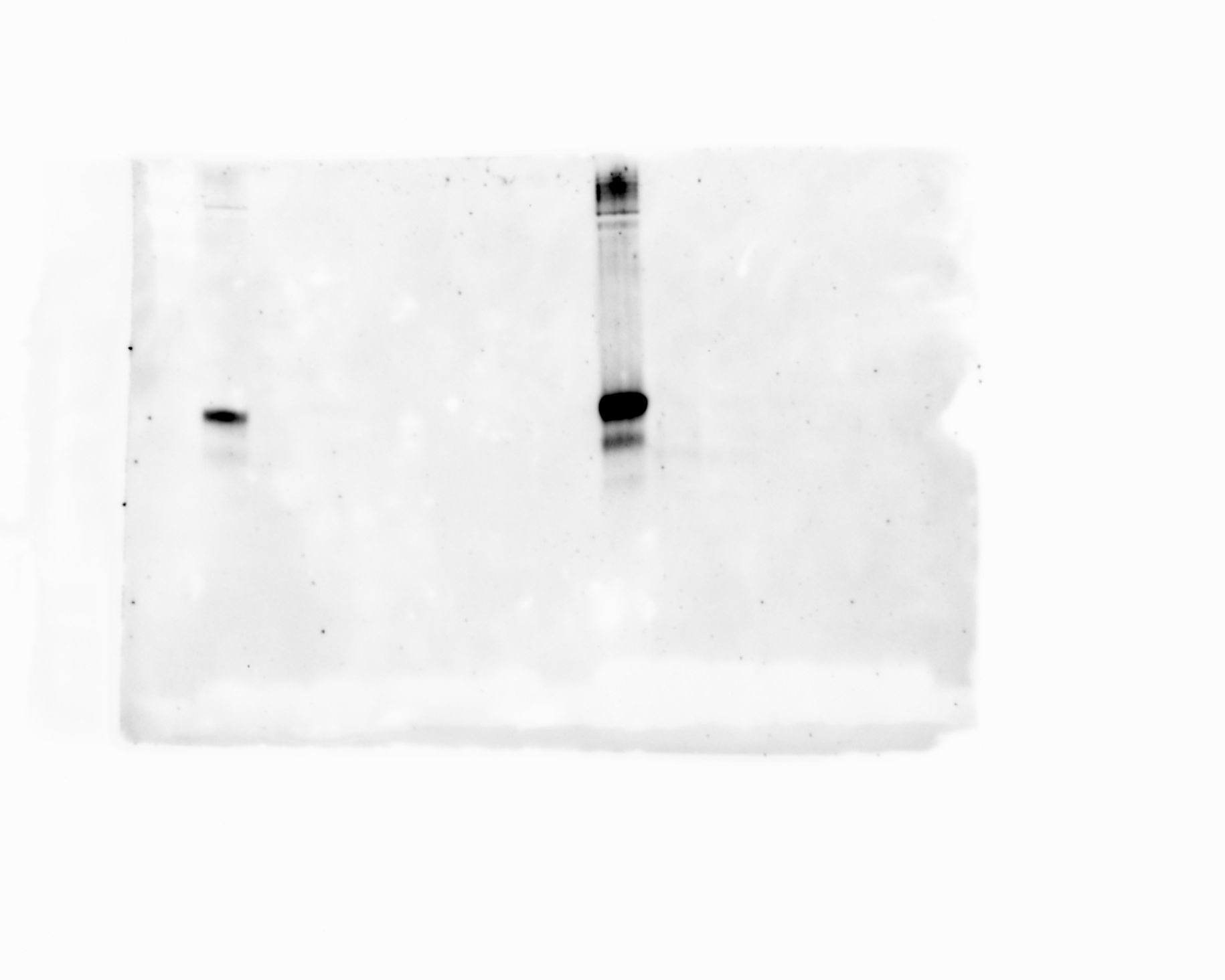

Supplement: Figure 3—figure supplement 1—source data 1. [file elife-92775-fig3-figsupp1-data1.zip › Figure 3 - figure supplement 1_Source data 1/Figure 3 - figure supplement 1 - D/D_MSA1_BD.jpg]

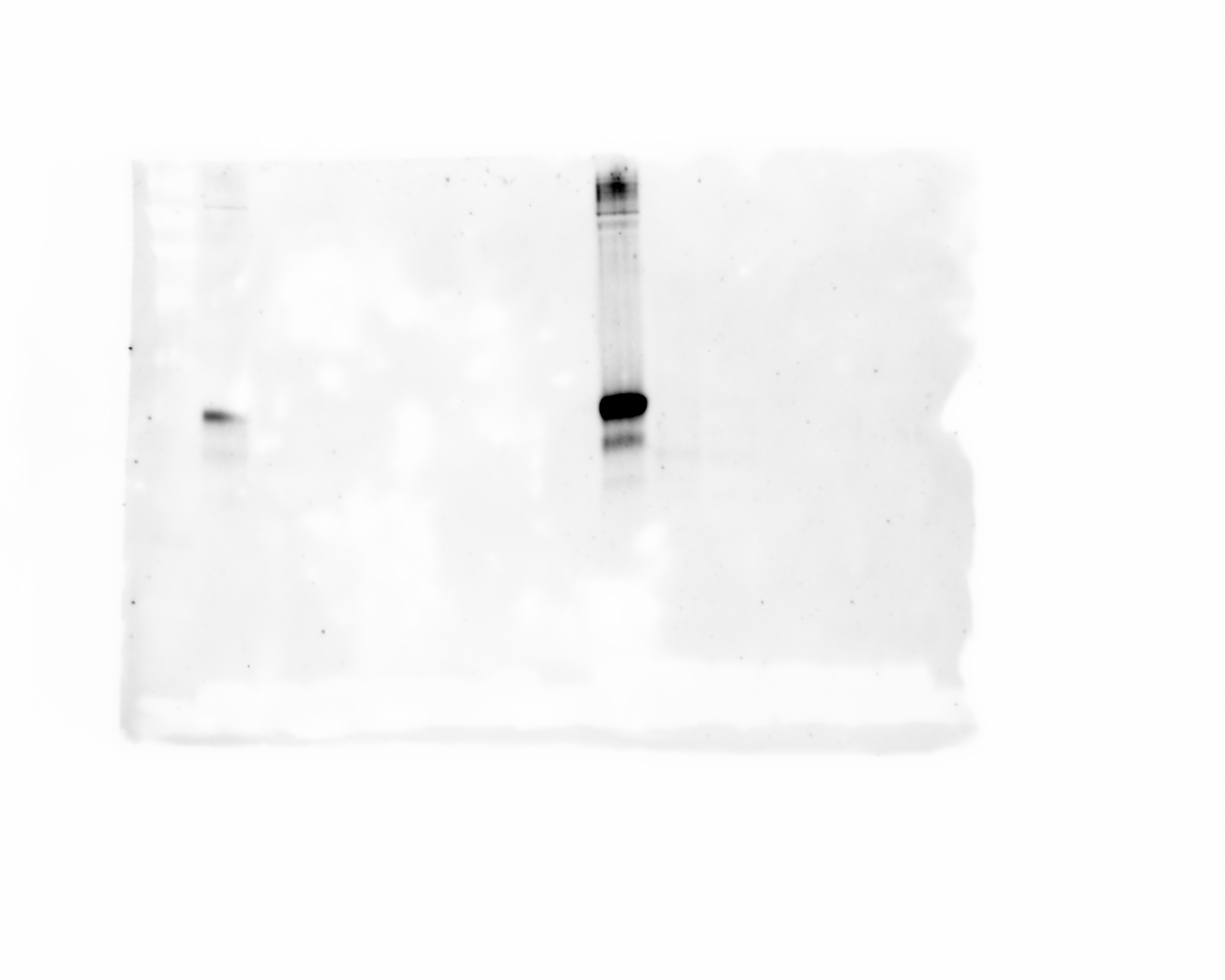

Supplement: Figure 3—figure supplement 1—source data 1. [file elife-92775-fig3-figsupp1-data1.zip › Figure 3 - figure supplement 1_Source data 1/Figure 3 - figure supplement 1 - D/D_MSA2_BD.jpg]

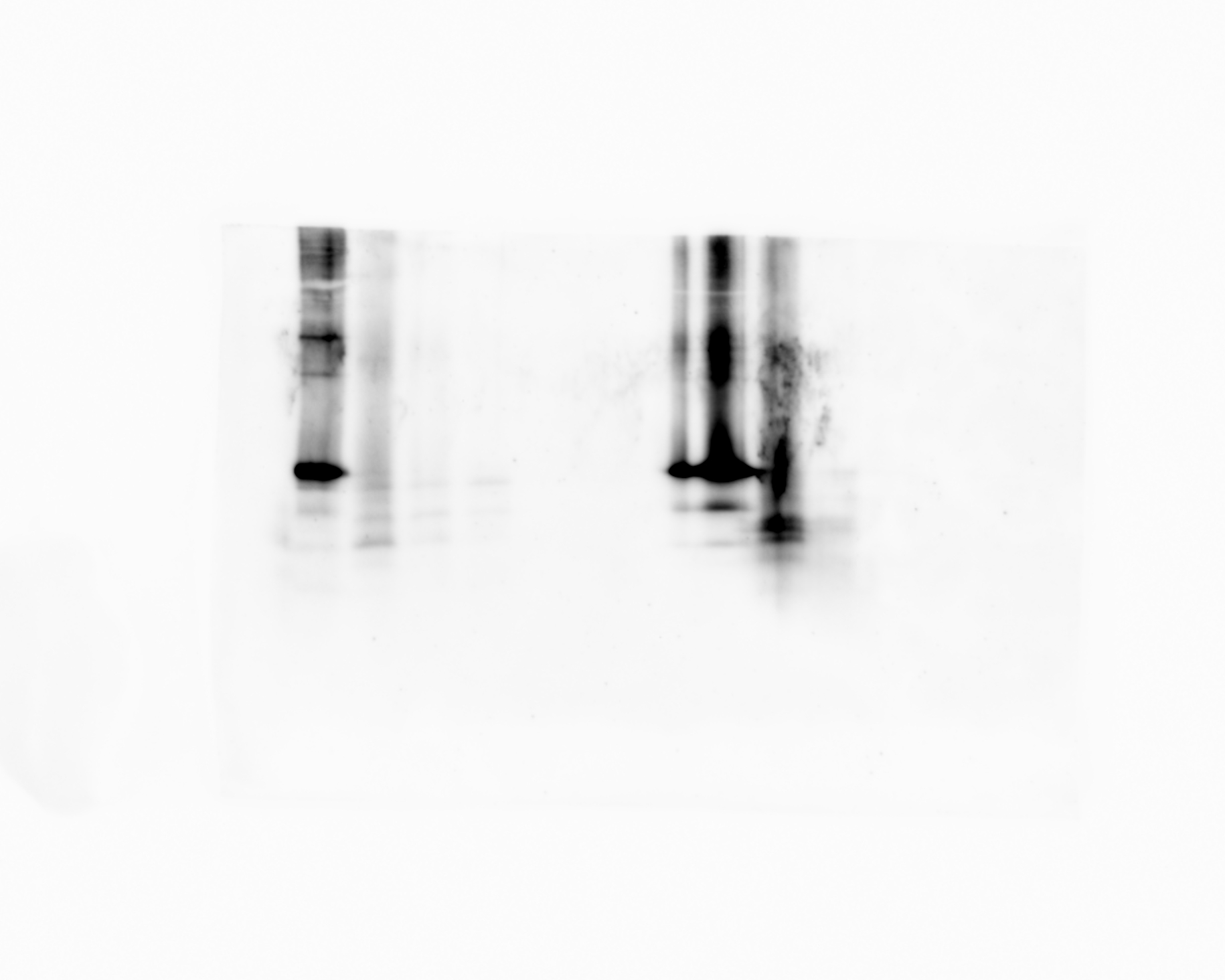

Supplement: Figure 3—figure supplement 1—source data 1. [file elife-92775-fig3-figsupp1-data1.zip › Figure 3 - figure supplement 1_Source data 1/Figure 3 - figure supplement 1 - C/C_DLB2_BD.jpg]

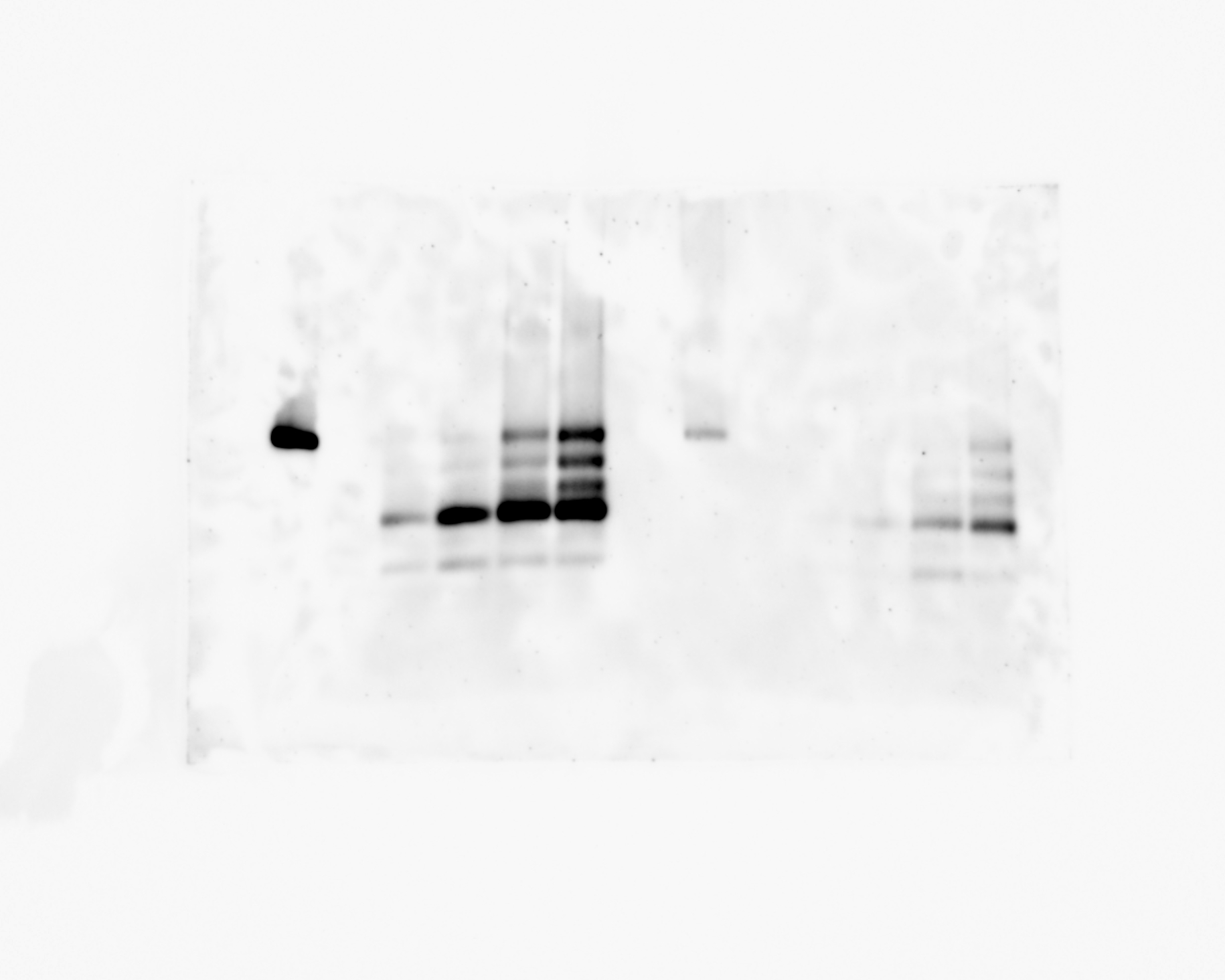

Supplement: Figure 3—figure supplement 1—source data 1. [file elife-92775-fig3-figsupp1-data1.zip › Figure 3 - figure supplement 1_Source data 1/Figure 3 - figure supplement 1 - C/C_DLB2_SAA.jpg]

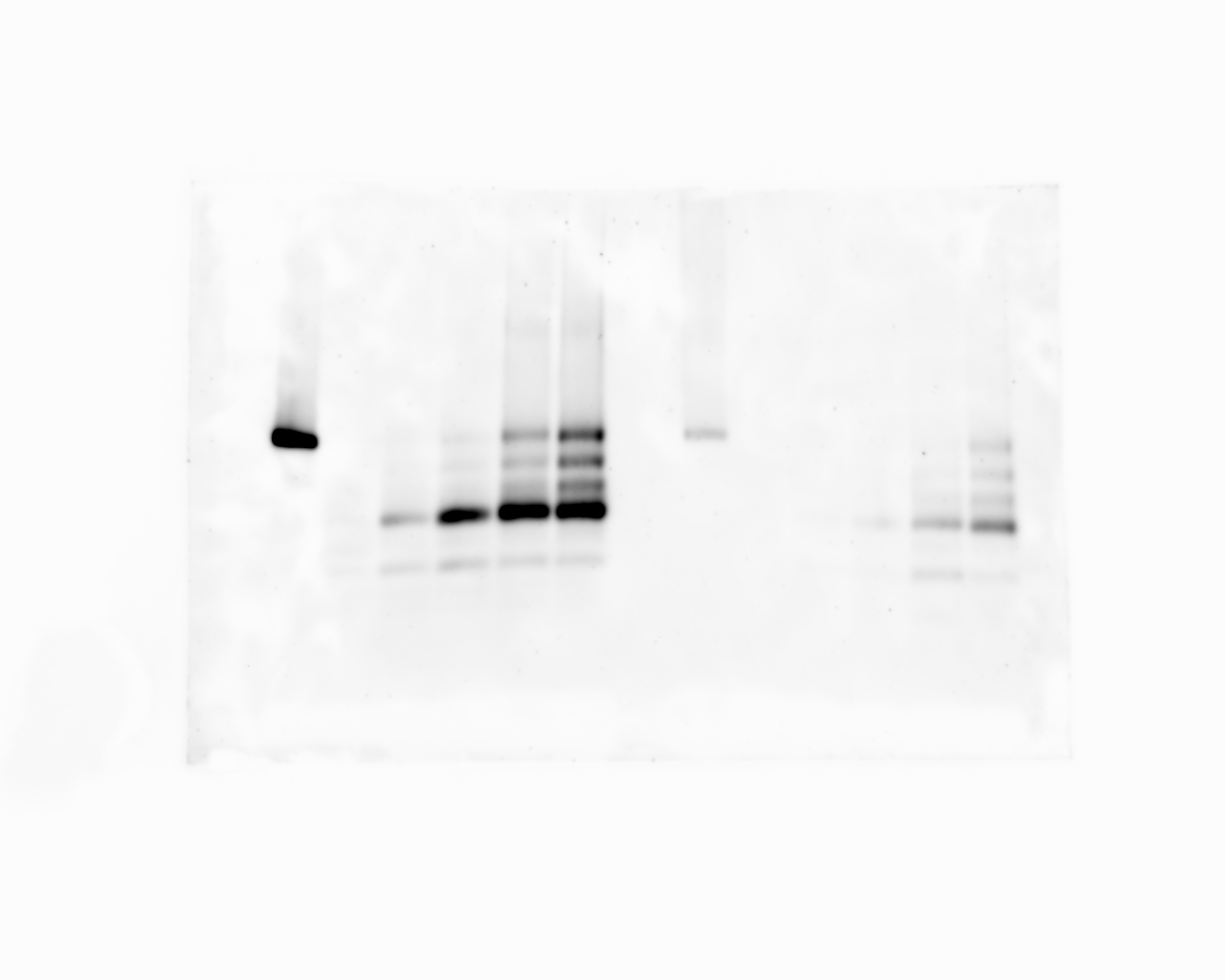

Supplement: Figure 3—figure supplement 1—source data 1. [file elife-92775-fig3-figsupp1-data1.zip › Figure 3 - figure supplement 1_Source data 1/Figure 3 - figure supplement 1 - C/C_DLB1_SAA.jpg]

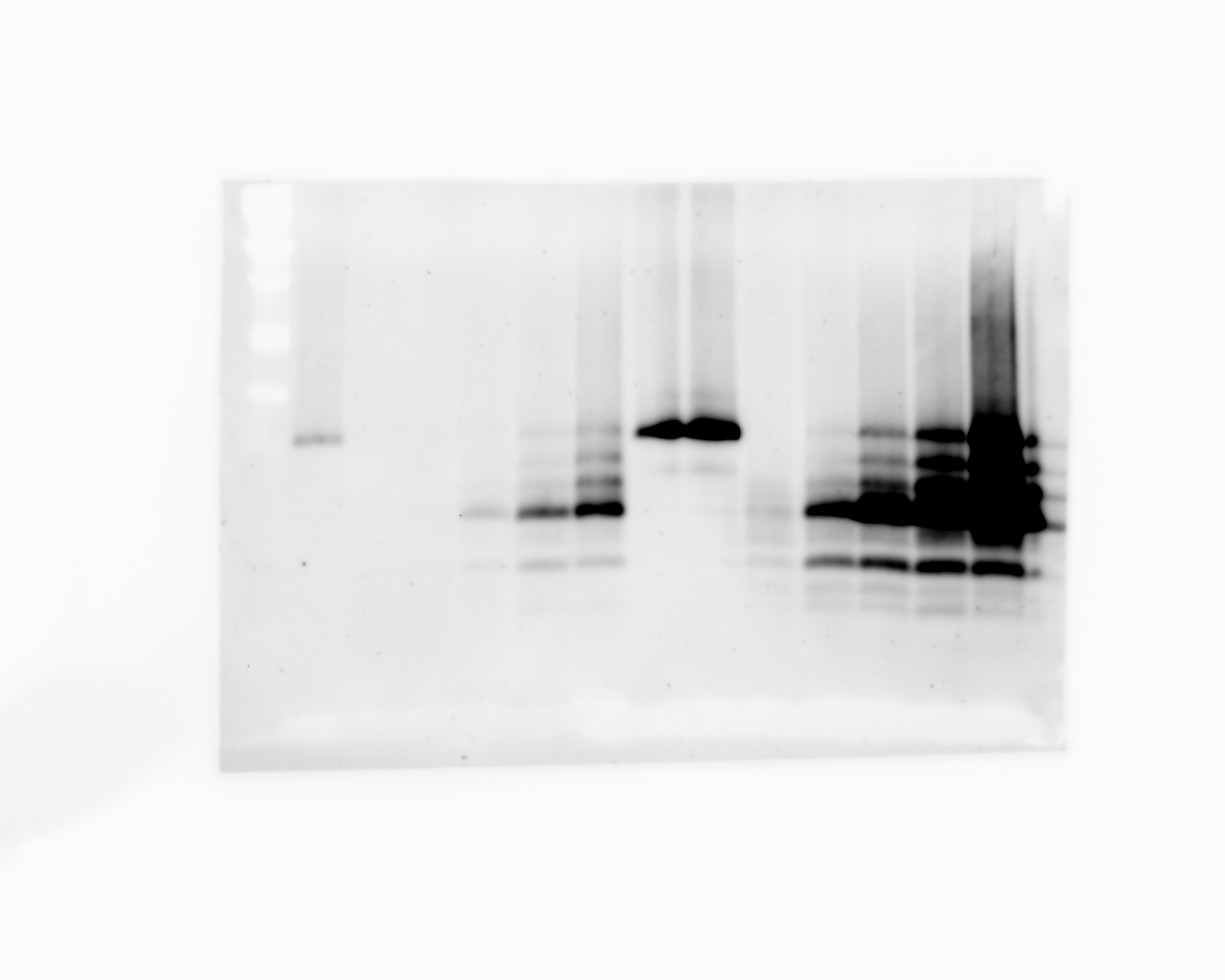

Supplement: Figure 3—figure supplement 1—source data 1. [file elife-92775-fig3-figsupp1-data1.zip › Figure 3 - figure supplement 1_Source data 1/Figure 3 - figure supplement 1 - B/B_PDD2_SAA.tif]

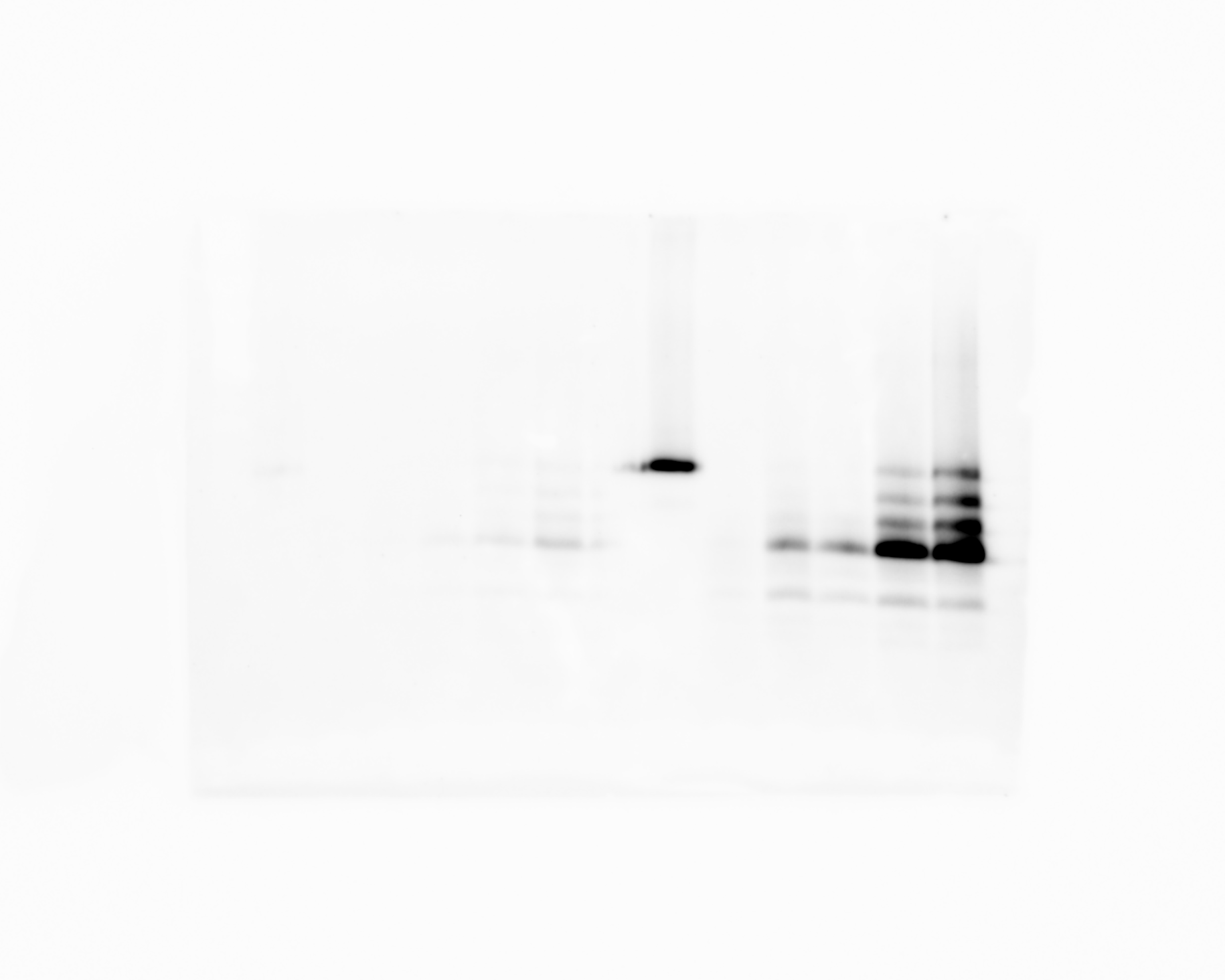

Supplement: Figure 3—figure supplement 1—source data 1. [file elife-92775-fig3-figsupp1-data1.zip › Figure 3 - figure supplement 1_Source data 1/Figure 3 - figure supplement 1 - B/B_PDD1_SAA.tif]

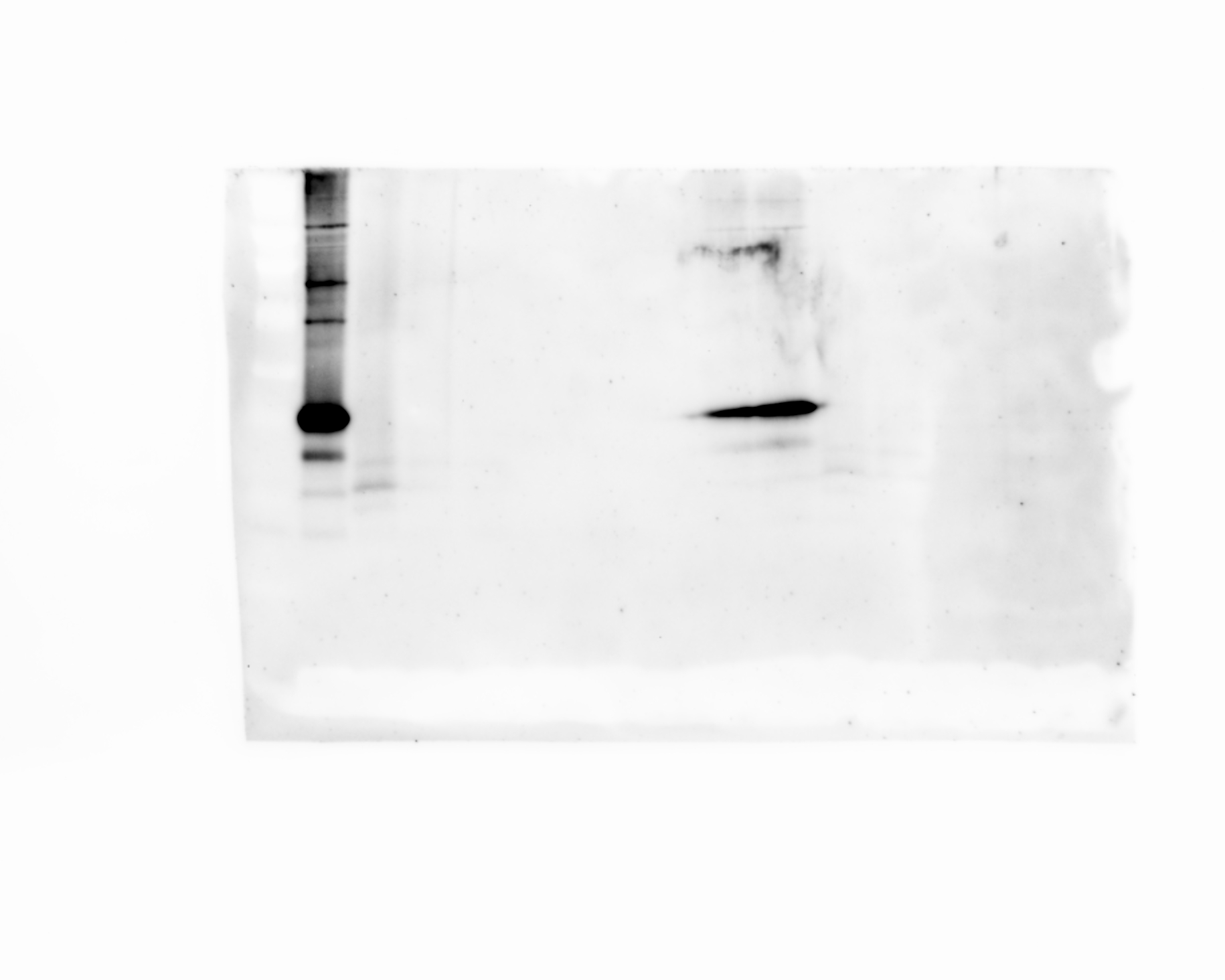

Supplement: Figure 3—figure supplement 1—source data 1. [file elife-92775-fig3-figsupp1-data1.zip › Figure 3 - figure supplement 1_Source data 1/Figure 3 - figure supplement 1 - B/B_PDD2_BD.jpg]

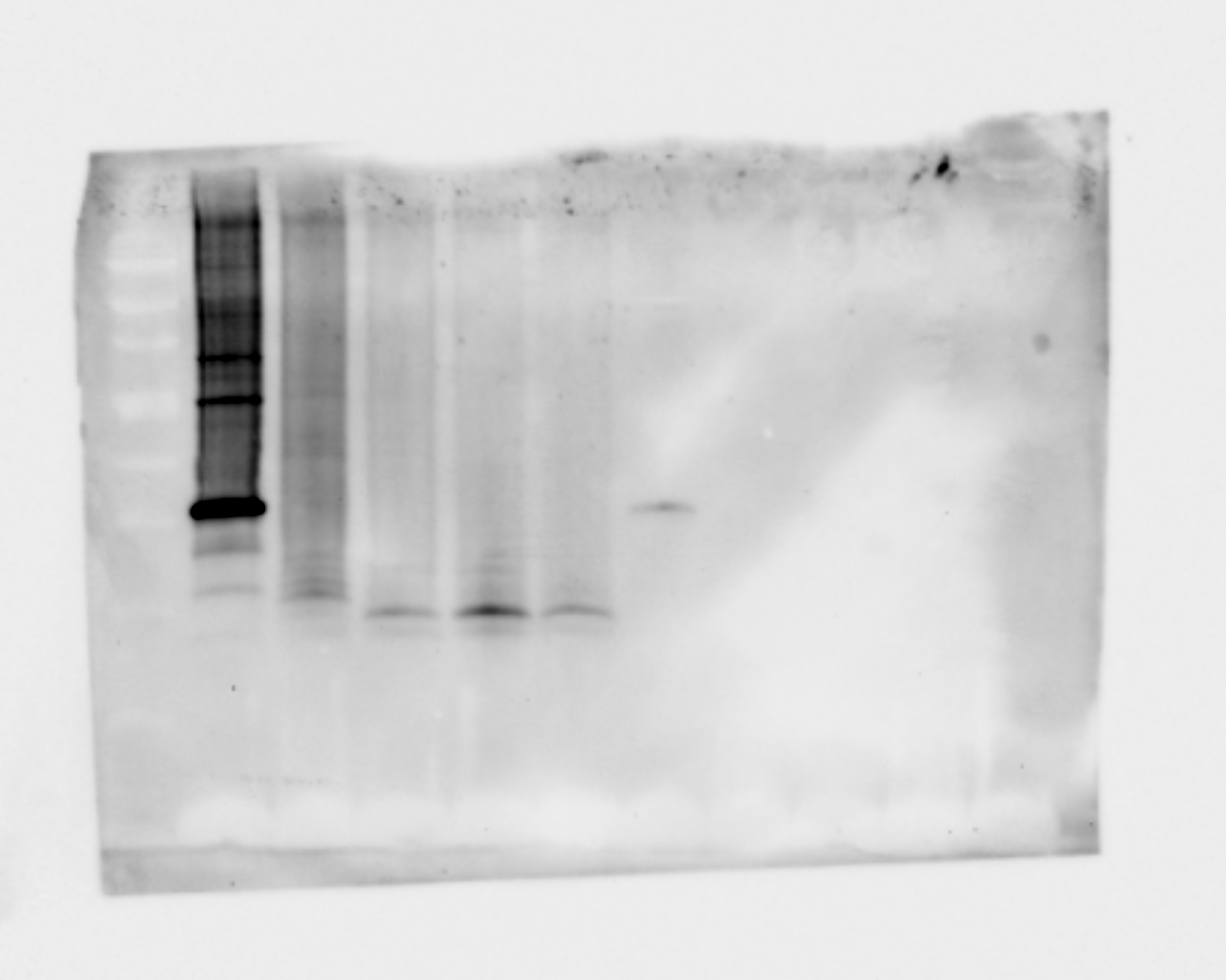

Supplement: Figure 4—source data 1. [file elife-92775-fig4-data1.zip › Figure 4_Source data 1/Fig.4_C/C_DLB_1.tif]

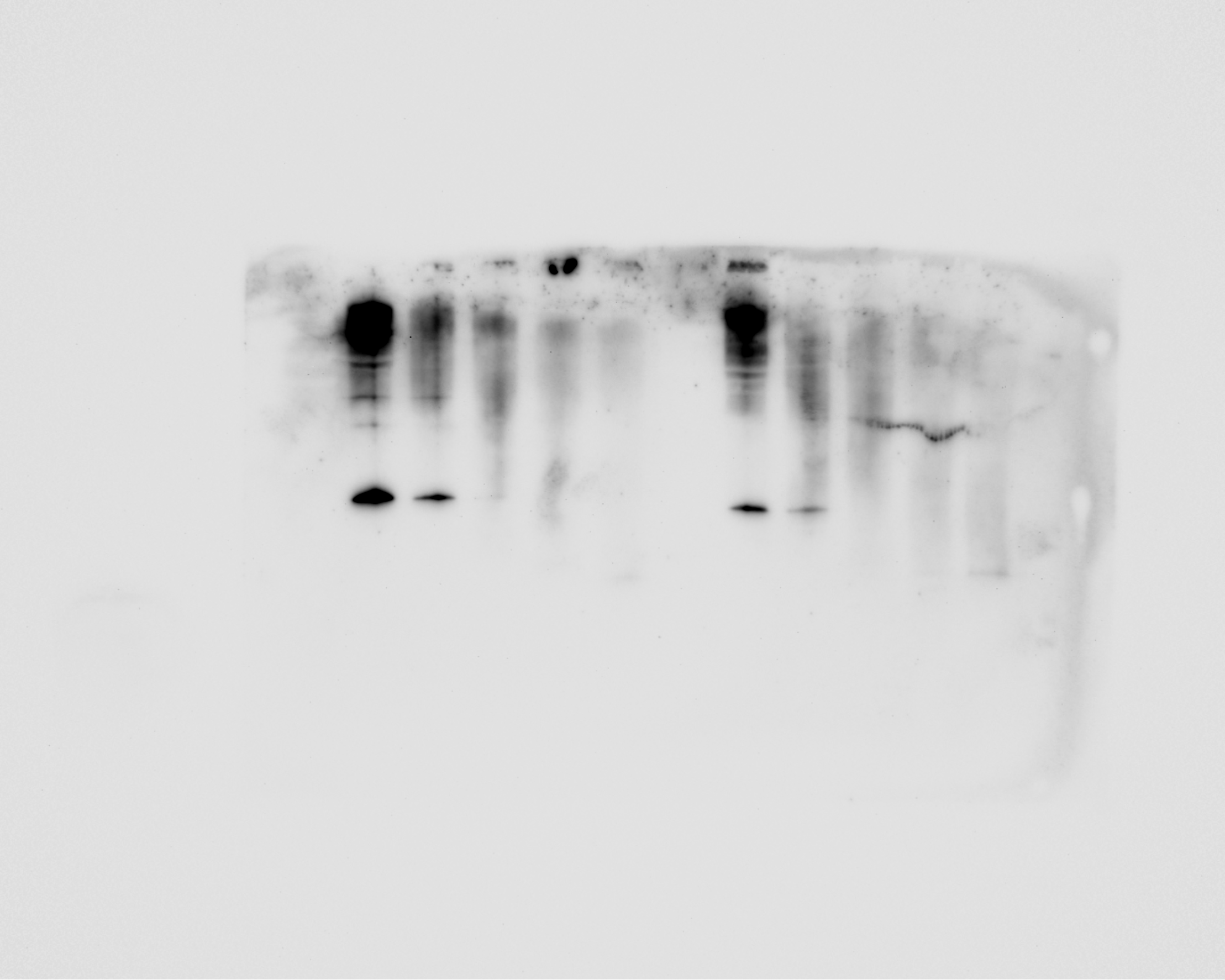

Supplement: Figure 4—source data 1. [file elife-92775-fig4-data1.zip › Figure 4_Source data 1/Fig.4_C/C_DLB_3.tif]

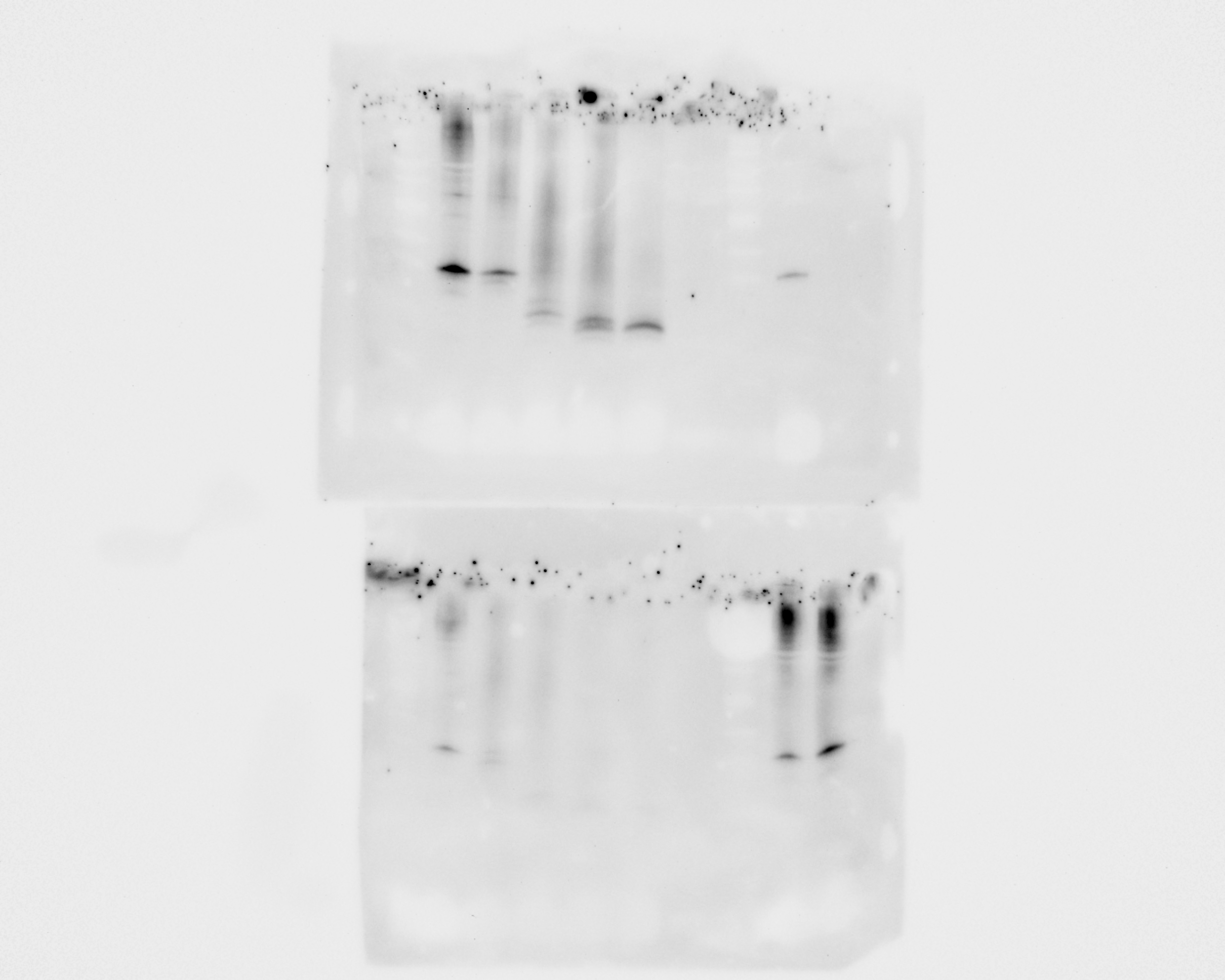

Supplement: Figure 4—source data 1. [file elife-92775-fig4-data1.zip › Figure 4_Source data 1/Fig.4_C/C_DLB_2.tif]

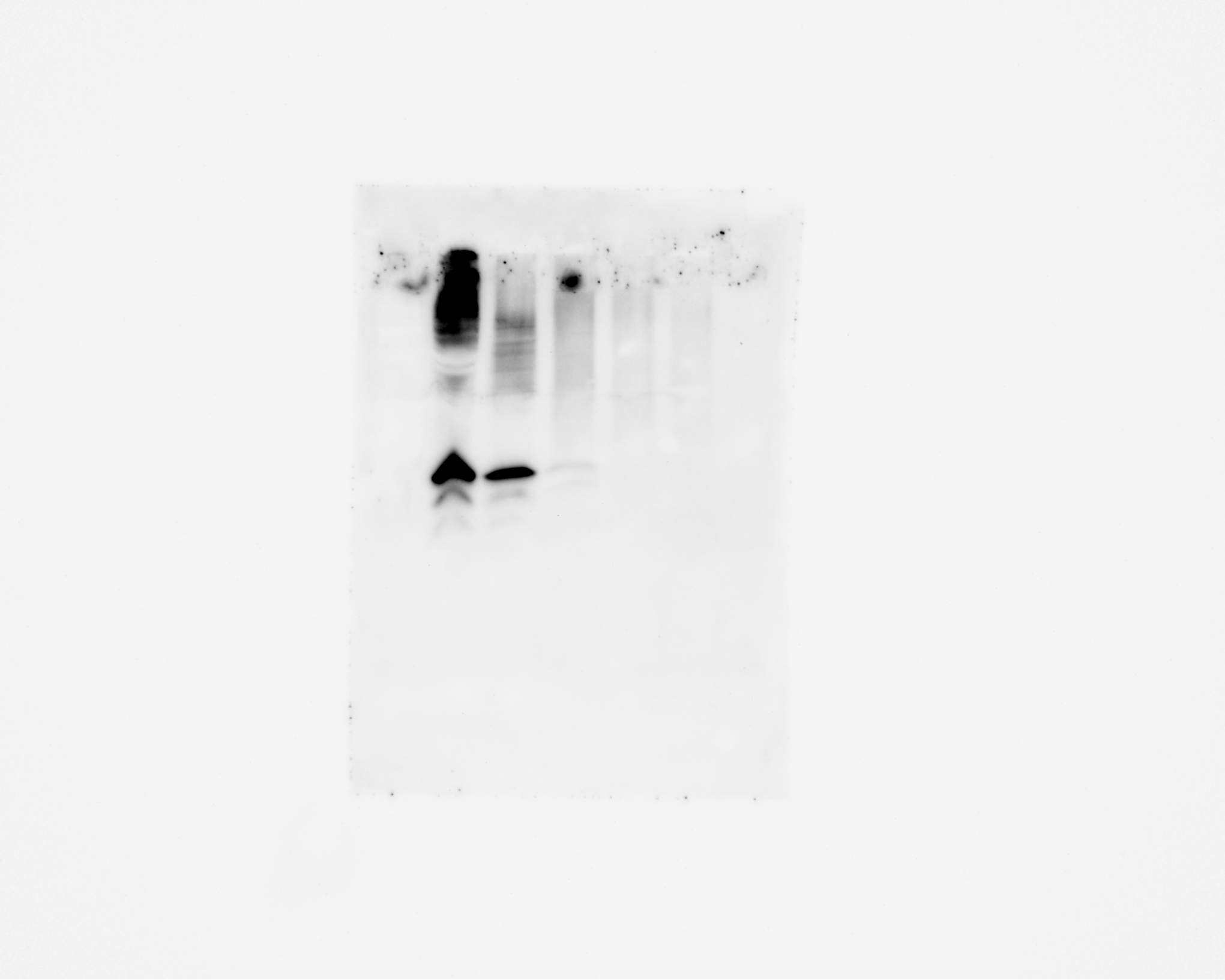

Supplement: Figure 4—source data 1. [file elife-92775-fig4-data1.zip › Figure 4_Source data 1/Fig.4_D/D_MSA_2.tif]

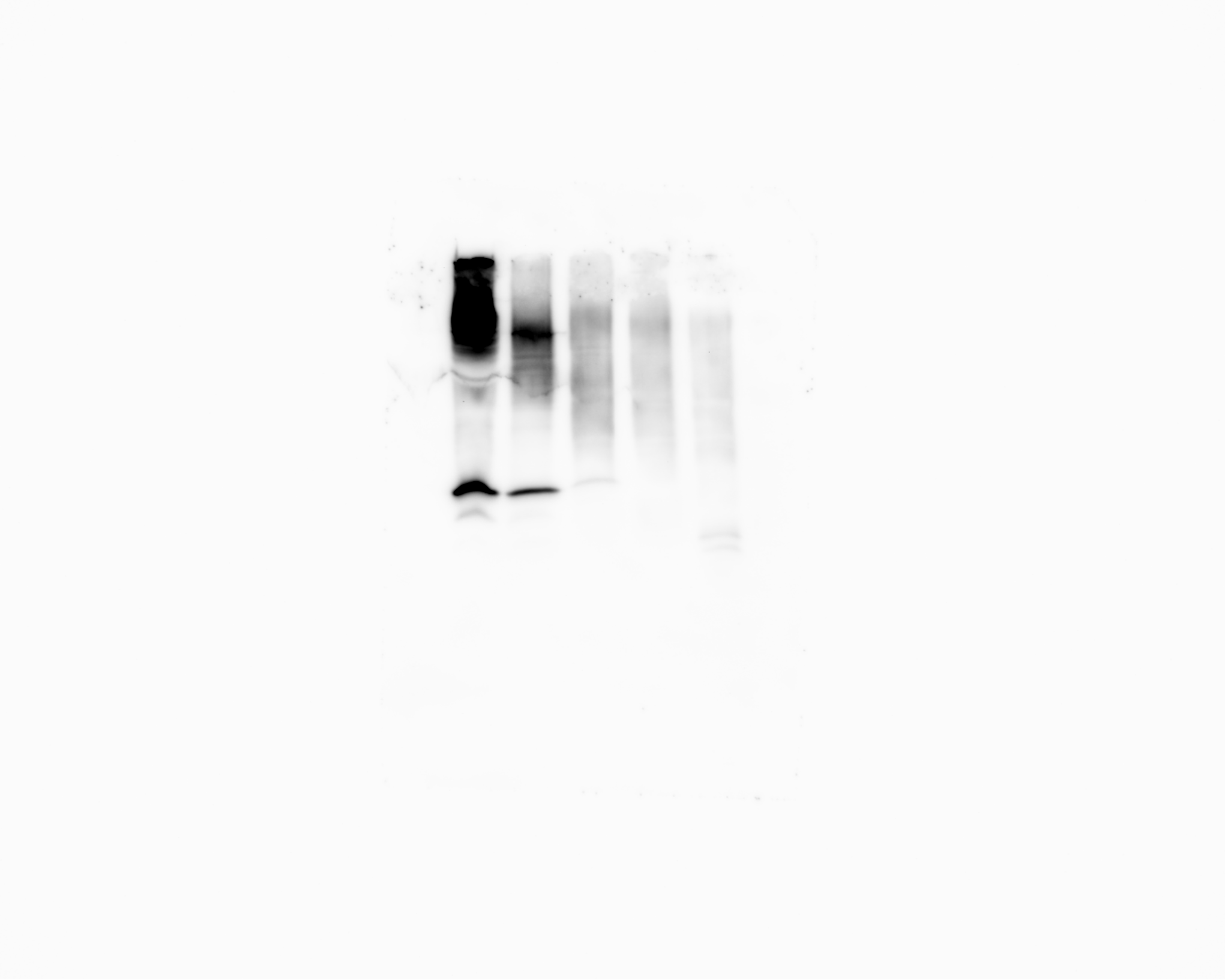

Supplement: Figure 4—source data 1. [file elife-92775-fig4-data1.zip › Figure 4_Source data 1/Fig.4_D/D_MSA_3.tif]

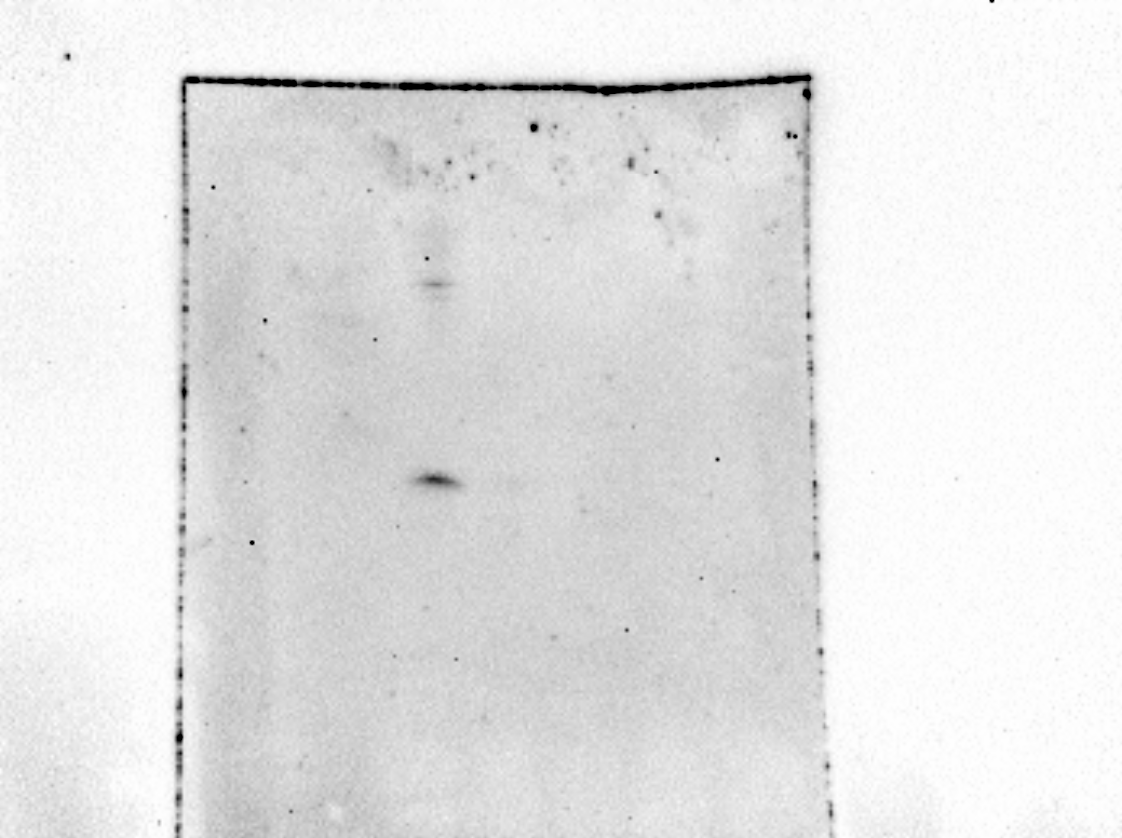

Supplement: Figure 4—source data 1. [file elife-92775-fig4-data1.zip › Figure 4_Source data 1/Fig.4_E/hc1_PK_120sec.tif]

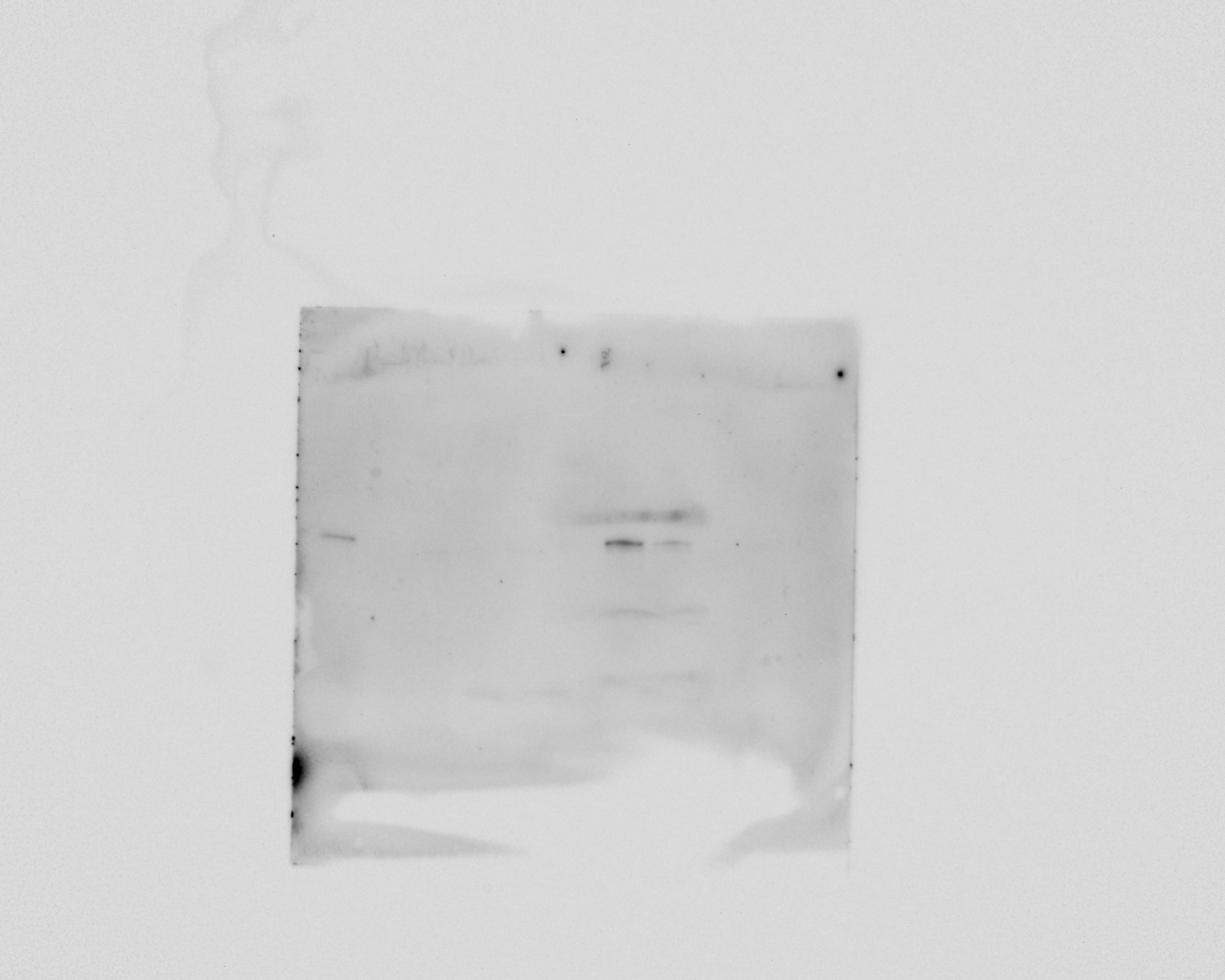

Supplement: Figure 4—source data 1. [file elife-92775-fig4-data1.zip › Figure 4_Source data 1/Fig.4_E/E_HC_2.tif]

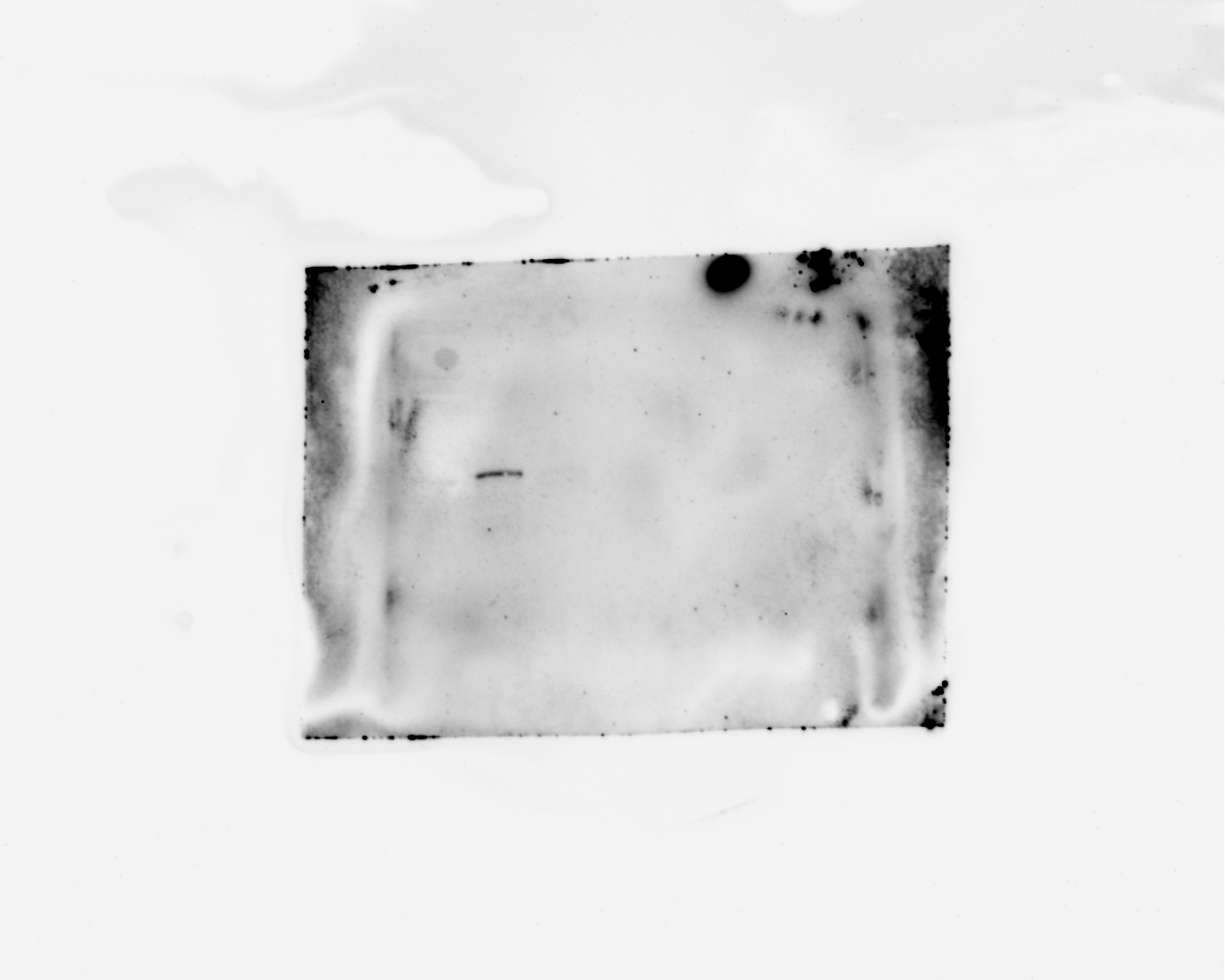

Supplement: Figure 4—source data 1. [file elife-92775-fig4-data1.zip › Figure 4_Source data 1/Fig.4_E/E_HC_3.tif]

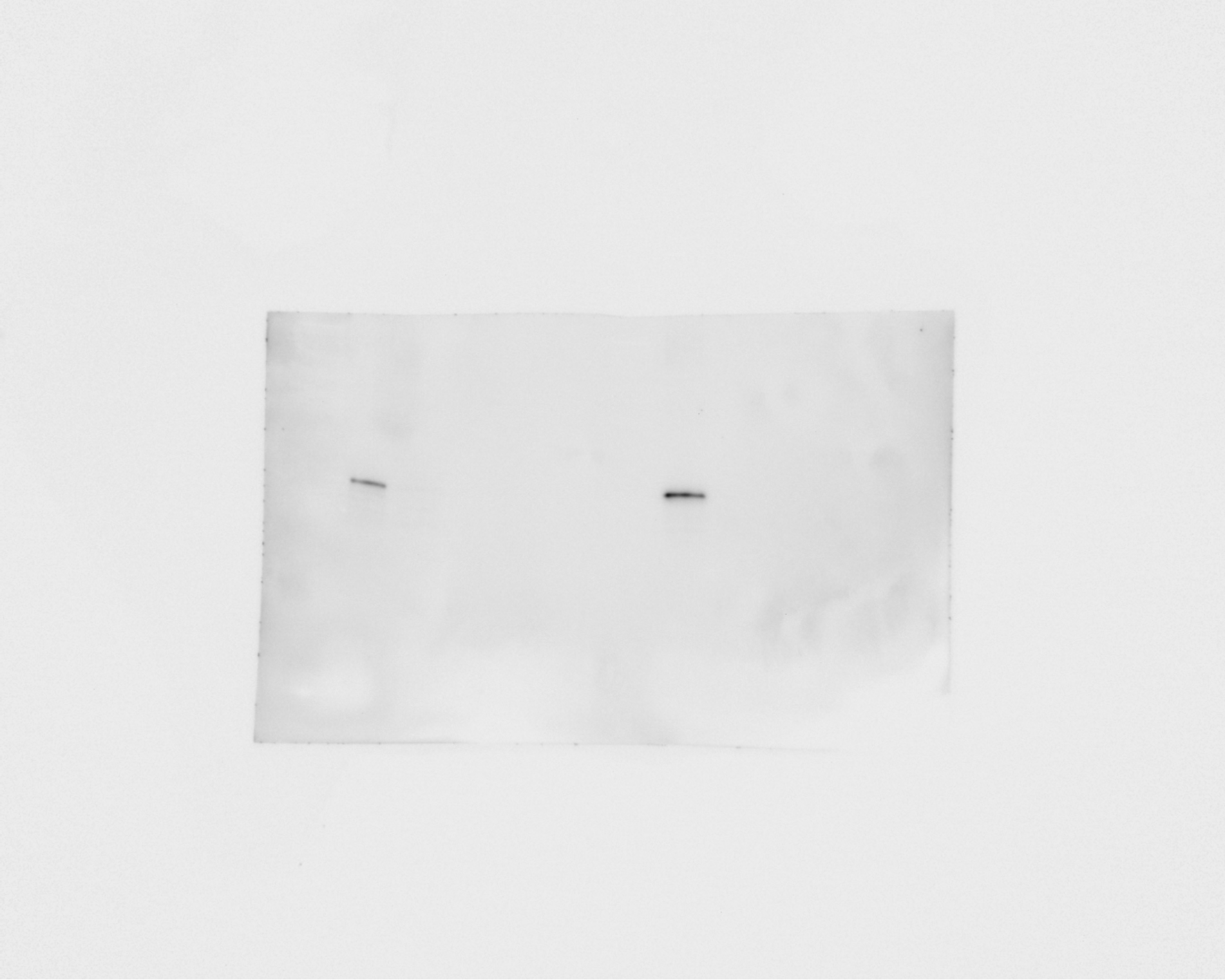

Supplement: Figure 4—source data 1. [file elife-92775-fig4-data1.zip › Figure 4_Source data 1/Fig.4_E/Selene 2024-04-23 07h11m18s(Chemiluminescence).tif]

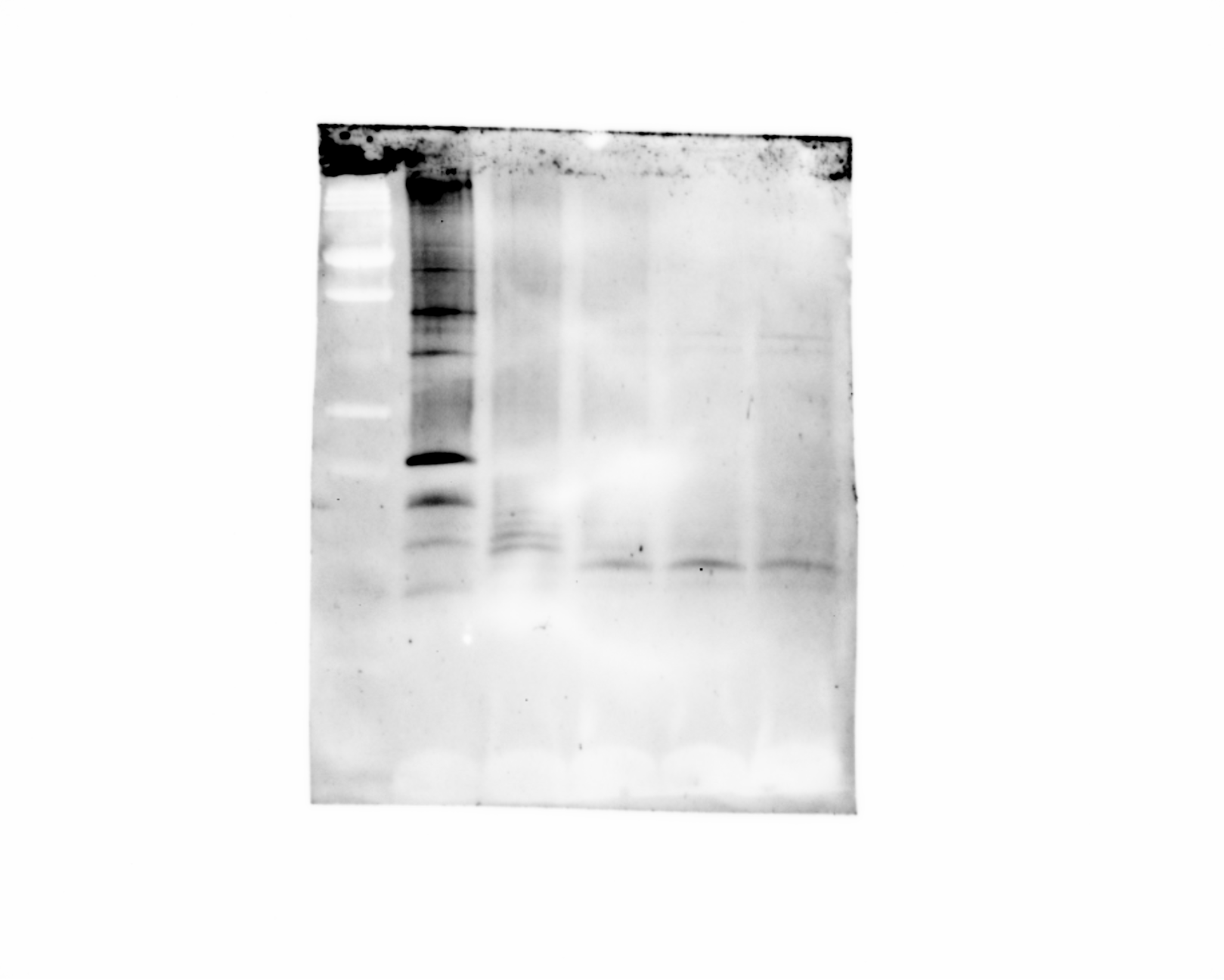

Supplement: Figure 4—source data 1. [file elife-92775-fig4-data1.zip › Figure 4_Source data 1/Fig.4_B/B_PDD_2.tif]

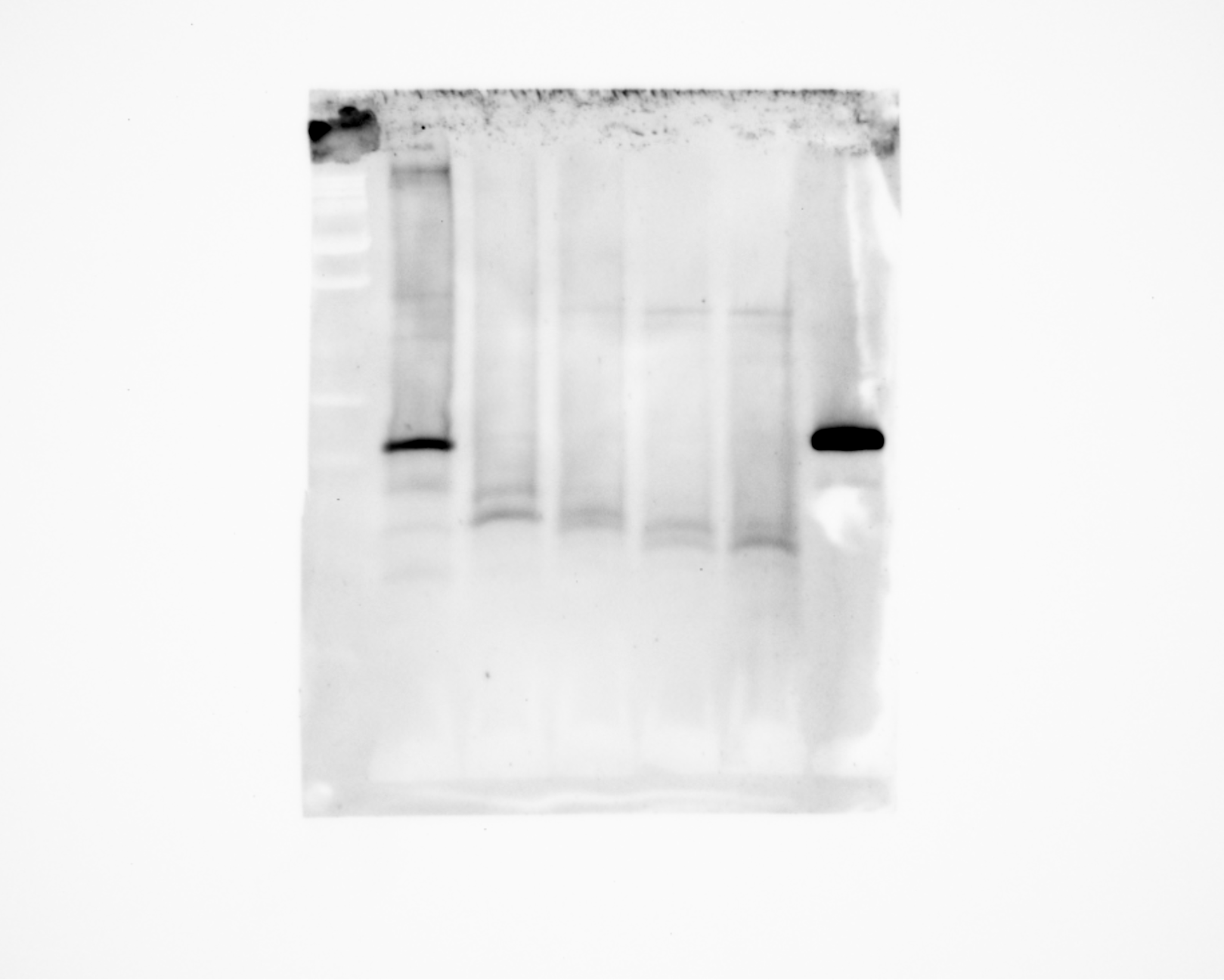

Supplement: Figure 4—source data 1. [file elife-92775-fig4-data1.zip › Figure 4_Source data 1/Fig.4_B/B_PDD_3.tif]

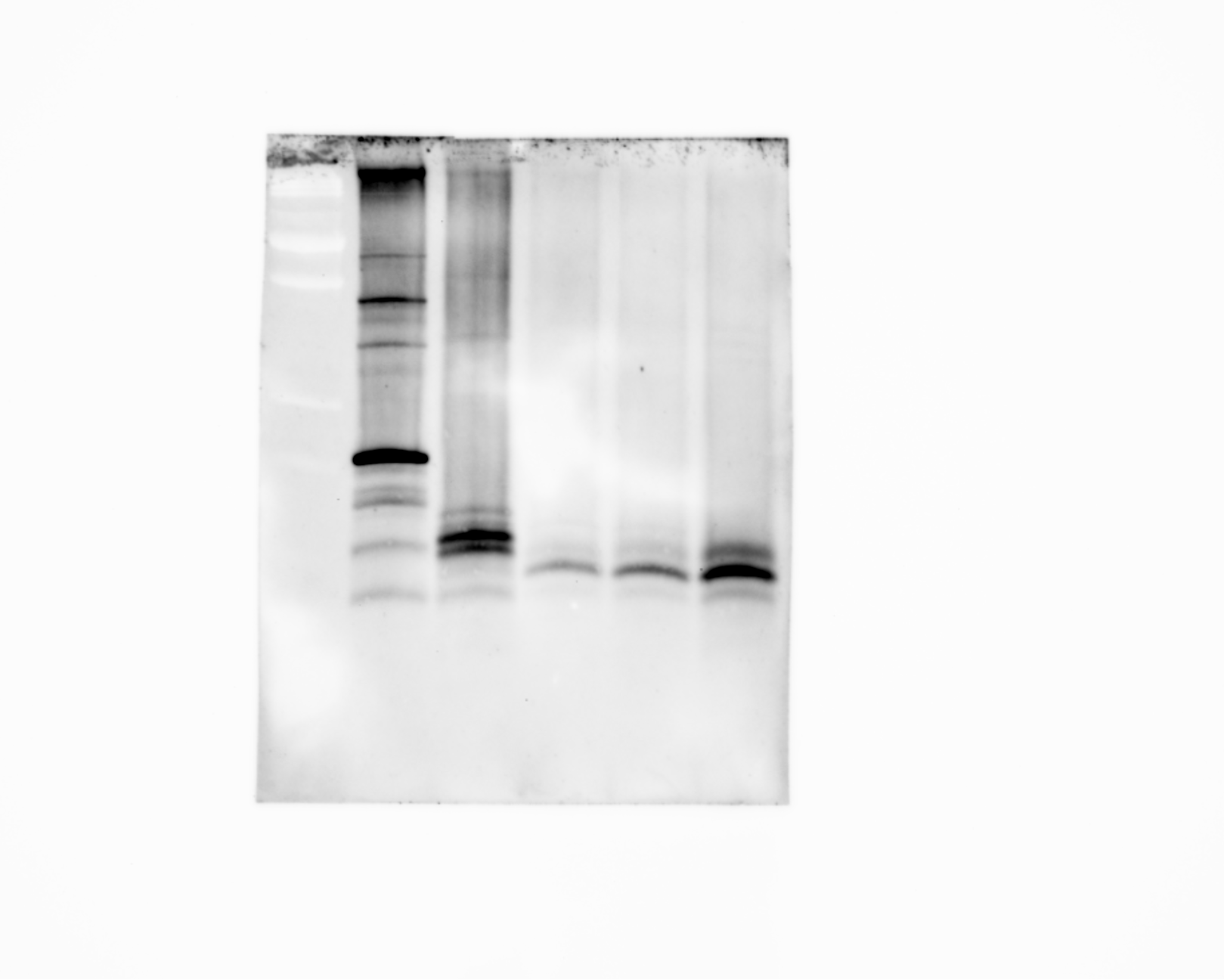

Supplement: Figure 4—source data 1. [file elife-92775-fig4-data1.zip › Figure 4_Source data 1/Fig.4_B/B_PDD_1.tif]

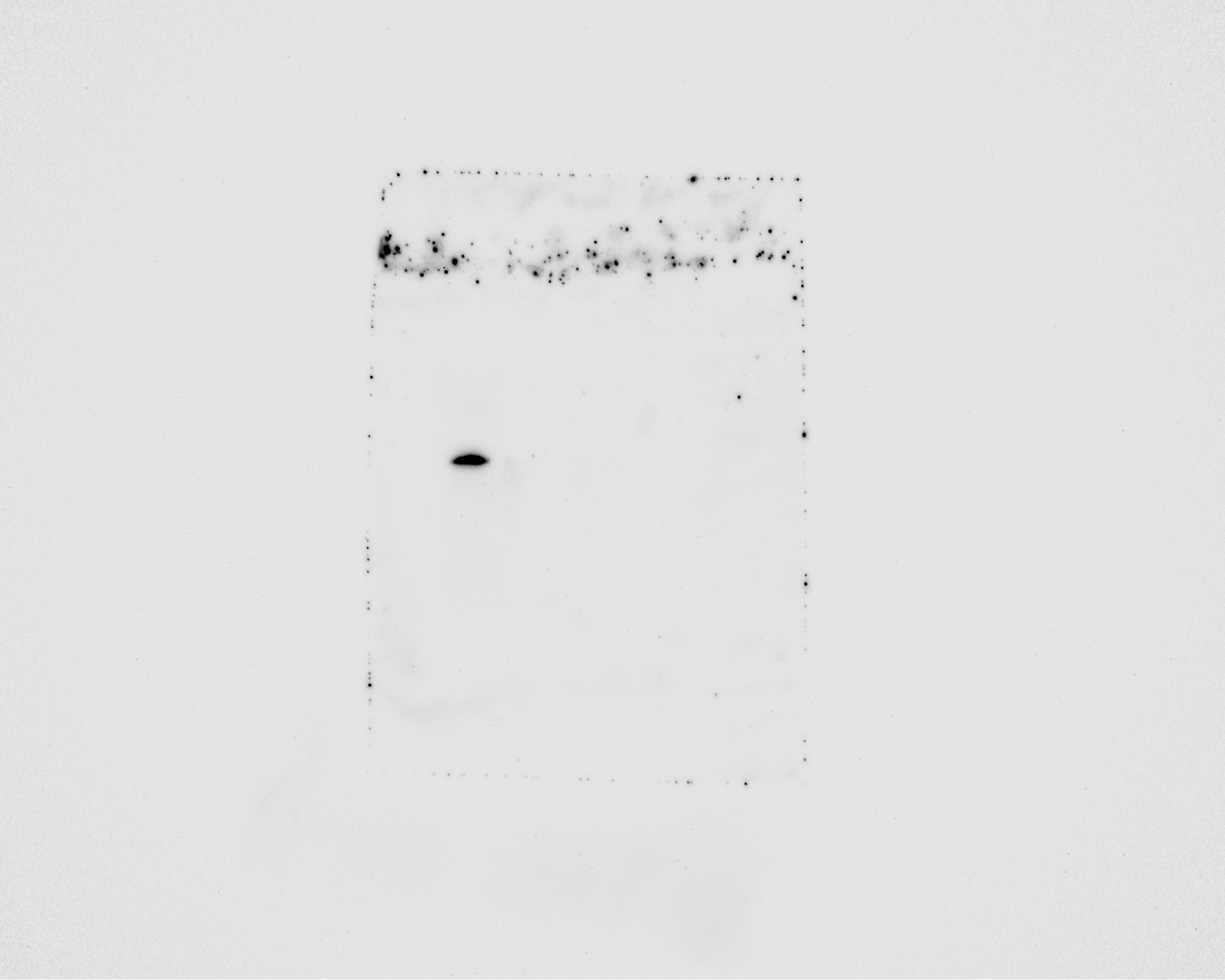

Supplement: Figure 4—source data 1. [file elife-92775-fig4-data1.zip › Figure 4_Source data 1/Fig.4_A/A_PD_3.tif]

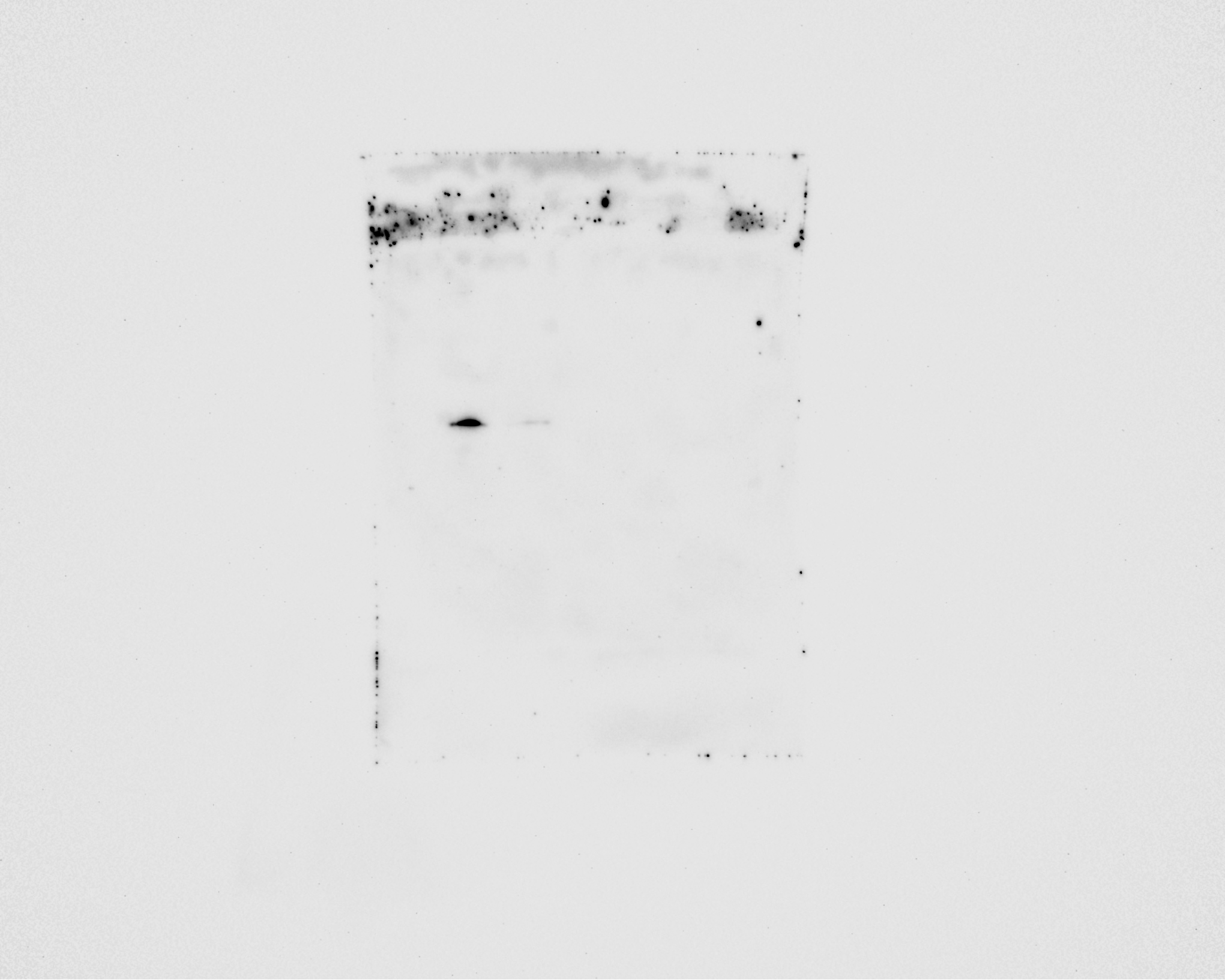

Supplement: Figure 4—source data 1. [file elife-92775-fig4-data1.zip › Figure 4_Source data 1/Fig.4_A/A_PD_2.tif]

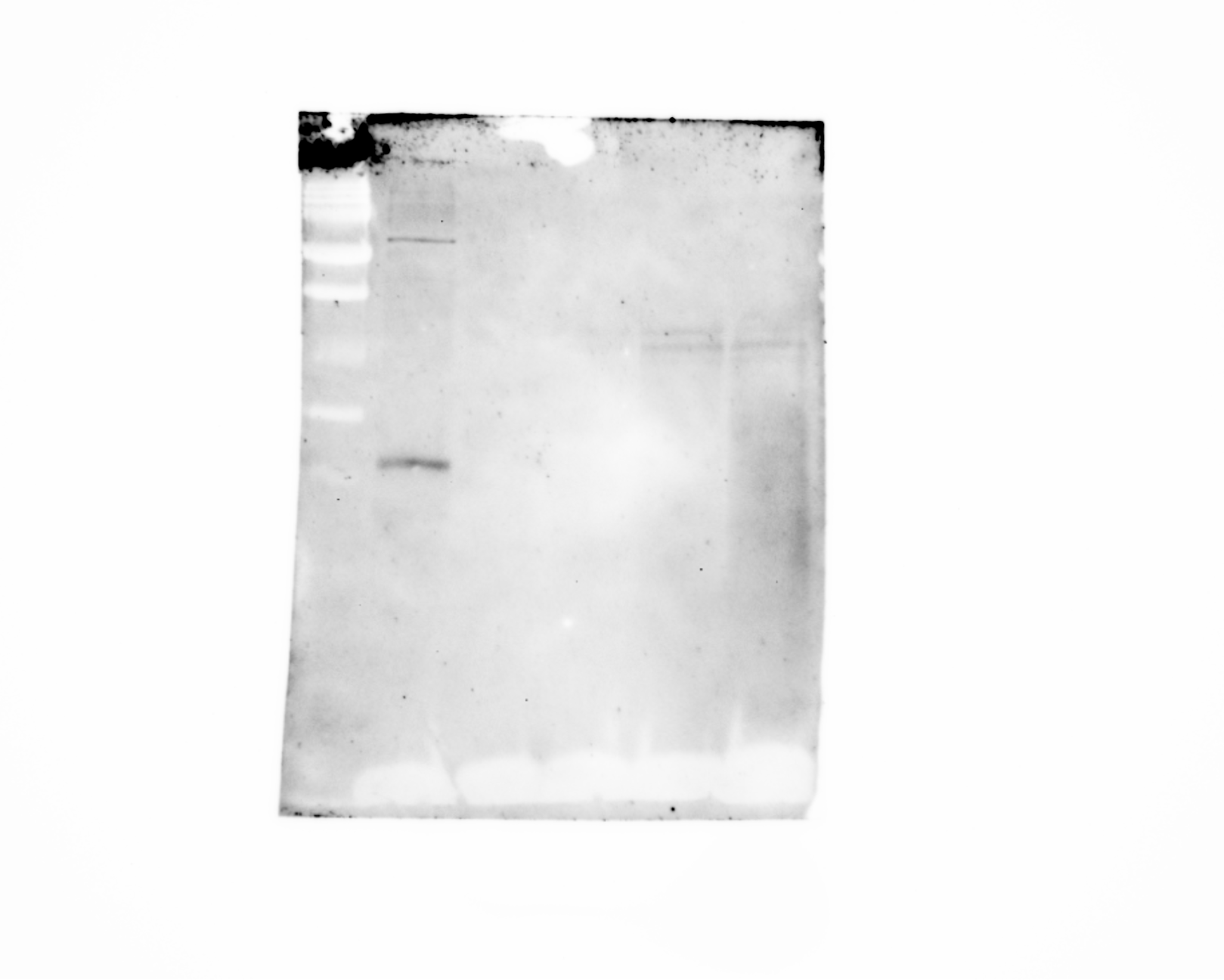

Supplement: Figure 4—source data 1. [file elife-92775-fig4-data1.zip › Figure 4_Source data 1/Fig.4_A/A_PD_1.tif]

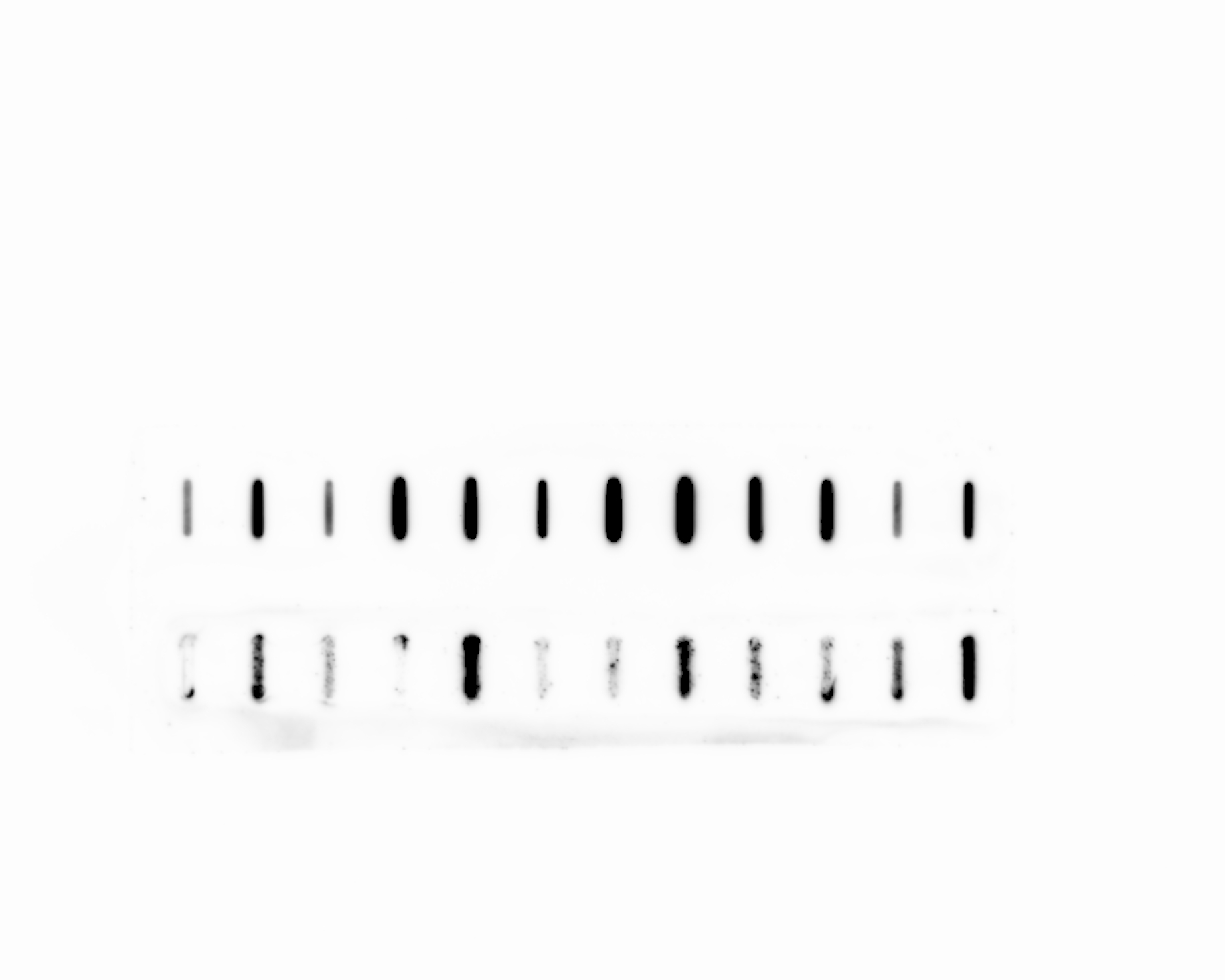

Supplement: Figure 6—source data 1. [file elife-92775-fig6-data1.zip › Figure 6_Source data 1/Brain_derived_MJFR-14.jpg]

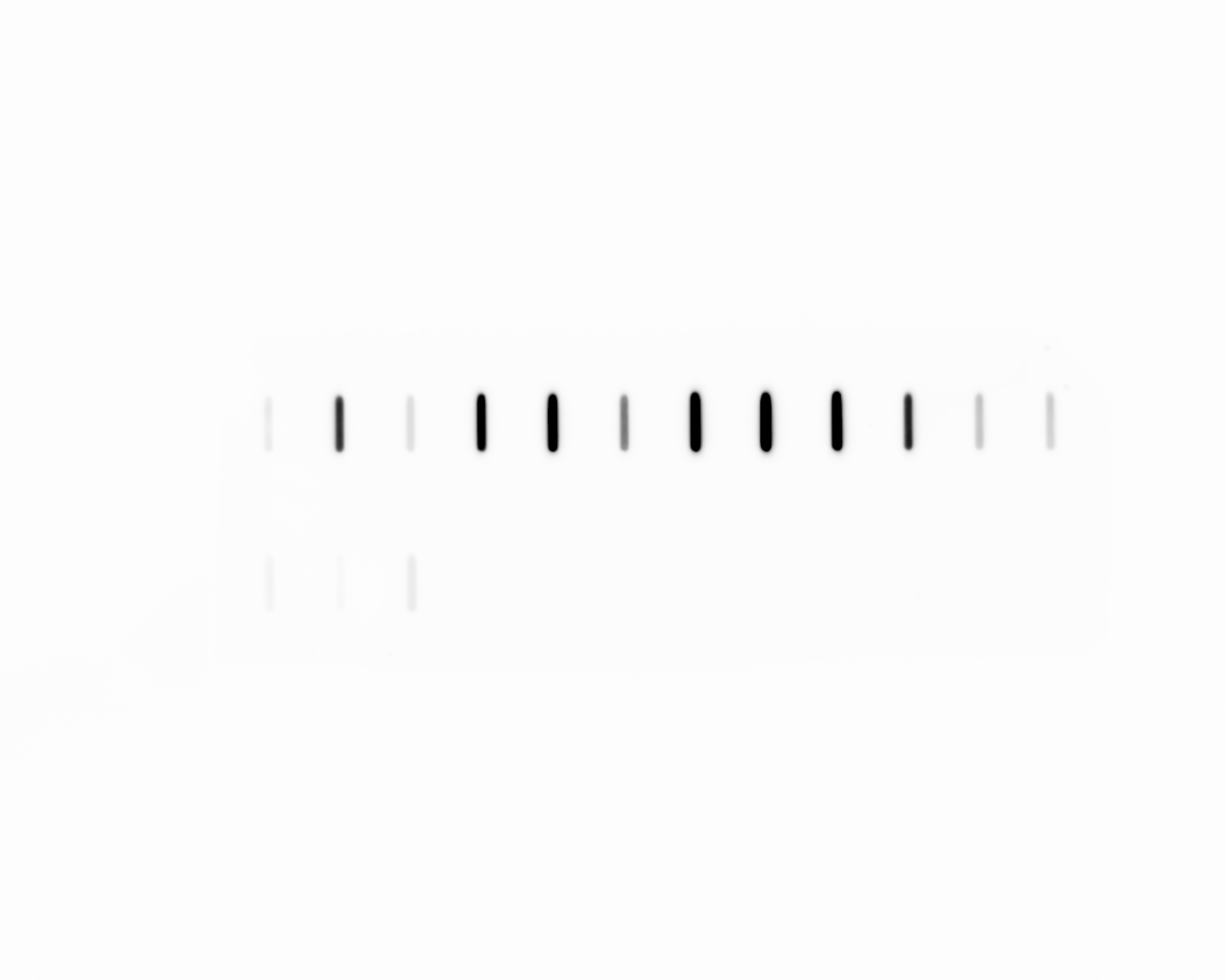

Supplement: Figure 6—source data 1. [file elife-92775-fig6-data1.zip › Figure 6_Source data 1/Brain_derived_pS129.jpg]

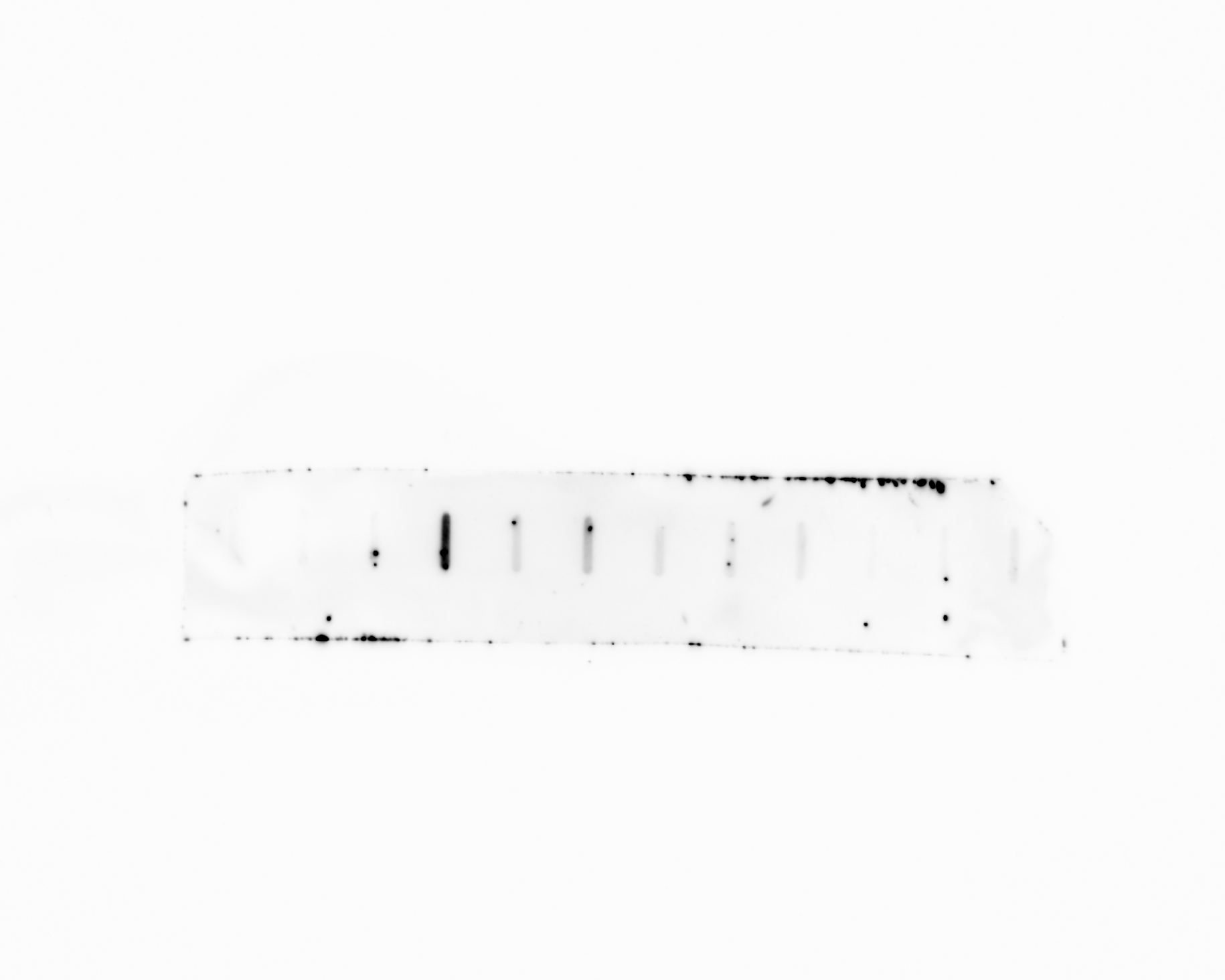

Supplement: Figure 6—source data 1. [file elife-92775-fig6-data1.zip › Figure 6_Source data 1/SAA_pS129.jpg]

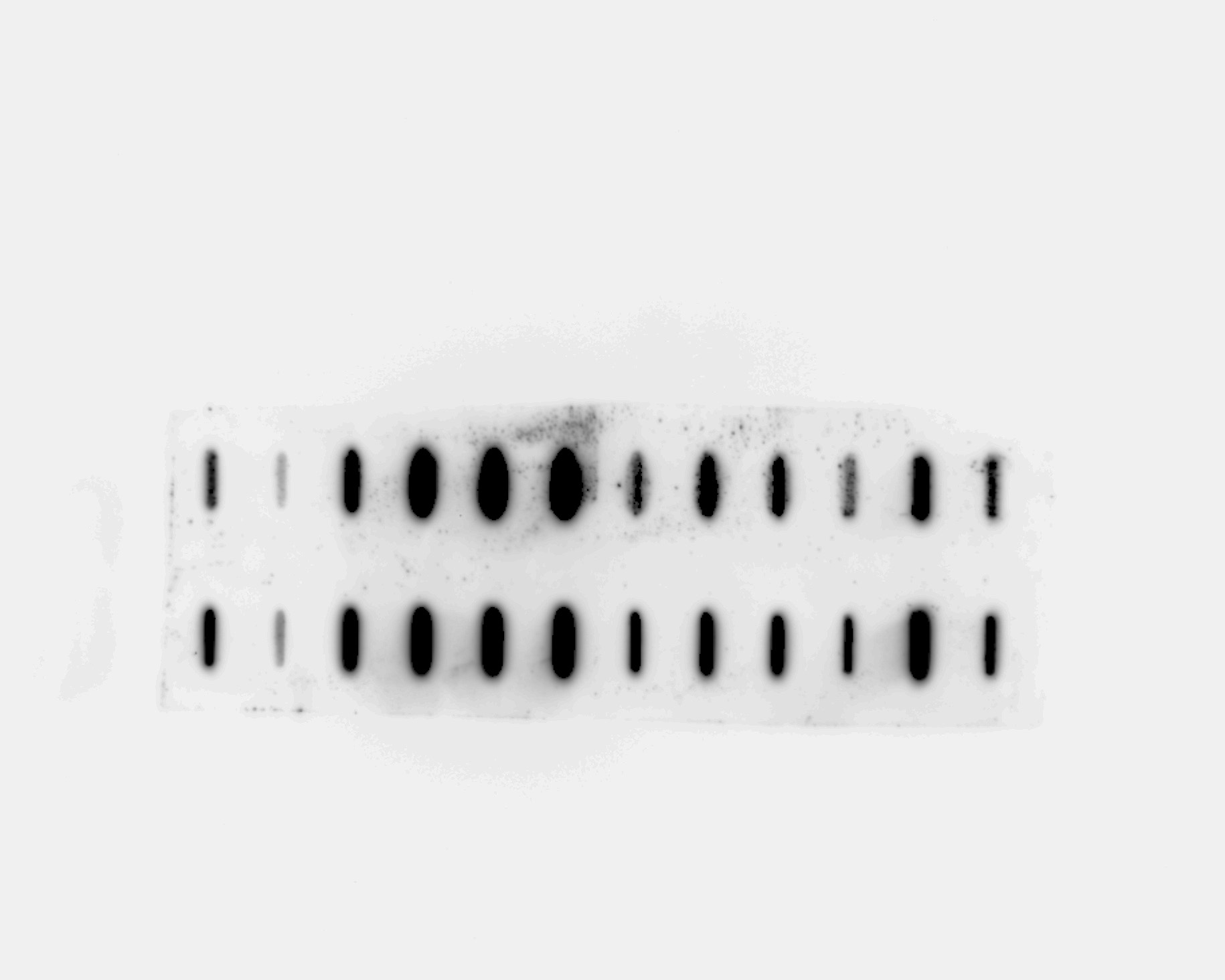

Supplement: Figure 6—source data 1. [file elife-92775-fig6-data1.zip › Figure 6_Source data 1/SAA_MJFR-14.jpg]

Figure 6 - C slot blots

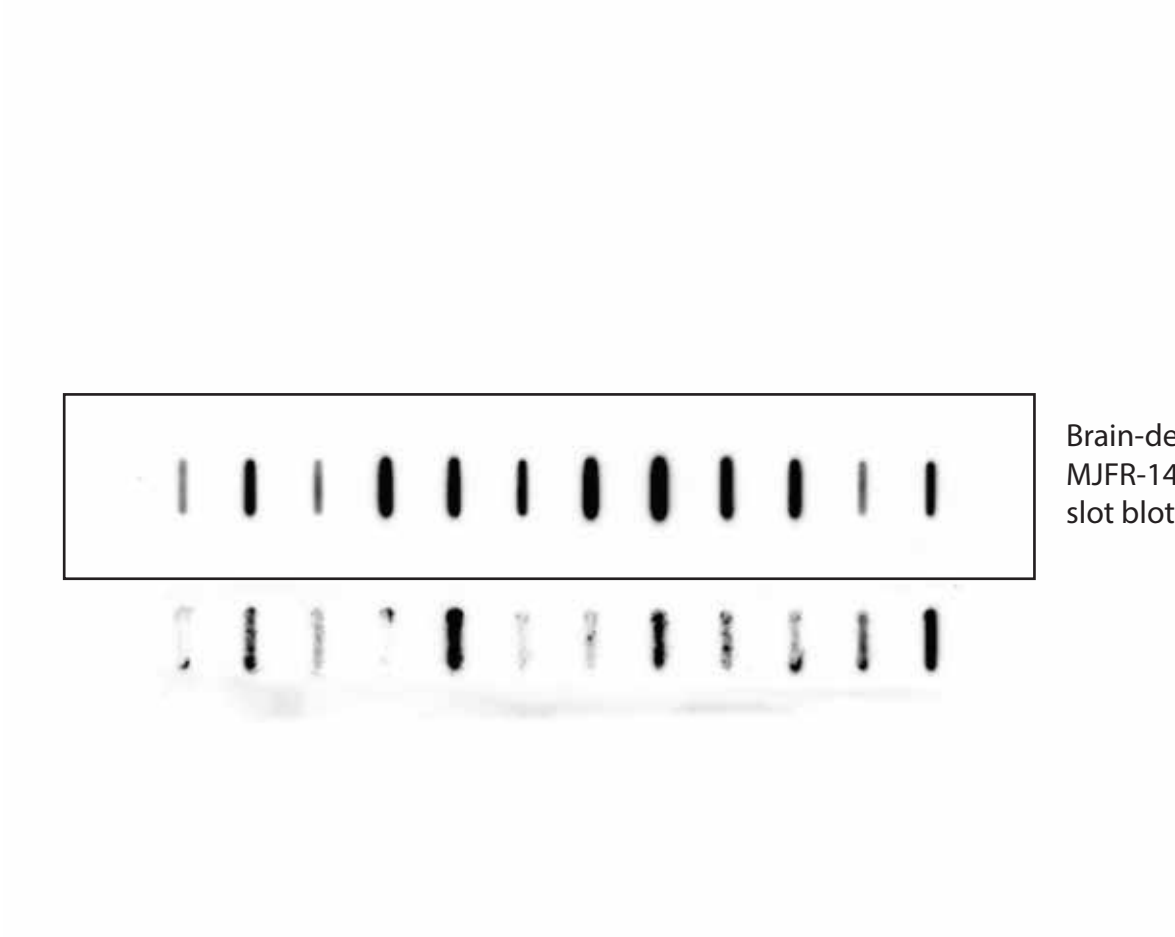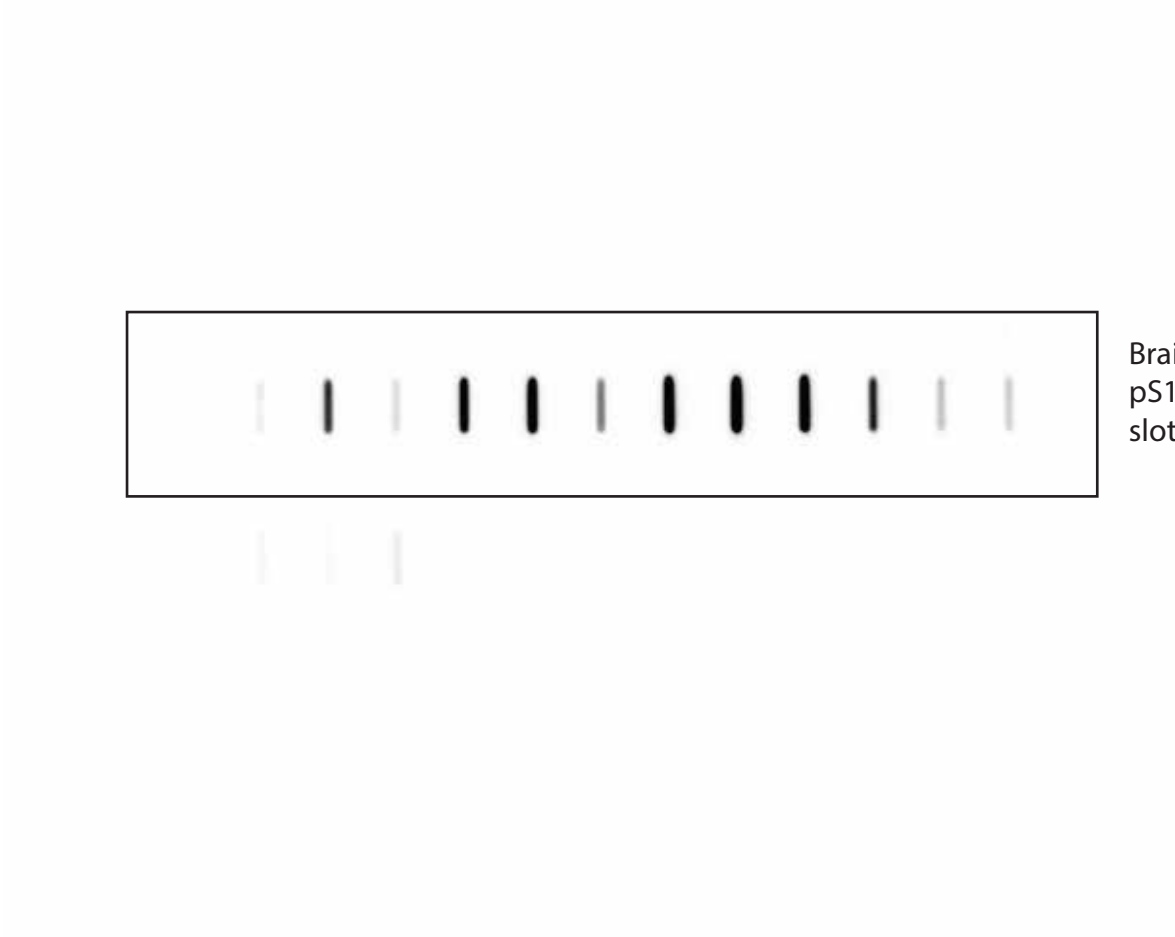

Figure 6 - D slot blots

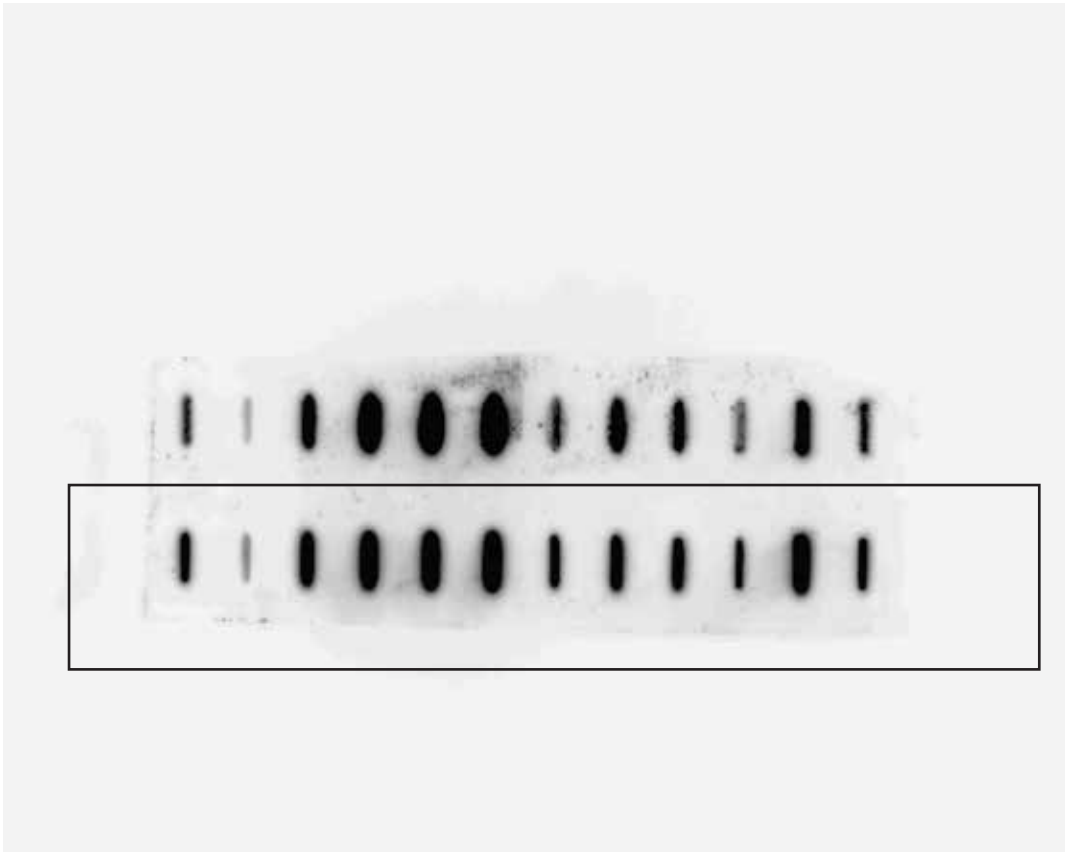

SAA fibrils  
MJFR-14  
slot blot

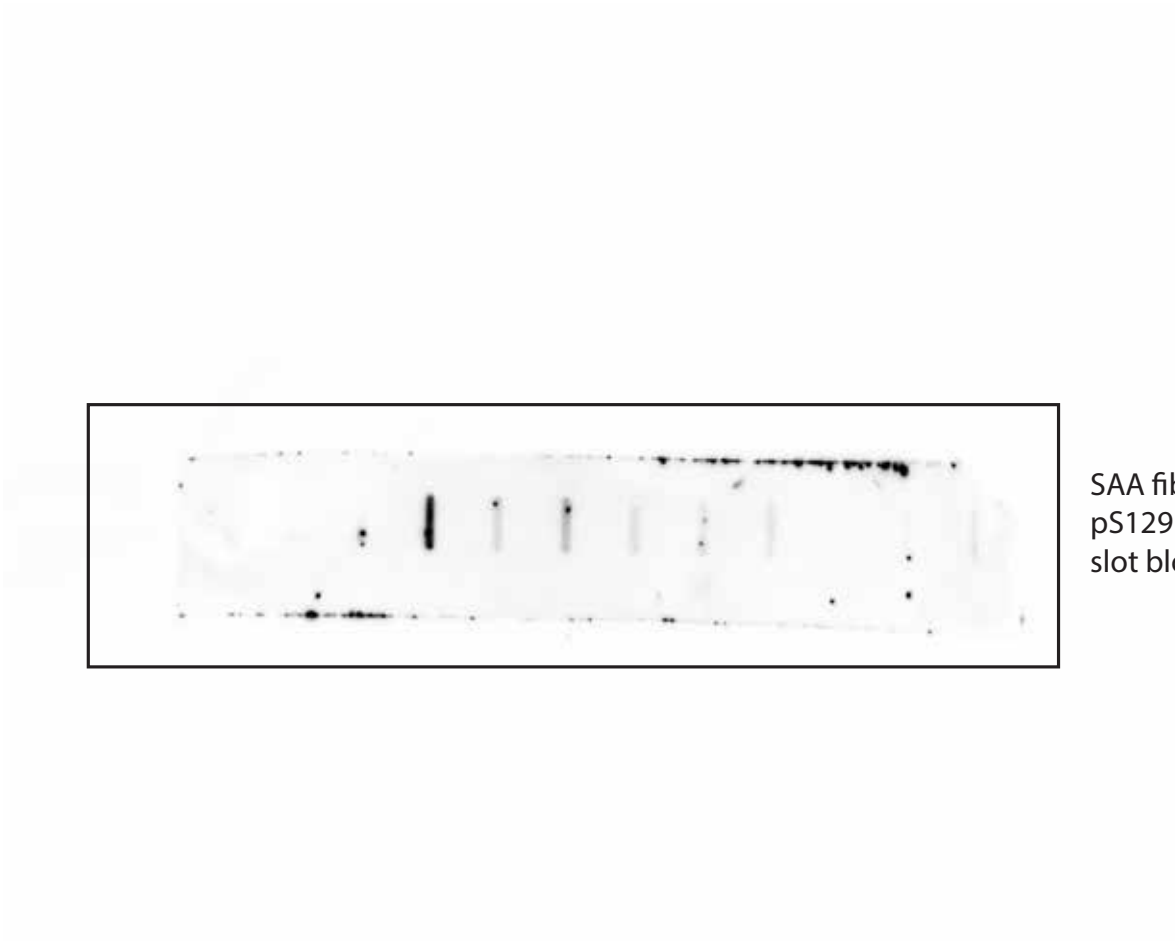

SAA fibrils  
pS129  
slot blot

Supplement: Figure 6—source data 2. [file elife-92775-fig6-data2.zip › Figure 6_Source data 2/Figure 6_Source data 2.pdf]

Figure 6 - figure supplement 1 A-D slot blots

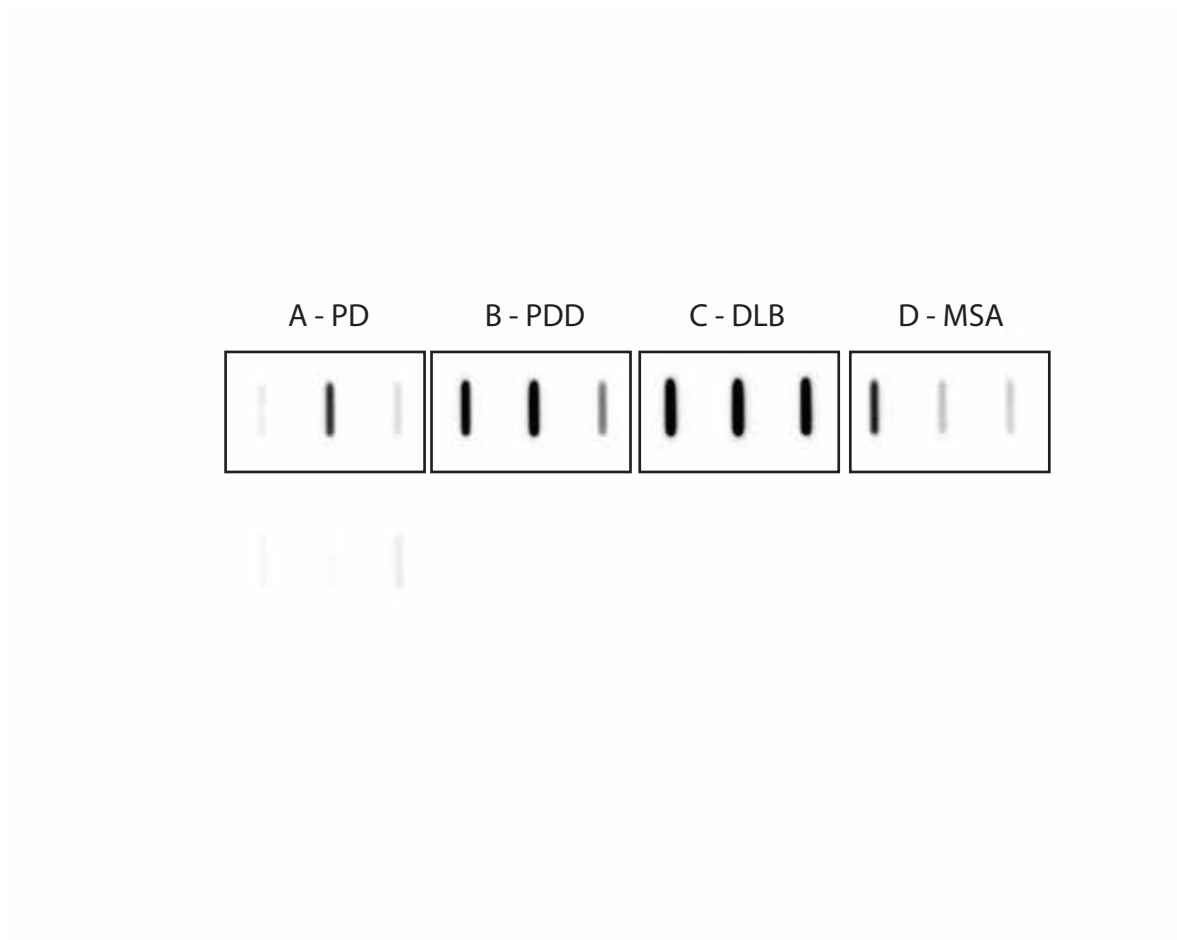

Supplement: Figure 6—figure supplement 1—source data 2. [file elife-92775-fig6-figsupp1-data2.zip › Figure 6 - figure supplement 1_Source data 2/Figure 6-figure supplement 1_Source data 2.pdf]
