# Supplementary material for: Brain-derived and in vitro-seeded alpha-synuclein fibrils exhibit distinct biophysical profiles
Source: eLife. 2024 Nov 25;13:RP92775. doi: 10.7554/eLife.92775 (PMC11588339; doi:10.7554/eLife.92775)

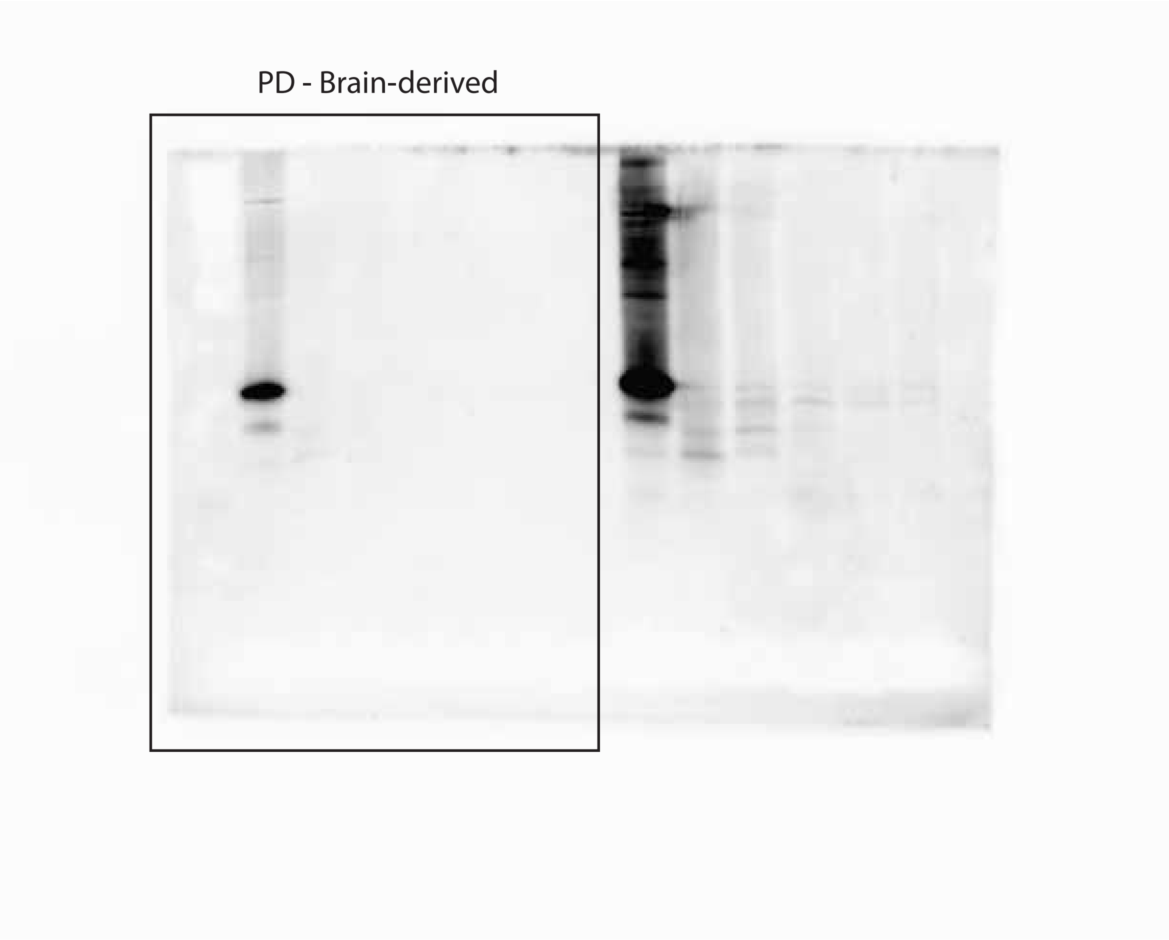
PD - Brain-derived

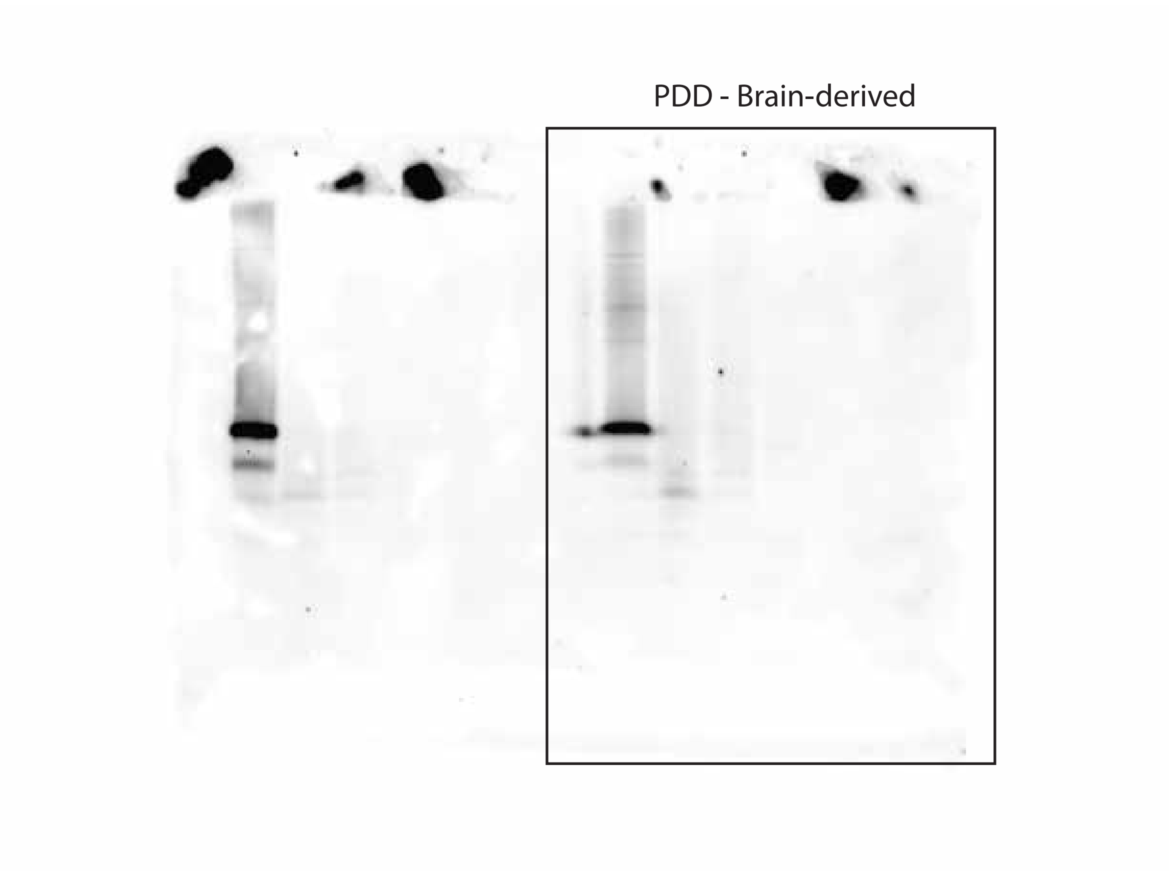
PDD - Brain-derived

DLB - Brain-derived

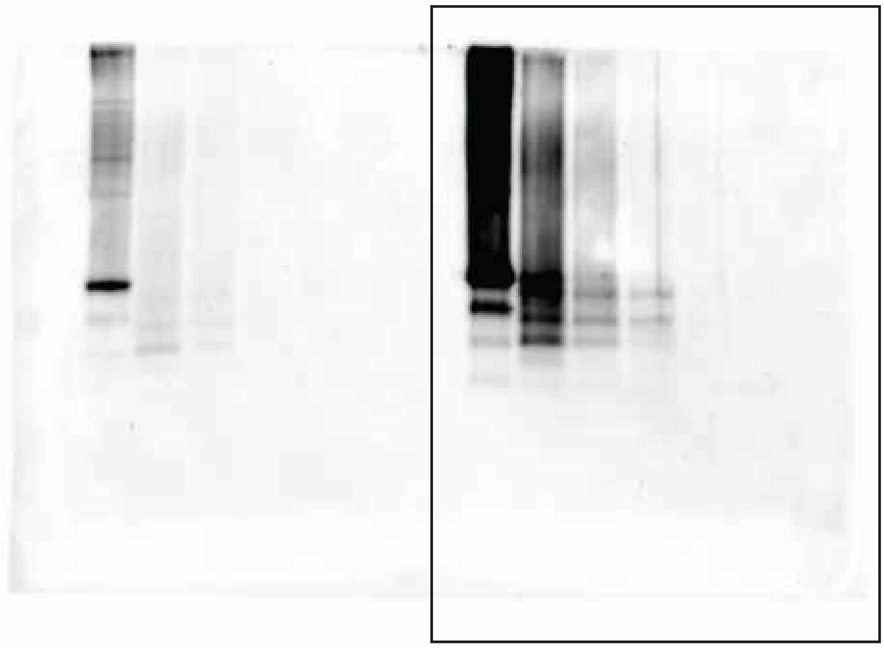

MSA - Brain-derived

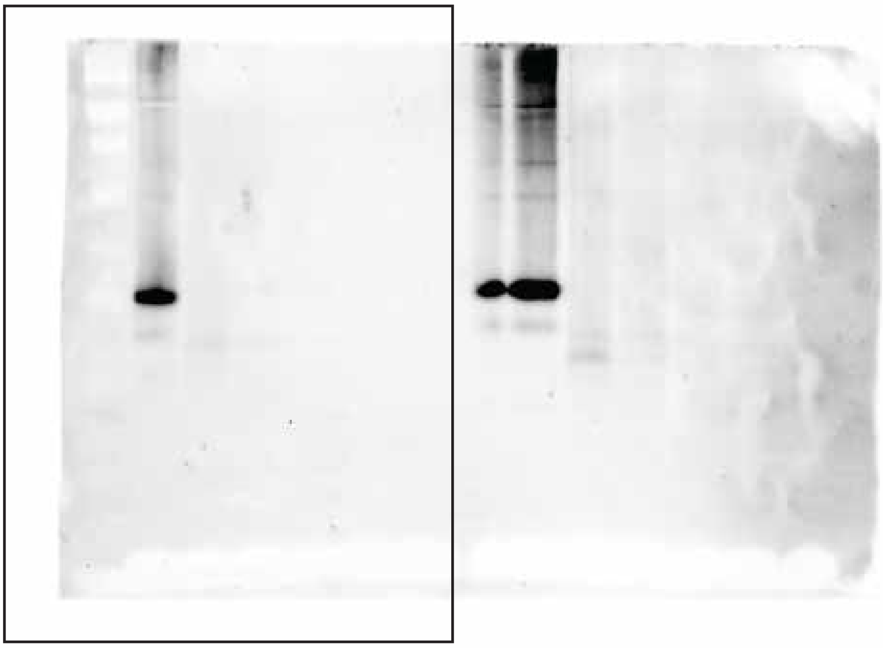

PD - SAA

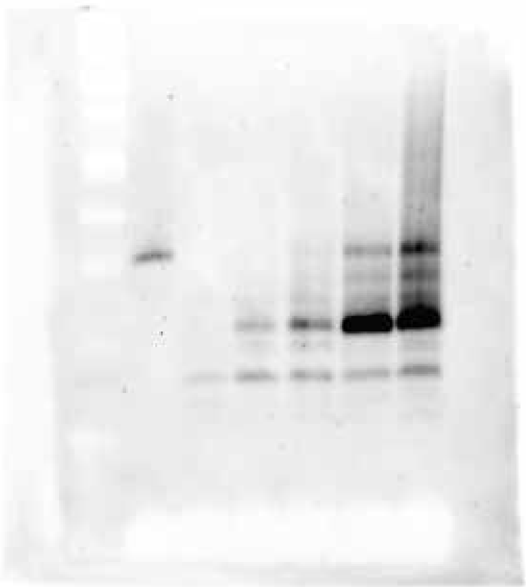

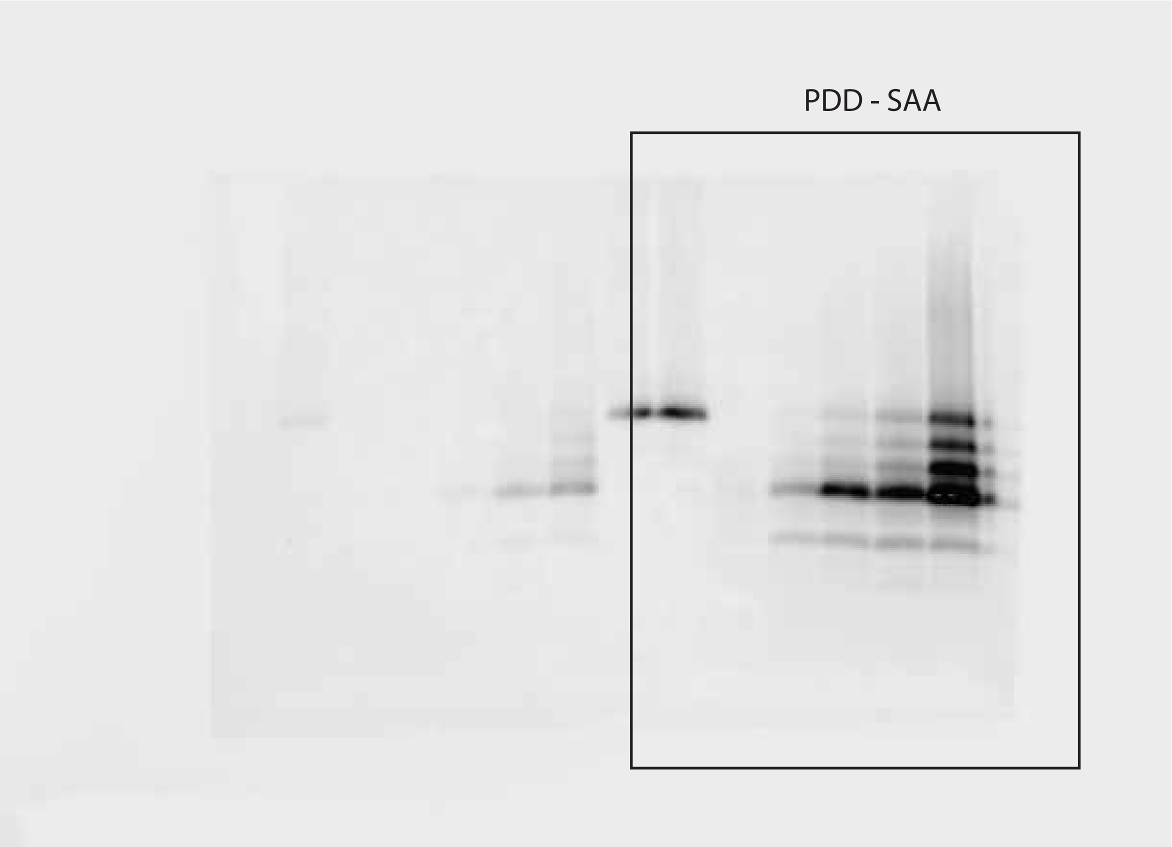

DLB - SAA

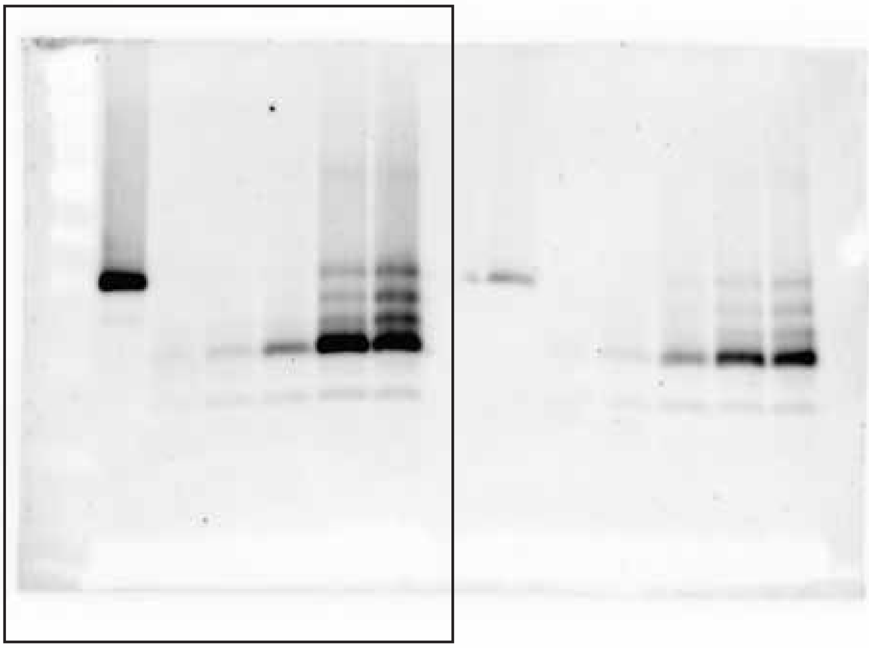

MSA- SAA

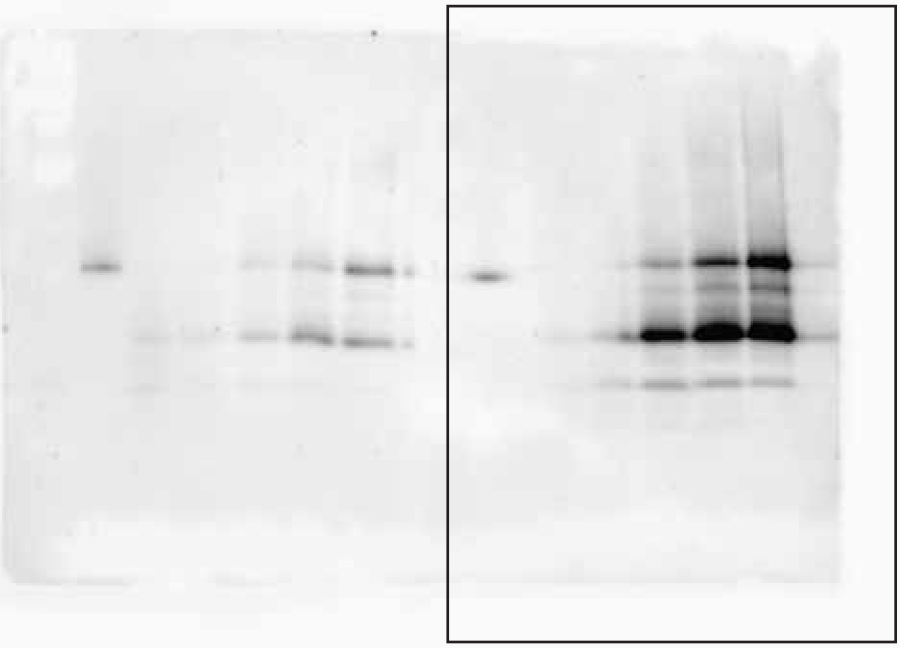

Supplement: Figure 3—source data 2. [file elife-92775-fig3-data2.zip › Figure 3_Source data 2/Figure 3_Source data 2.pdf]
