# Supplementary material for: Brain-derived and in vitro-seeded alpha-synuclein fibrils exhibit distinct biophysical profiles
Source: eLife. 2024 Nov 25;13:RP92775. doi: 10.7554/eLife.92775 (PMC11588339; doi:10.7554/eLife.92775)

Figure 3 - Figure supplement 1 - A

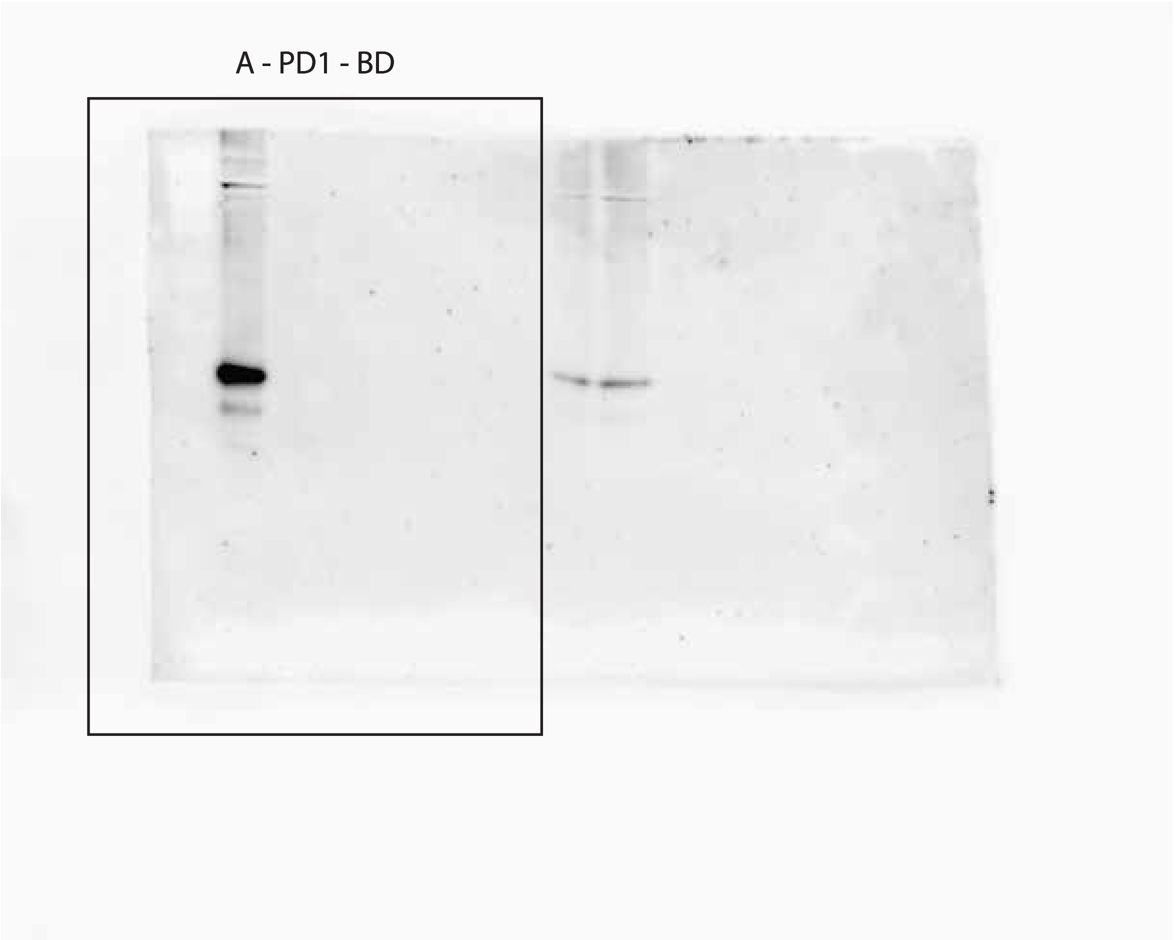

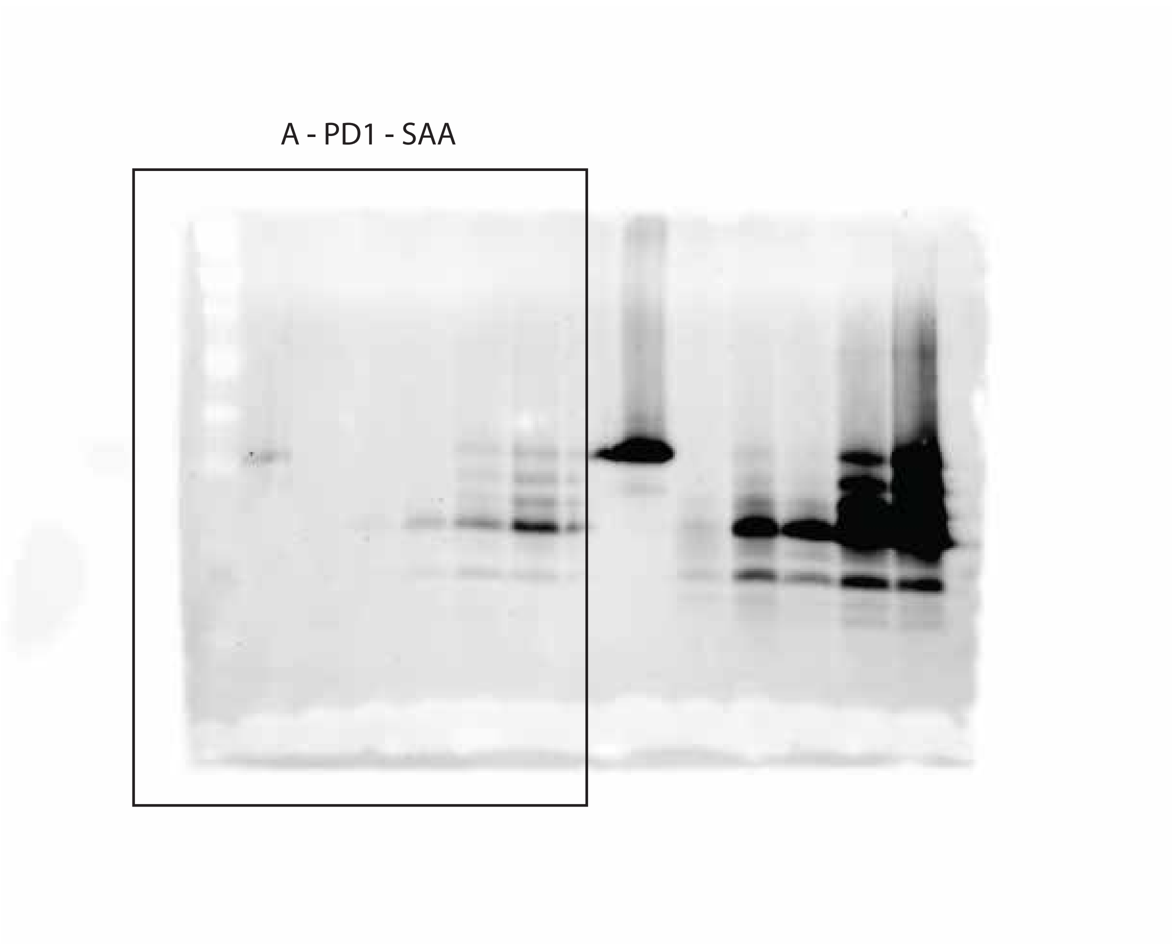

A - PD2 - BD

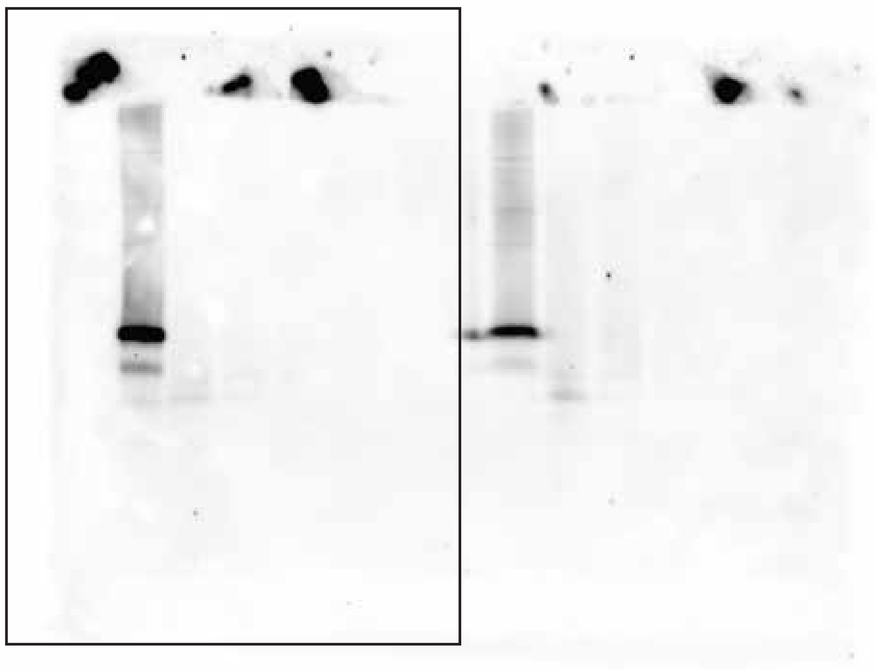

A - PD2 - SAA

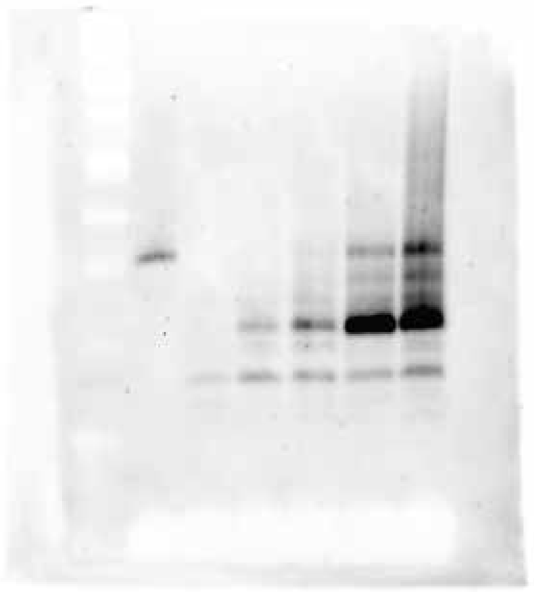

A - PD3 - BD

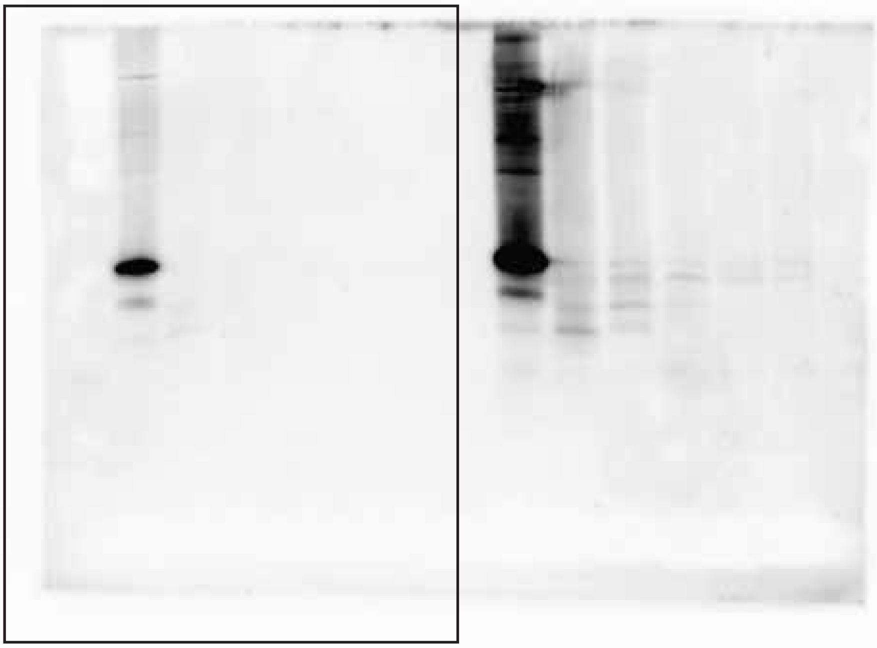

A - PD3 - SAA

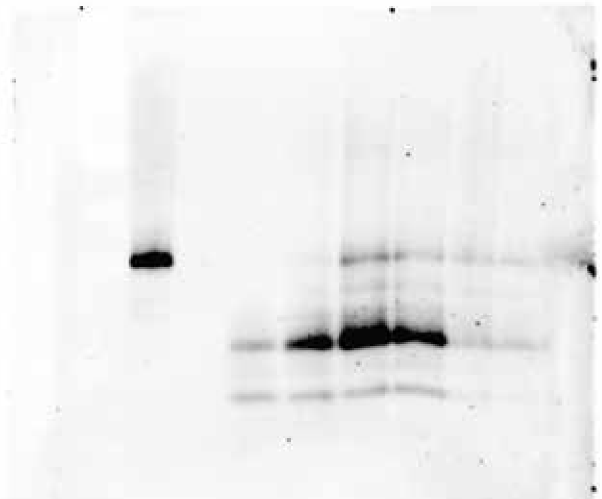

Figure 3 - Figure supplement 1 - B

B - PDD1 - BD

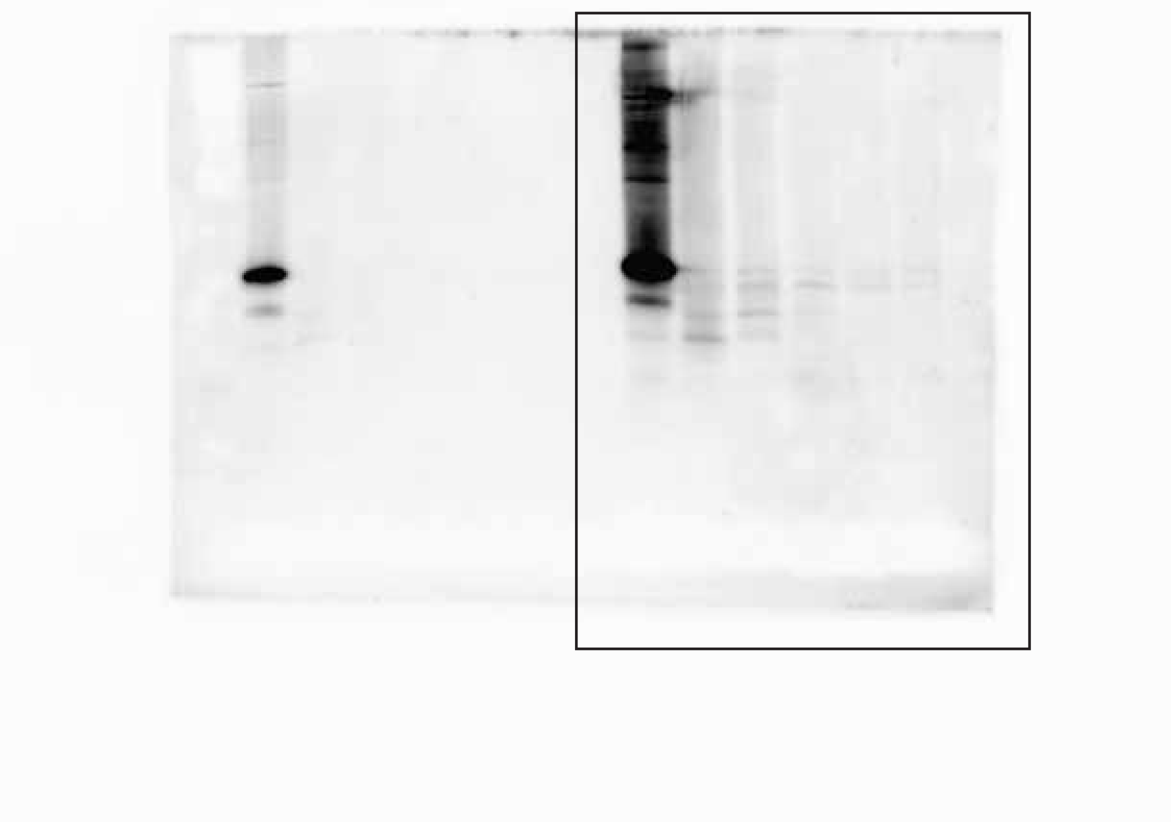

B - PDD1 - SAA

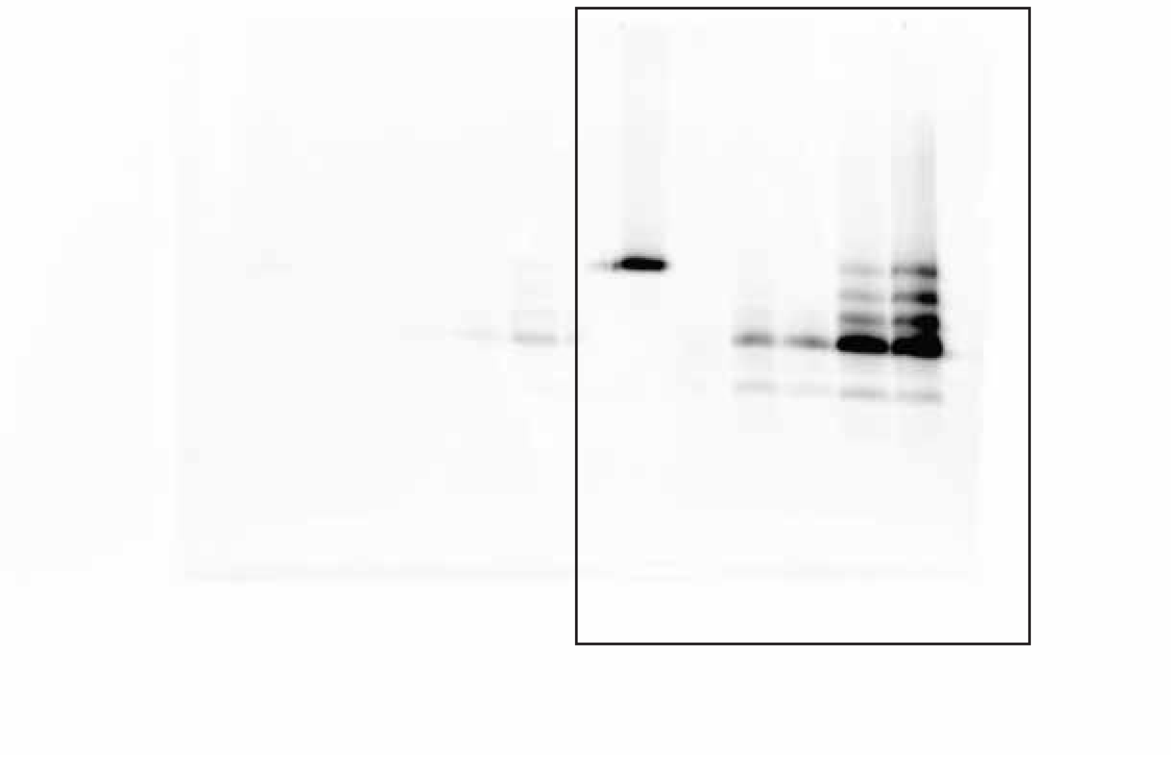

B - PDD2 - BD

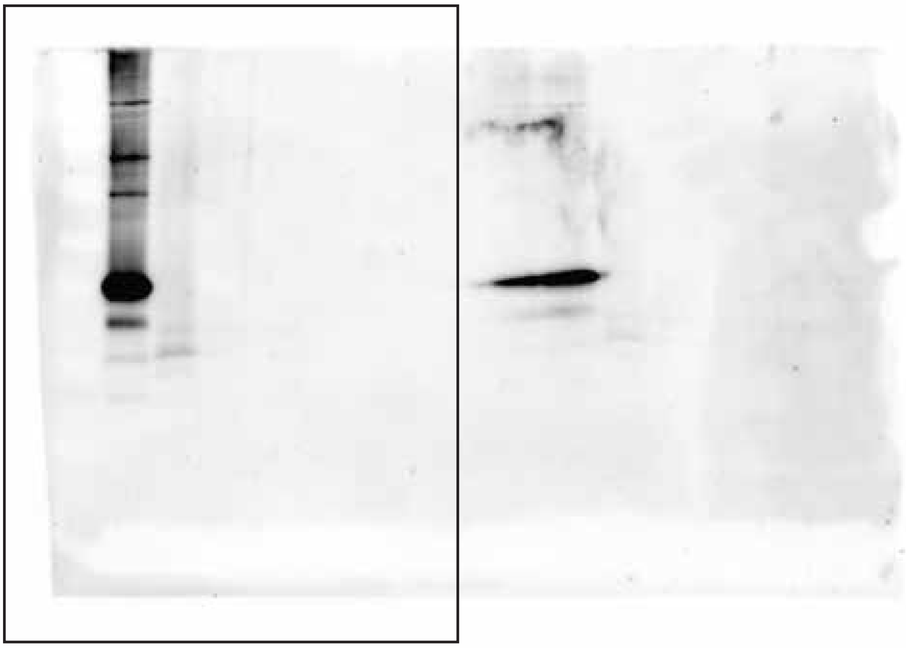

B - PDD2 - SAA

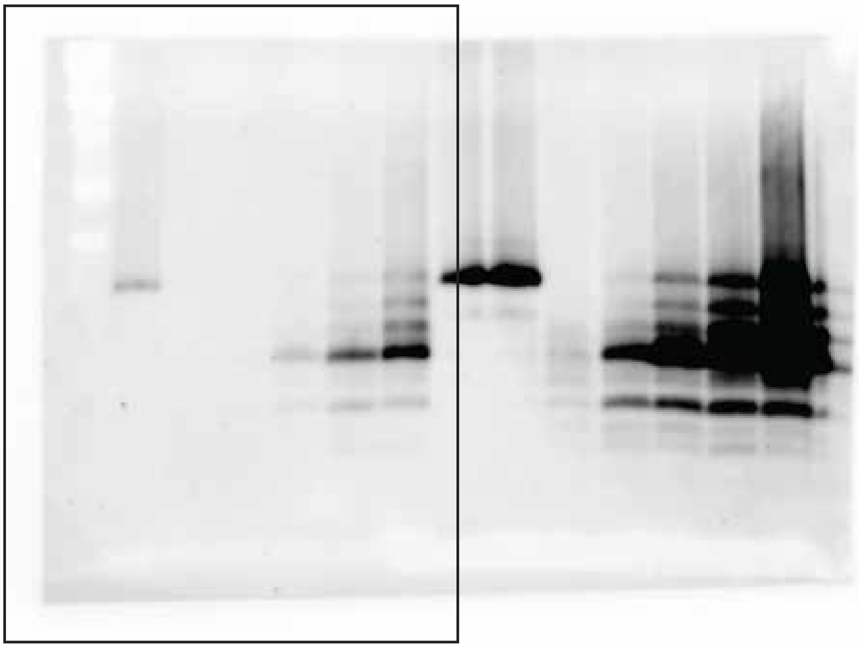

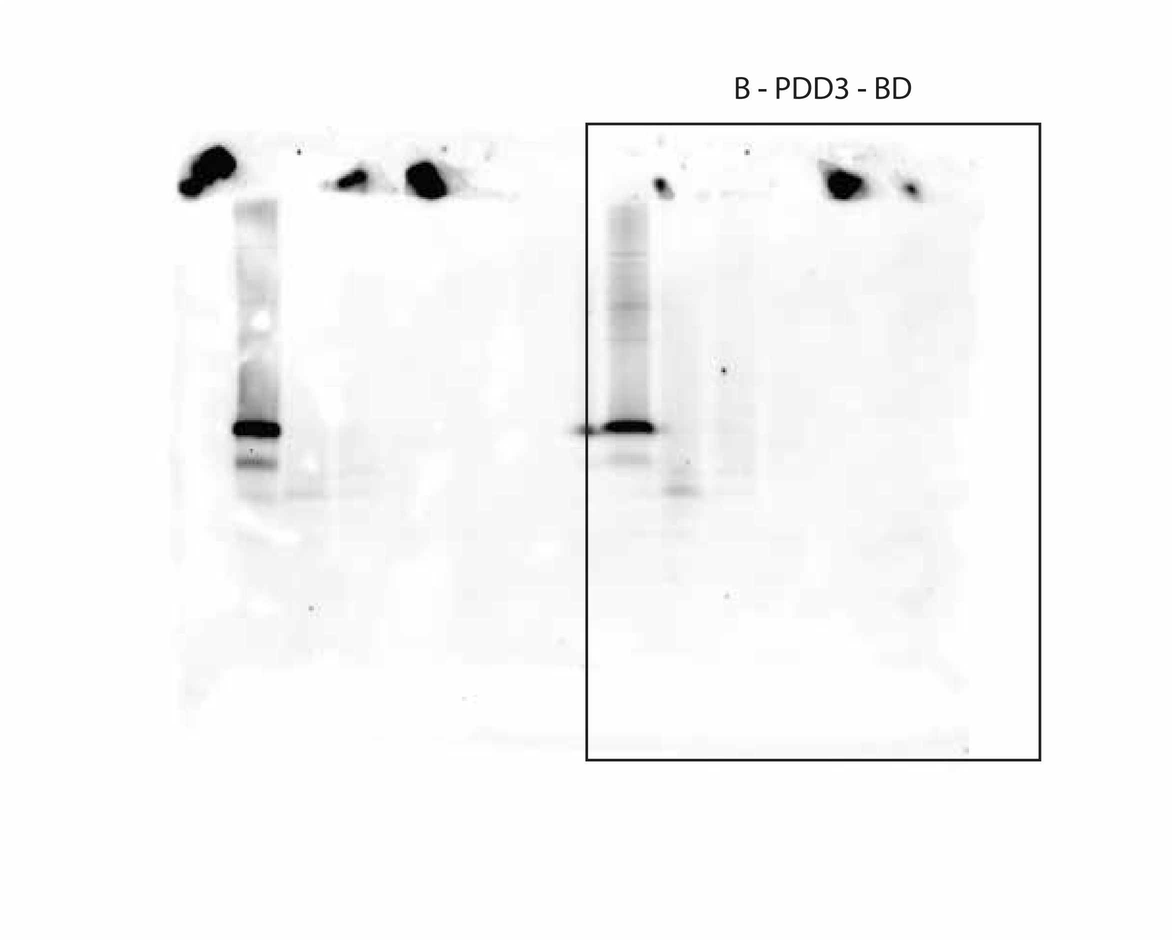
B - PDD3 - BD

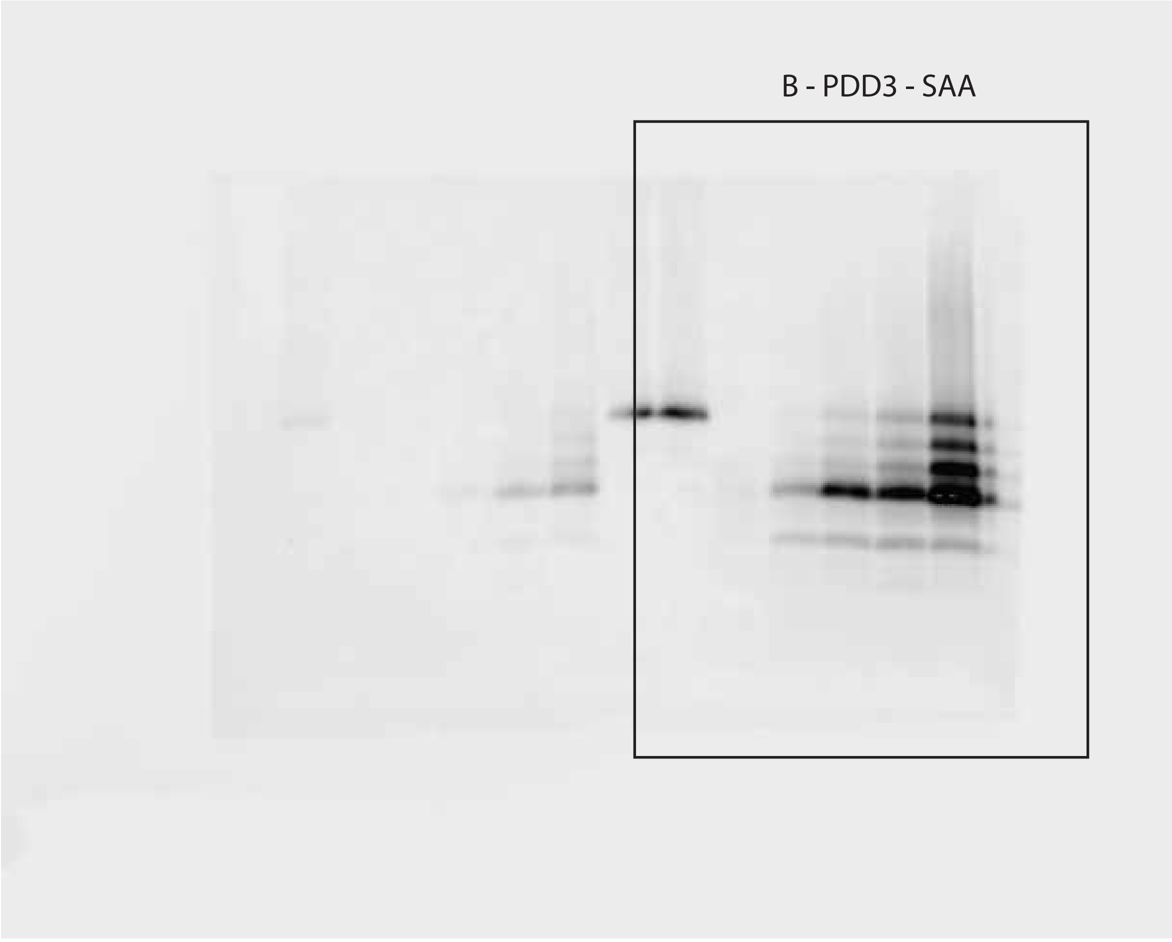
B - PDD3 - SAA

Figure 3 - Figure supplement 1 - C

C - DLB1 - BD

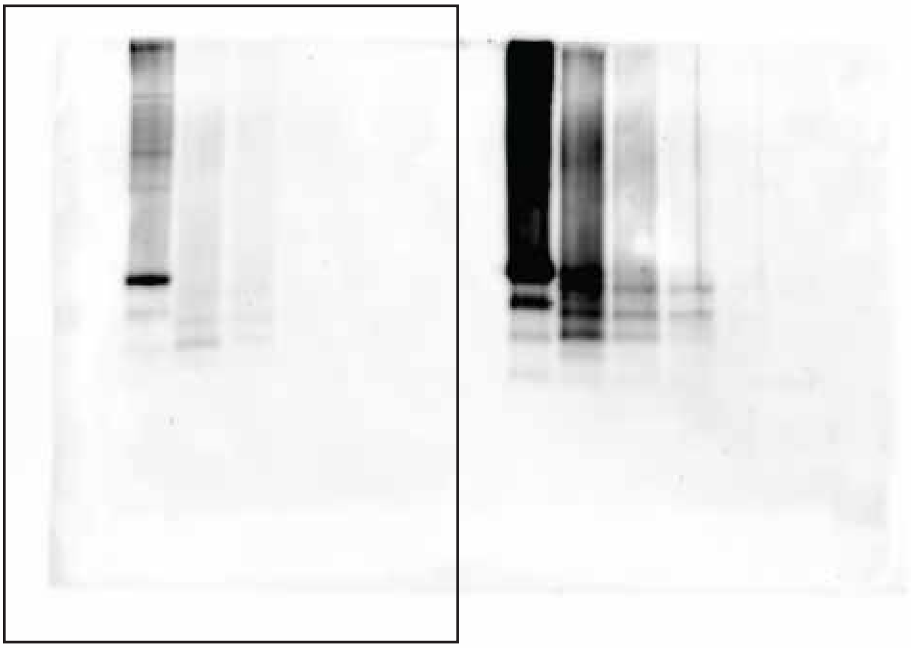

C - DLB1 - SAA

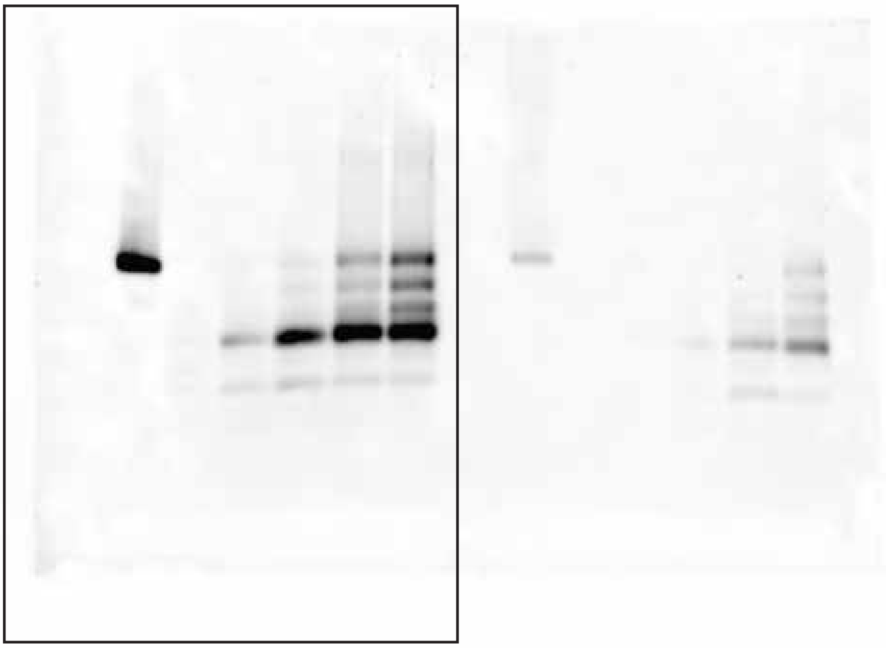

C - DLB2 - BD

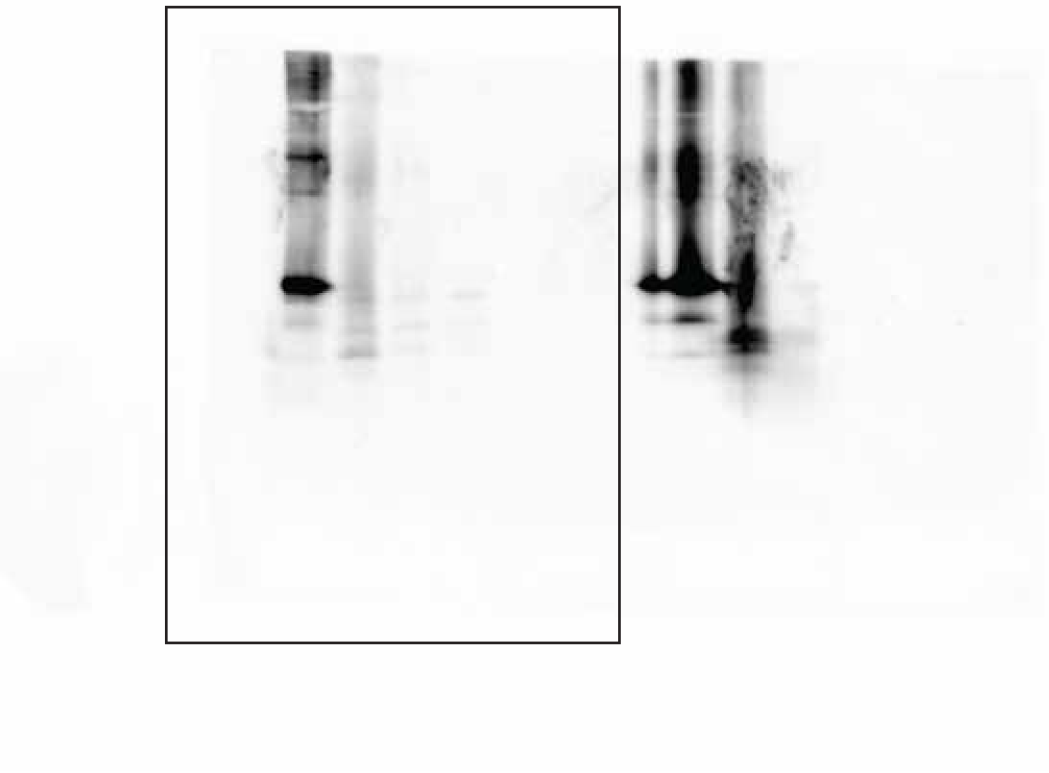

C - DLB2 - SAA

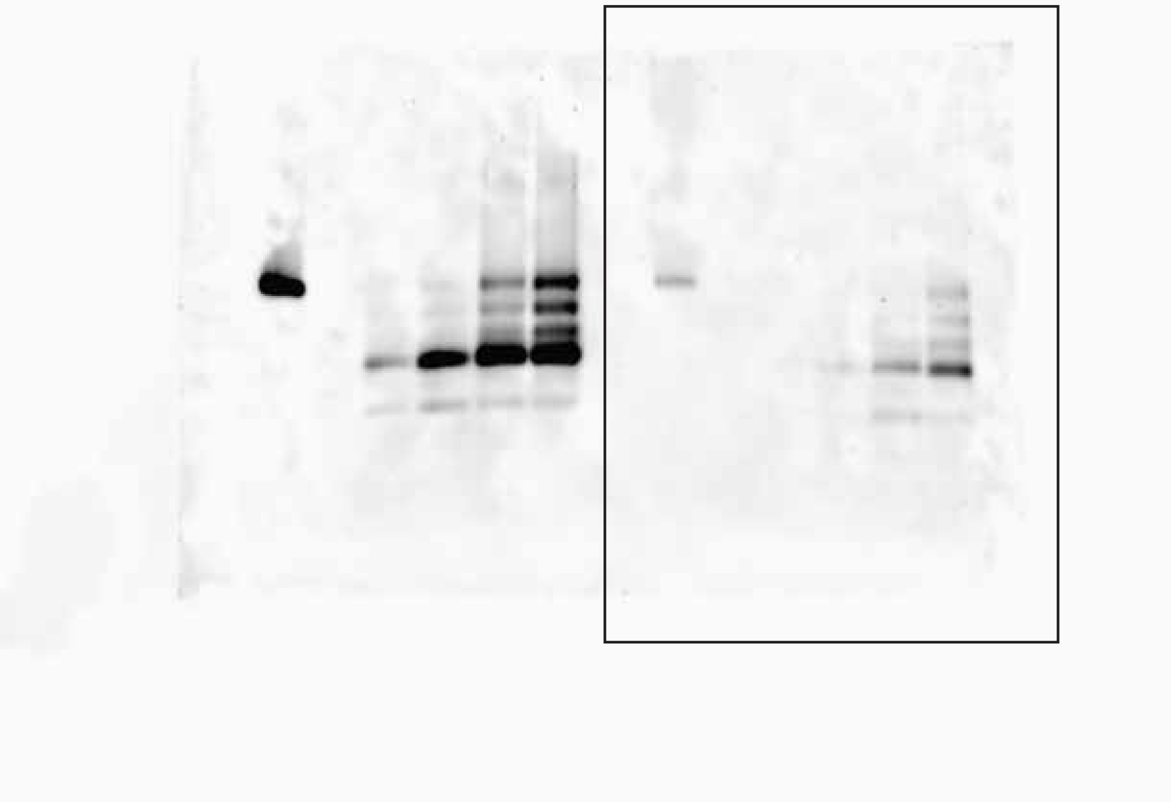

C - DLB3 - BD

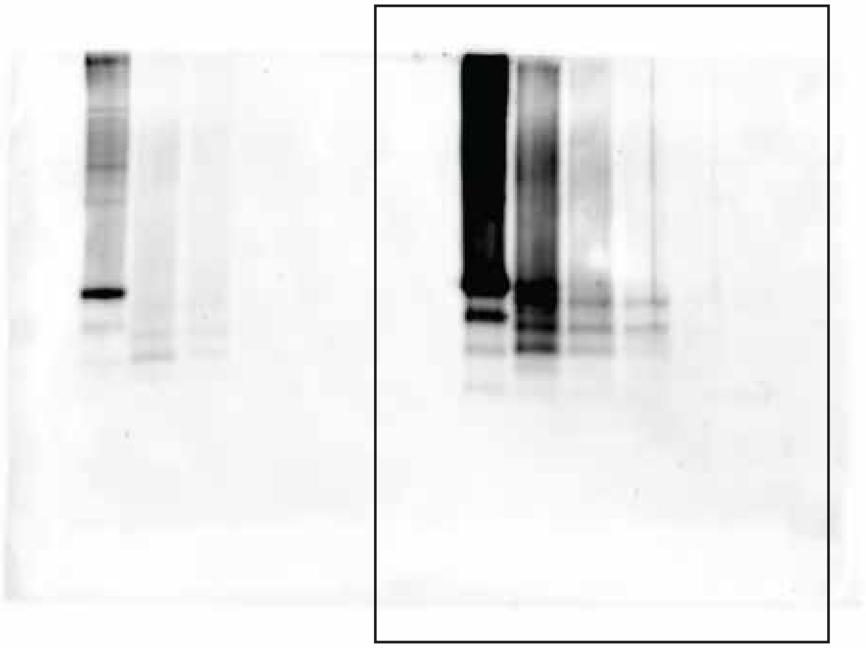

C - DLB3 - SAA

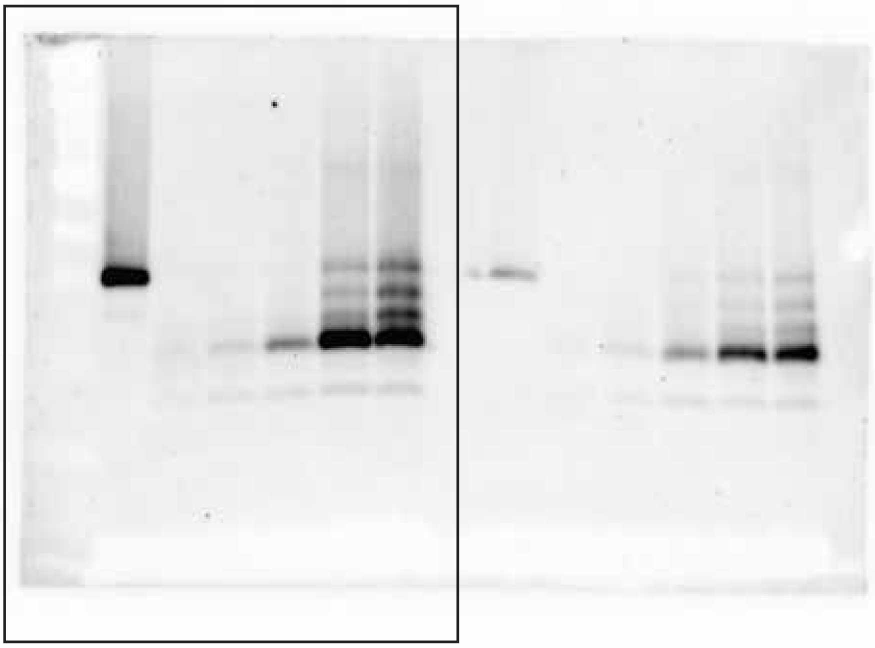

Figure 3 - Figure supplement 1 - D

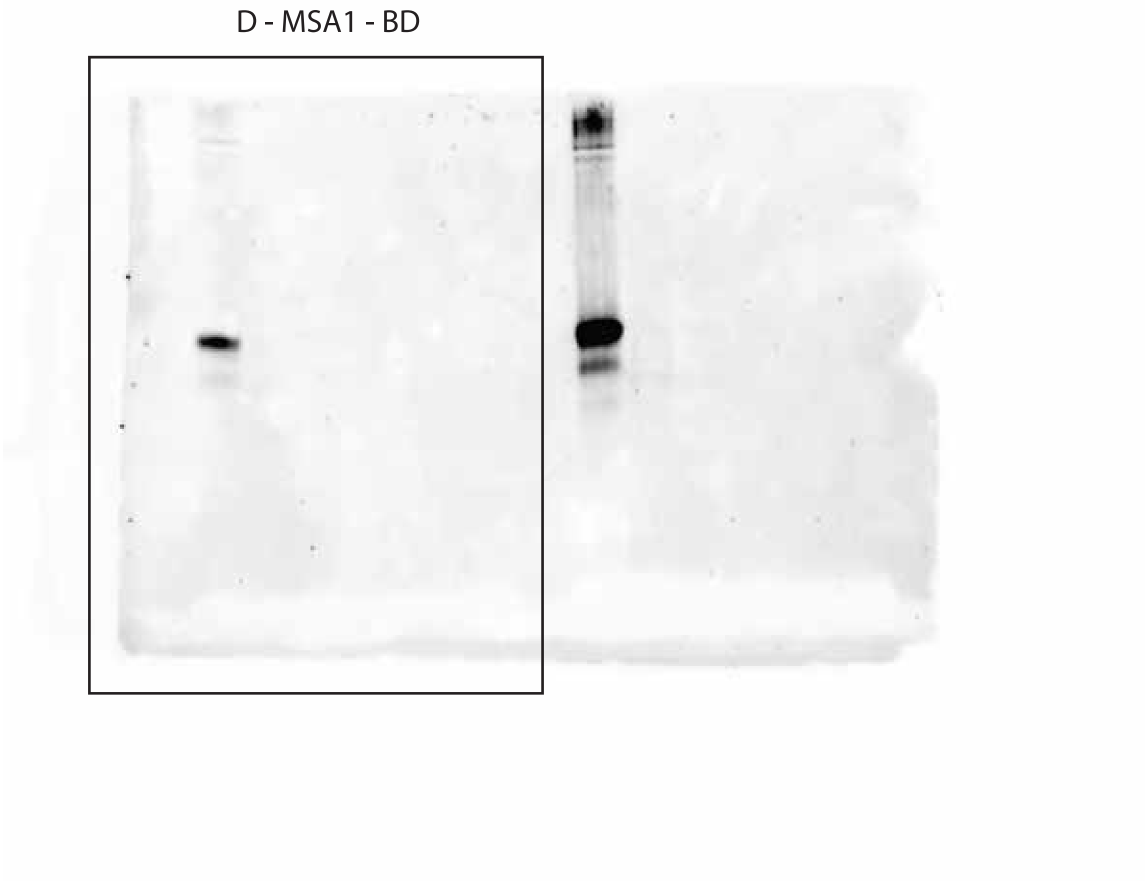

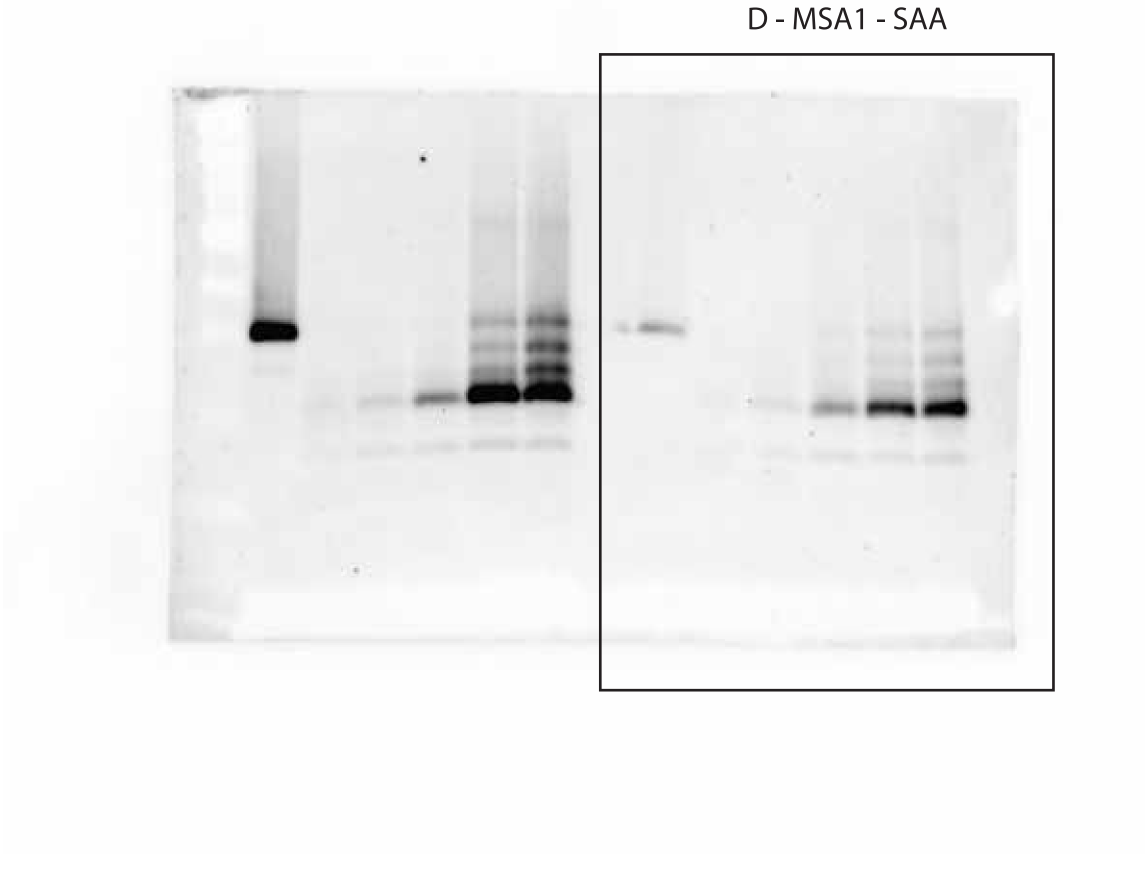

D - MSA2 - BD

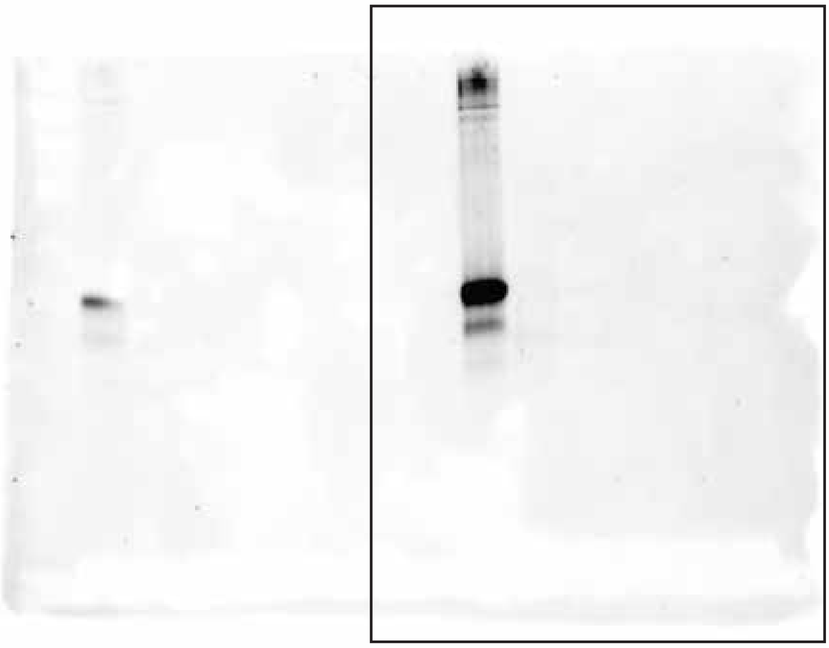

D - MSA2 - SAA

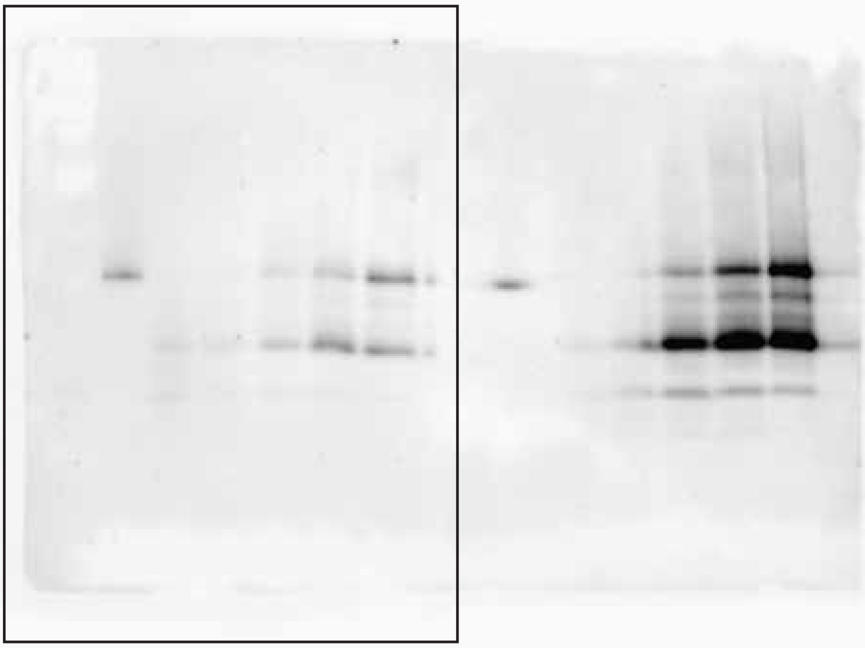

D - MSA3 - BD

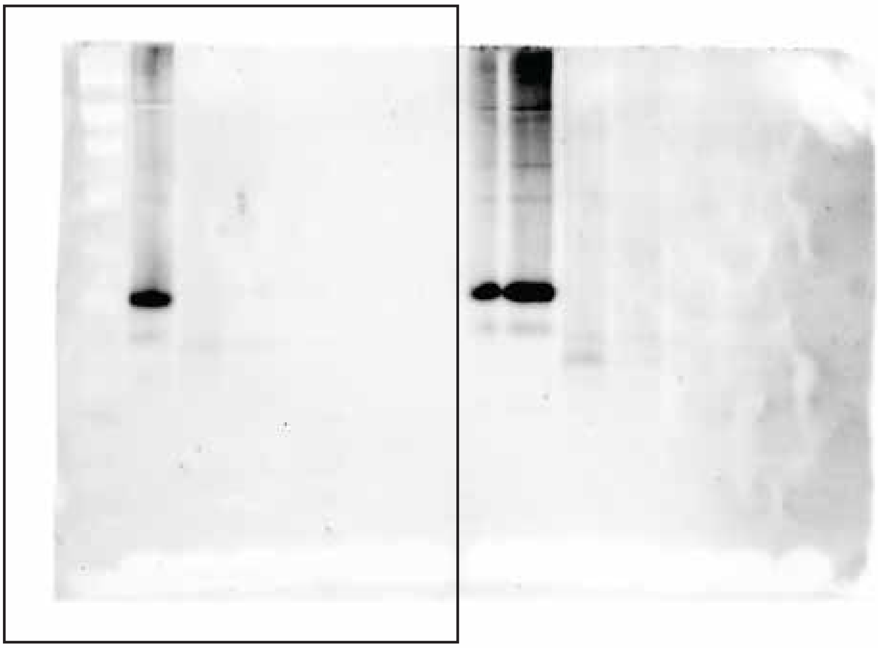

D - MSA3 - SAA

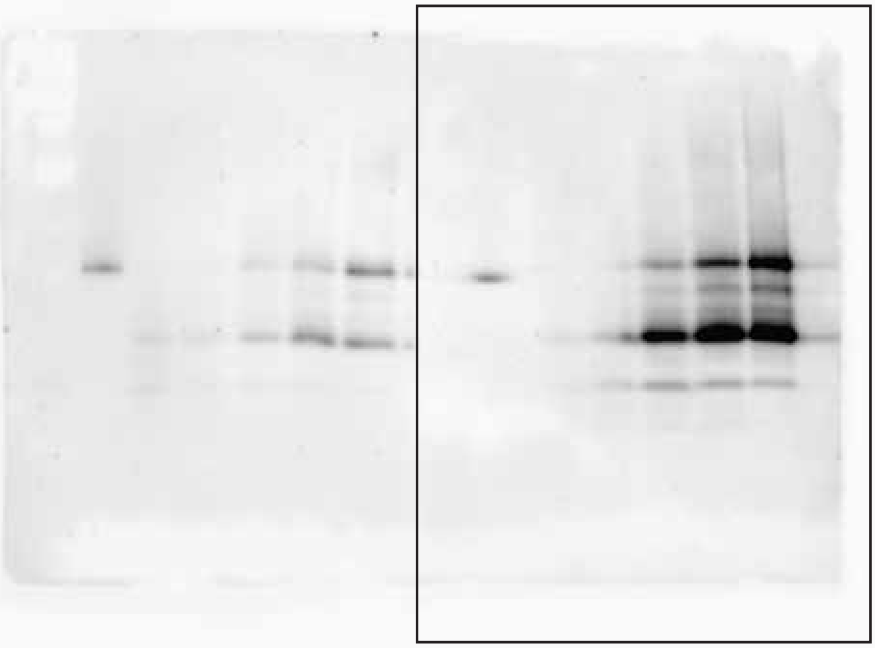

Supplement: Figure 3—figure supplement 1—source data 2. [file elife-92775-fig3-figsupp1-data2.zip › Figure 3 - figure supplement 1_Source data 2/Figure 3-figure supplement 1 _source data 2.pdf]
