# Supplementary material for: Brain-derived and in vitro-seeded alpha-synuclein fibrils exhibit distinct biophysical profiles
Source: eLife. 2024 Nov 25;13:RP92775. doi: 10.7554/eLife.92775 (PMC11588339; doi:10.7554/eLife.92775)
Supplement: Supplementary file 1. — The table summarizes the clinical and pathological reports of the patients included in this study. PD, Parkinson’s disease; PDD, Parkinson’s disease with dementia; DLB, dementia with Lewy body; MSA, multiple system atrophy; HC, healthy control; ECtx, entorhinal cortex; BG, basal ganglia; M, male; F, female; AD, Alzheimer’s disease; CVD, cardiovascular disease; and alpha-synuclein (αSyn). [file elife-92775-supp1.pdf]

Table 1:

Case	Region	Age of death	Sex	Clinical Diagnosis	Disease duration (yrs)	Path Diagnosis	PMI (hr)	Braak aSyn stage	Braak tau stage	CERAD
PD 1	ECtx	86	M	PD	8	PD	20	6	2	0
PD 2	ECtx	78	M	PD	6	PD	31	6	2	1
PD 3	ECtx	90	M	PD	8	PD	14	6	2	1
PDD 1	ECtx	75	M	PDD	25	PDD	20	6	2	2
PDD 2	ECtx	81	M	PDD	10	PDD	11	6	2	0
PDD 3	ECtx	83	M	PDD	16	PDD		6	2	1
DLB 1	ECtx	76	M	Possible AD	7	DLB +AD	52	6	3	2
DLB 2	ECtx	92	F	Possible AD	14	DLB +AD	96	6	3	2
DLB 3	ECtx	86	M	AD + CVD	6	DLB +AD	19	6	3	2
MSA 1	BG	65	M	PD	5	MSA	22	0	0	0
MSA 2	BG	59	M	MSA	2	MSA	24	0	0	0
MSA 3	BG	67	F	MSA	2	MSA	24	0	1	0
HC 1	ECtx	51	M	NA	NA	NA	10	0	0	0
HC 2	ECtx	69	M	NA	NA	NA	48	0	1	0
HC 3	BG	71	F	NA	NA	NA	50	0	1	0
